# Loads Bias Genetic and Signaling Switches in Synthetic and Natural Systems

**DOI:** 10.1371/journal.pcbi.1003533

**Published:** 2014-03-27

**Authors:** Samanthe M. Lyons, Wenlong Xu, June Medford, Ashok Prasad

**Affiliations:** 1 School of Biomedical Engineering, Colorado State University, Fort Collins, Colorado, United States of America; 2 Chemical and Biological Engineering Department, Colorado State University, Fort Collins, Colorado, United States of America; 3 Department of Biology, Colorado State University, Fort Collins, Colorado, United States of America; University of Notre Dame, United States of America

## Abstract

Biological protein interactions networks such as signal transduction or gene transcription networks are often treated as modular, allowing motifs to be analyzed in isolation from the rest of the network. Modularity is also a key assumption in synthetic biology, where it is similarly expected that when network motifs are combined together, they do not lose their essential characteristics. However, the interactions that a network module has with downstream elements change the dynamical equations describing the upstream module and thus may change the dynamic and static properties of the upstream circuit even without explicit feedback. In this work we analyze the behavior of a ubiquitous motif in gene transcription and signal transduction circuits: the switch. We show that adding an additional downstream component to the simple genetic toggle switch changes its dynamical properties by changing the underlying potential energy landscape, and skewing it in favor of the unloaded side, and in some situations adding loads to the genetic switch can also abrogate bistable behavior. We find that an additional positive feedback motif found in naturally occurring toggle switches could tune the potential energy landscape in a desirable manner. We also analyze autocatalytic signal transduction switches and show that a ubiquitous positive feedback switch can lose its switch-like properties when connected to a downstream load. Our analysis underscores the necessity of incorporating the effects of downstream components when understanding the physics of biochemical network motifs, and raises the question as to how these effects are managed in real biological systems. This analysis is particularly important when scaling synthetic networks to more complex organisms.

## Author Summary

Cells rely on complex networks of protein-protein interactions in order to carry out life functions. Scientists believe that these networks are organized in a modular fashion; that is they are made up of functionally distinct parts like an electronic circuit. Modularity implies that just as we put together electronic parts to make an amplifier that we can use in many different circuits, we can put together biochemical reactions to make an amplifier, or a switch or an oscillator, which perform the same function in different organisms. This assumption is important in synthetic biology, where we engineer and assemble synthetic genetic circuits in living organisms in a modular fashion. We show that for important modules like genetic and signaling switches, the assumption of modularity has a crucial limitation. We show that if one simply connects a biological switch to another downstream circuit, the presence of the connection changes the operation of the switch, which in some cases may stop behaving like a switch. Our work underscores the importance of taking into account these downstream connections and suggests that natural systems may be balancing some of these components in order to ensure that despite diversity, modules continue to work in different systems with fidelity.

## Introduction

A longstanding question about signal transduction and gene transcription networks is how modular are they. Here modularity means relative insulation of small subgraphs or motifs of the main network from each other [1]. This question is especially relevant for synthetic biology that aims to build artificial circuits from the bottom up [2]. It is also relevant for molecular biologists that aim to arrive at a quantitative understanding of a cellular decision, by, for example, isolating a crucial network module [3].

For synthetic biologists the challenge is now to move from simple network motifs such as pulse generators [4], genetic switches [5]–[8], logic gates [9], [10], and oscillators [11]–[13] to more complicated networks combining multiple motifs and networks in more complex organisms. Novel applications currently being explored include plant biosensors [14], hazardous waste remediation [15], clean fuel technology [16], and numerous medical applications [17]–[20]. Synthetic biologists hope to utilize biological modules in a manner similar to electrical circuit board components – plugging them together to attain a specific, and novel, function [21]. At the core of the concept of either breaking down complex biological systems into small modules, or even building complex systems from modules, is the belief that these modules will behave predictably in isolation and in connection. Recent theoretical and experimental work however [22]–[25] suggests that the functioning of modules may not be independent of the downstream components that they are connected to. Adding an additional binding reaction to the output of a gene regulatory network (or loading the network) may decrease system bandwidth [24] and substrate sequestration in covalent modification cycles may result in signaling delay [26]. In vitro studies find that there is significant load-induced modulation of the upstream module in an enzymatic signal transduction cascades [24]. Theoretical analysis has also shown that a load can change the fundamental properties of an oscillating circuit [27]. Thus understanding the effects of adding a load to the output of these technologically important network modules is required for a thorough understanding of the challenges of scaling up synthetic networks to higher levels of complexity.

Loads could also have noteworthy unrecognized effects in natural systems. In fact all natural systems have loads in some ways or the other. Motifs in signal transduction networks are connected directly to a transcriptional response, or to downstream proteins that may function as transcription factors or go on to activate transcription factors. Motifs in gene transcription networks have transcriptional outputs with protein domains that bind nonspecifically and specifically to binding sites on the DNA, apart from interacting with other transcription factors.

Circuits that function as switches play an important role in all biological signaling and gene transcription networks because they encode decisions. This change of state can be brought about by an external signal, or an internal accumulation of a protein, which can drive the system to a different steady state. Examples are the regulatory circuits for the cell cycle in yeast [28], mitogen-activated protein kinase cascades in animal cells [29]–[31], and the lysis-lysogeny switch in the λ phage [32]. Since many small circuits can show this kind of behavior, switches are among the earliest and most well studied of protein interaction circuits [33]. The genetic toggle switch, which was one of the first two synthetic circuits constructed, is a well-known synthetic example [5]. Given the ubiquity and importance of switch-like motifs, it is important to understand how their function could be affected by binding downstream partners.

**Figure 1:**
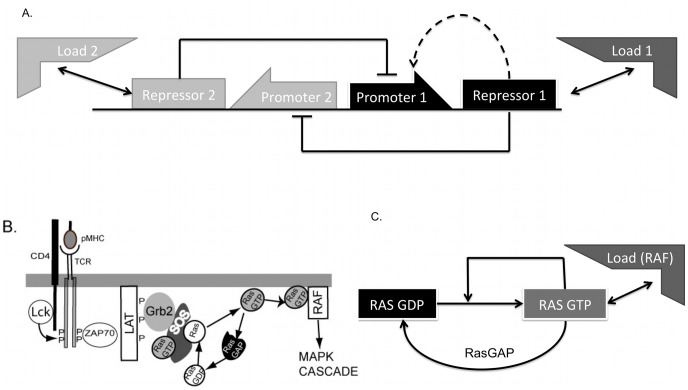
Schematic diagram of the circuits studied in this paper. (A). The basic toggle switch is the network shown without the dotted line. Repressor 1 represses the production of Repressor 2 and vice versa. The dotted line denotes a positive feedback motif found in some natural circuits. (B). A cartoon of part of the MAPK activation pathway in T lymphocytes, adapted from [29], showing the role of Ras activation. Signals from peptide-MHC complexes are received at the TCR and lead to phosphorylation of the cytoplasmic chains of the TCR by the Src kinase, Lck. This recruits the kinase ZAP70 which trans-autoactivates and phosphorylates a scaffold called LAT, which recruits Grb2 and SOS to the plasma membrane. SOS activates Ras as as shown. (C) A simplified model of the Ras switch. RasGDP transforms into RasGTP via the enzyme SOS. However the catalytic rate of SOS increases when bound to RasGTP. This sets up an autocatalytic positive feedback. RasGTP is deactivated by enzymes called RasGAP's (among others).

These reasons prompted our theoretical study of the behavior of a simple genetic toggle switch [5], a toggle switch with positive feedback as well as a common positive-feedback based switch involving Ras activation in lymphocytes [29], [30] under a load on either one or both of its outputs. These circuits are shown in Fig. 1 and described below. The simple toggle switch is a widely studied and emulated synthetic network motif based on the mutual repression of two repressor proteins. However, naturally occurring toggle switches are often found connected to an additional positive autoregulatory component. For example in the competence system in *B. subtilis*, ComK represses the production of Rok and Rok represses the production of ComK; however ComK also has a strong positive feedback upon its own production [34]. Another example is found in the apoptosis network of many multicellular organisms, including mammals. Within the pathway controlling intrinsic apoptosis is a set of genes with double-negative repression, Casp3 and XIAP, again accompanied by positive autoregulation of Casp3 [35].

The Ras protein is a G-protein found on mammalian cellular membranes that is important in many cellular processes and is an upstream activator of the MAPK pathway. Ras goes from a GDP-bound inactive form to a GTP-bound active form, often in a digital manner [30], and previous studies in lymphocytes have shown that RasGDP is activated to RasGTP via a bistable switch that arises from a positive feedback loop on its own activation via SOS (Son of Sevenless) [30]. However the Ras switch very naturally has an associated load, since to transduce the cellular signals down along the MAPK/ERK pathway, RasGTP naturally binds to Raf kinase. Thus the Ras switch system contains all the elements we need to study the effects of adding loads to a bistable switch which is based on a positive feedback loop.

## Methods

### Genetic toggle switch

The basic genetic toggle switch consists of two mutually repressing genes as shown in Fig. 1 along with an additional system to toggle the states. As shown in previous studies, with the right combination of parameters, the toggle switch will stay in one of two stable states, each characterized by a high concentration of one of the repressor proteins, and strong repression of the other. The toggle switch can now be induced to switch states using two possible strategies for inducing a transition: decrease the level of highly expressed protein [5], [36] or increase the expression of one of the repressed proteins (Fig. 1) using an additional inducible system [36]. For a model which utilizes the latter protocol we obtain a system of four differential equations [36] after including a load. The load may be a protein, a small molecule or a binding site on DNA such that the bound complex prevents the repressor from binding to and repressing its conjugate promoter. In order to make the simplest and the most general model, we have assumed here that the repressors reversibly bind the load only in one copy. We assume that the total load L_1T_ is a constant, L_1_ is the free load and conservation gives us the bound load as L_1T_−L_1_.
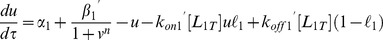
(1)

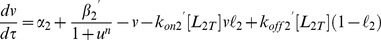
(2)

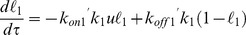
(3)

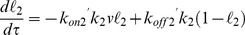
(4)


These four equations are presented in de-dimensionalized form, with 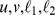 representing the dimensionless concentrations of Repressor 1, Repressor 2, Load1 and Load2 respectively and τ the de-dimensionalized time. The basal parameter values that we use are as follows: α_1_ = α_2_ = 0.2; β_1_′ = β_2_′ = 4; n = 3; k_on1_′ = k_on2_′ = 0.5; k_off1_′ = k_off2_′ = 0.5; k_1_ = k_2_ = 1; [L_1T_] and [L_2T_] are variable. Note that Equations (1) and (2) without the last two terms incorporating the load are the standard equations for analyzing the toggle switch that have been widely used in both empirical and theoretical work [5], [36]. These equations are discussed in more detail in Supplementary Text S1 Section 1.1. The derivation of this model follows that of Kobayashi et al [33]. All parameters excluding load binding rates were sourced from Kobayashi et al [36]; extensive parameter sensitivity of the load binding rates was performed and are discussed in the Supplementary Text S1 section 1.4 and Figs. S1, S2, Table S1 and Figs. S15 and S16. The effect of a load arises from the binding competition between the promoter where the repressor binds and the load. This competition is not directly incorporated into the Hill function, since the binding step with the promoter is not explicitly modeled and is treated in an effective way. In reality however the concentration of the promoter is so small compared to that of the load, that the use of Hill functions is justifiable [37]. There are possibly exceptional cases such as a high copy number of plasmids compared to load concentrations where this assumption does not apply. Note that the Hill function is an effective phenomenological equation describing gene transcription and protein production, and standard Law of Mass Action (LMA) methods to derive the Hill functional form may not apply for many transcription factors that nevertheless show Hill kinetics [38]. Thus it is preferable to use Hill function forms for this analysis.

To calculate transition times, we first start the system in one state, say high Repressor 1. After the system has reached steady-state, we add a constant concentration of the inducer and measure the time taken for Repressor 2 to go from 10% of its maximum value to 90% of its maximum value. This is the “rise time”. Similarly the “decay time” is the time taken for Repressor 1 to go from 90% of its maximum value to 10% of its maximum value. The level of the inducer remains fixed.

In practice the inducer may decay and the transition would depend upon there being inducer present for a sufficiently long time to induce transition. In such cases the amount of inducer required may be of interest. When the inducer is applied as a bolus with a first order decay rate, it appears as an exponentially decaying pulse. We thus included a fifth differential equation governing the amount of Inducer.
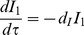
(5)


Here 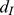 is the ratio of the inducer degradation constant to the repressor degradation constant. We used Eq. 5 only when estimating the amount of inducer required to switch states for different loads and different decay rates of the load (Supplementary Text S1 section 1.4 and Supplementary Tables S3, S4).

A genetic toggle switch can be induced to change states by the alternative method of repressing the highly expressed repressor, and in fact the original toggle switch used this form of induction [5], [33]. We repeated our calculations for the basic model for the case of alternative induction, but found no qualitative differences. The alternative induction model along with the equations is detailed in the Supplementary Text S1 section 1.4.

Equations 1–4 assume that the load itself stays in steady state during the switching of the toggle between one state and another. However in reality if the load is another protein, it is also synthetized and degraded by the cell, and therefore its level could be dynamic. We also simulated this situation by incorporating a synthesis and a degradation rate for each load. This resulted in Equations 3 and 4 being replaced by:
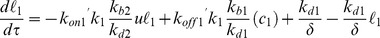
(6)

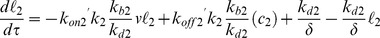
(7)


Here 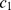 is the load-repressor complex and 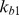 and 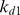 are the synthesis and degradation rates respectively for Repressor 1, and correspondingly for Repressor 2. The parameters are defined in the Supplementary Text S1, section 1.5. Since the total load is no longer conserved, we need to include additional equations for the load repressor complex.
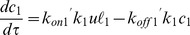
(8)

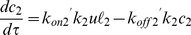
(9)


Our model assumes that when the repressor protein is bound to the load, it is protected from degradation. However it is also possible that even when the protein is bound to the load, it can still degrade. To check the impact of removing the protection assumption, we also consider an additional model where the repressor can still degrade with the same rate constant when bound to the load. The equations for that model are slightly modified versions of the equation above, and are presented in detail in the Supplementary Text S1, section 3.2.

We conducted parameter sensitivity analysis on models utilizing both forms of induction; these did not show any qualitative change on wide variation of key parameters (Tables S1, S2, S3, S4 and Supplementary Text S1).

### Toggle with positive feedback

A positive feedback was added to the R1 side of the toggle switch as an inducible promoter with a Hill coefficient of 1. We assumed that the positive feedback acted on the same promoter as the repression, resulting in a composite term for production of R1 from promoter 1 where ρ is the strength of positive feedback.
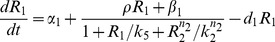
(10)


The derivation of this equation can be found in the Supplementary Text S1, section 1.6.1. As before, α_1_ is the leaky production of R_1_ while α_1_+β_1_ represents the activity of the promoter in the absence of repression or positive feedback. We chose k_2_ and k_5_ = 1, d_1_ = 0.2, and for the figures in the main paper we chose ρ = 3.5. We address other values of the positive feedback in Fig. S6 and the Supplementary Text S1, section 1.6.2.

### Stochastic simulations

We perform stochastic simulations and histogram the concentrations of the repressor proteins to construct their probability distribution. The quasi-potential of the toggle is given by the negative logarithm of this probability distribution [39]. In order to construct the probability distribution we make use of the phenomenon of noise-induced switching. Recent theoretical work has shown that multiplicative noises due to stochastic fluctuations can induce switching [40]–[42]. Experimental results demonstrate bimodal populations that correspond with theoretic predictions arising from noise-induced switching [41].

Stochastic simulations were carried out using a modified Gillespie algorithm using the standard rate expressions for every reaction (Table S5). We chose a reaction volume that would correspond to a small number of molecules in the system. Stochastic fluctuations then drive the system to transition between states rapidly, allowing us to collect sufficient data points. In order to make sure that the system was not being biased by the small volume, we also repeated the calculations for a five times larger volume (and hence molecule number) and found qualitatively similar results (Fig. S4).

For the positive feedback toggle switch the same equations were used except for the repressible production of Repressor 1, where we used instead the rate expression given by the right hand side of Eq. 10 in the Monte Carlo simulations.

### Ras-kinase system

For our study we adapted the minimal model of the Ras switch proposed by Das et. al. [30] with the addition of a reversibly binding load in the form of the Raf protein (Fig. 1C). The model contains three proteins, Ras, which exists as RasGDP or RasGTP, SOS, the guanine exchange factor (GEF) that catalyzes the transformation from RasGDP to RasGTP and a GTPase, RasGAP. SOS on its own has very low GEF activities. However, the activity of the GEF pocket is strongly influenced by the binding state of an allosteric pocket in Cdc25 domain [29], [30]. When the allosteric pocket is bound by RasGDP, the GEF activity is increased by 5 times. If the allosteric pocket is bound by RasGTP, its GEF activity is increased by 75 times. In this way, RasGTP can upregulate its own production rate by binding to SOS, thus constituting a positive feedback loop. RasGTP is deactivated by GTPase's such as RasGAPs that are constitutively present.

After Raf binds RasGTP, the complex catalyzes the phosphorylation of Raf leading to a phosphorylation cascade. For this study we ignore Raf activation and only consider the effects of Raf as a binding partner for RasGTP. The Das paper [30] also models the systems using Michaelis-Menten (MM) forms for the actions of the enzymes which is quite standard for modeling systems of enzymatic reactions. However since in this model the load competes not with a promoter, as in the toggle switch, but with another protein, it is possible that the quasi-steady state assumption of the MM form could be introducing some inaccuracies in the results. To account for this possibility we wrote the entire model using the Law of Mass Action. We separately simulated the model using the MM functional forms (Supplementary Text S1 section 2 and Figs. S7 and S9). The equations for the MM forms are listed and discussed in detail in the Supplementary Text S1. The reactions and rate constants for this model are listed in Table S6 and Table S7.

We use the following notations for the species involved in the system:
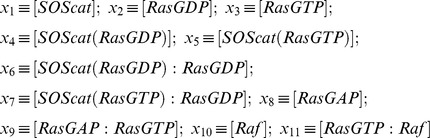


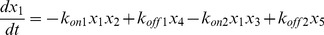
(11)

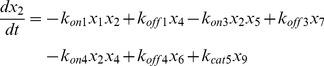
(12)

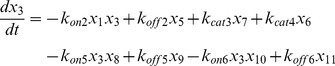
(13)

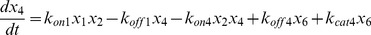
(14)

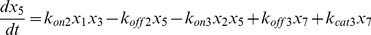
(15)

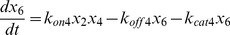
(16)

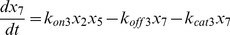
(17)

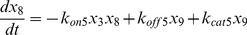
(18)

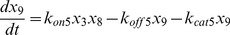
(19)

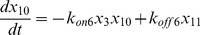
(20)

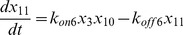
(21)


Moreover, four of the basic protein species along with the complexes they participate in have associated conservation laws. These are as follows:
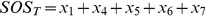
(22)

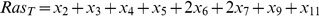
(23)

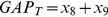
(24)

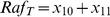
(25)


In the Ras model too we implicitly assume that when RasGTP is bound to Raf, it is protected from de-activation by a RasGAP. We also study the effects of relaxing this assumption on both the LMA and the PSSA models. The modifications to the original model are detailed in the Supplementary Text S1 section 3.3.1.

We used XPPaut to perform a bifurcation analysis of the Ras switch with changing levels of SOS, with and without a load. The quasi-potential landscape does not provide useful insights into load induced modulation of the Ras switch and hence the probability distributions are not reported.

## Results

### The bistability properties of the toggle switch do not change unless the repressor can degrade when bound to the load

The presence of a binding partner for either Repressor 1 or Repressor 2 (which we refer to thereafter as the load) introduces new terms in the differential equations describing the toggle switch, i.e. the last two terms in Eq. 1 and in Eq. 2, as well as two new equations, Eq. 3 and 4, in the dynamical system. However it can be easily seen that in steady state Eq. 3 and 4 are also independently set to zero, and therefore do not affect the bifurcation properties of the switch. Even in the case of a dynamic load, since Eq. S13 and S14 are set to zero to ensure the load-repressor complex is in steady state, the additional terms in Eq. S9 and S10 are also zero. Thus the load makes no difference to either the bistability of the switch or to the parameter values where the bistability is seen.

**Figure 2:**
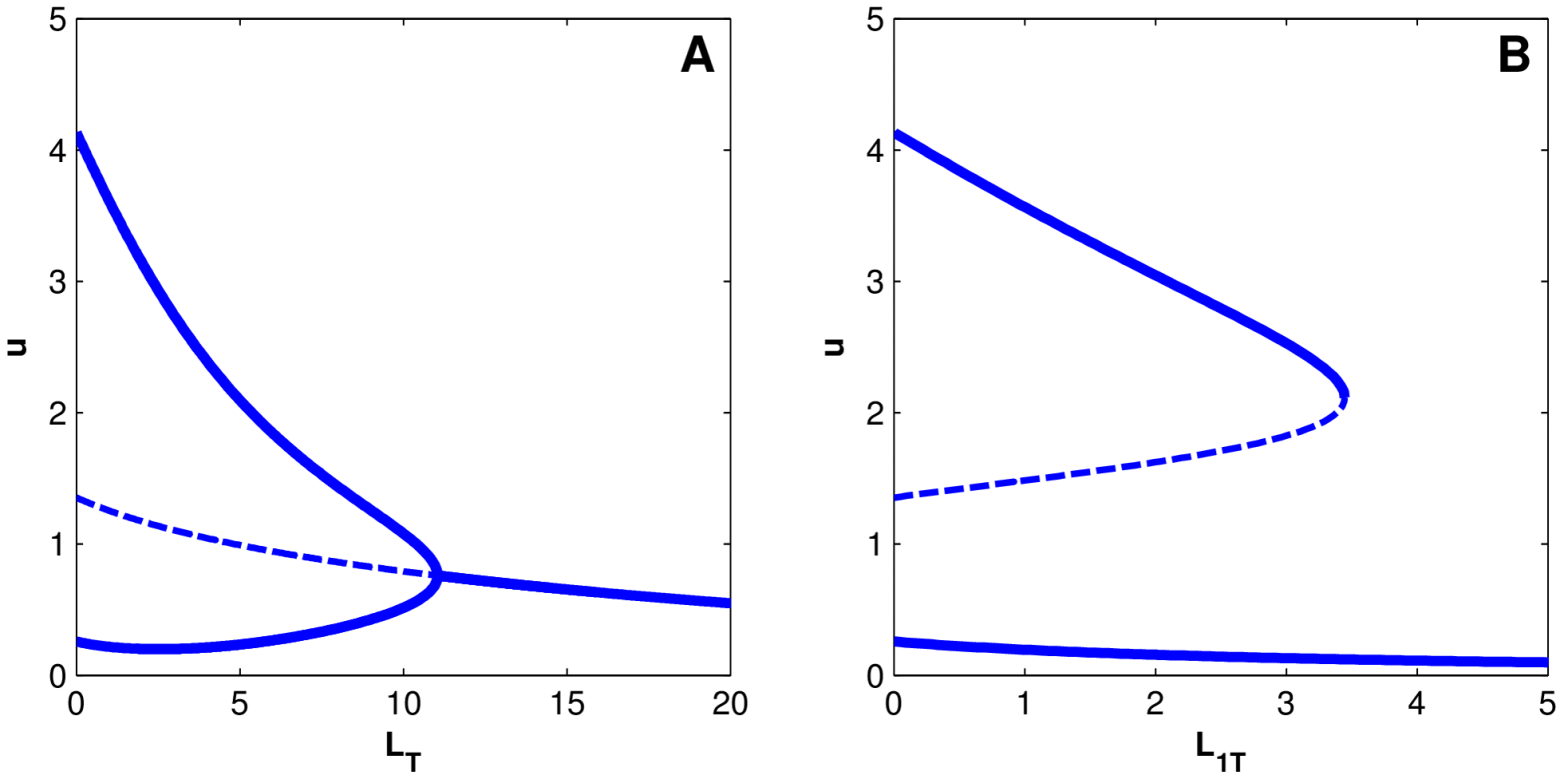
Bifurcation diagram of the genetic toggle switch when the repressor can decay from the load-repressor complex. The thick lines are stable steady states, the dashed lines are unstable steady states. (A). A load is added symmetrically to both sides of the toggle. The stable states of only one Repressor molecule with respect to the load are shown. With zero load the toggle switch is bistable with well separated steady states. As the load increases, the two stable states approach each other and the unstable state, and eventually merge in a bifurcation at a critical value of the load. The system is monostable beyond this critical value. (B) A load is added only to Repressor 1. The high state of Repressor 1 approaches the unstable steady state as the load increases and merges with it at a critical value of the load, leaving only the lower state accessible to the system.

The exception is when the repressor molecule can degrade even when bound to the load, which may be relevant in some experimental situations. As Fig. 2A shows, when a load is added symmetrically to both sides of the toggle switch, the two stable states approach each other and eventually annihilate, leaving a monostable system. Fig. 2B shows that when a load is added only to one side, the system again goes from bistable to monostable at some critical value of the load. In effect, the upper stable point vanishes and is no longer accessible due to leakage of the repressor affected by the load.

The reason for the change in steady state behavior is made clear on examining the equations of the system. Here we need to incorporate additional reactions that represent the decay of the repressor-load complex into the load alone. This leads to an additional term in the equation for the load and the repressor-load complex (Eq. S44 and S45). However this term does not appear in the equation for the repressors, which continue to be governed by Eq. 1 and Eq. 2. As a consequence in the steady state, the additional terms in Eq. 1 and 2 no longer equal zero and the steady state properties of the switch are influenced by the presence of the load.

As can be seen from an examination of the chemical reaction system, this mechanism of abrogation of bistability arises whenever the load-repressor complex participates in a non-reversible (from the repressor's point of view) chemical process that leads to an unbalanced leakage of the repressor from its function as a repressor by the presence of the load. A more interesting example of such a process could be provided by a chemical reaction system where the load is an enzyme for one of the repressor molecules, which is transformed by the enzymatic action into a protein no longer capable of repression. The mathematical analysis of this case is exactly the same as the model we are currently discussing hence we do not consider it separately here.

However a load can significantly change the dynamic response of the basic genetic toggle switch as we shall see below. We examined two different measures of dynamic response, response time for state switching and the amount of inducer required for state switching.

### The response time for state switching of the toggle switch increases

We measured two response times, the rise time which quantifies the time taken for the concentration of Repressor 2 to increase from its low or zero level in state 1 to its high level in state 2, and the decay time which measures the time taken for Repressor 1 to decay from its high level in state 1 to its low level in state 2, in both cases in response to a constant inducer. Specifically the rise time measured the time to go from 10% to 90% of the steady state maximum, while the decay time measured the time to go from 90% to 10% of the steady state maximum. These measurements were made using the deterministic model in the cases when the load was applied only to one side and to both sides of the switch.

**Figure 3:**
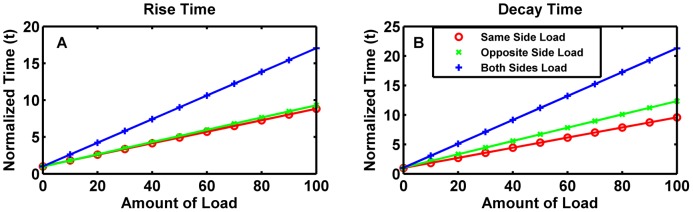
Effects of a load on transition times of the basic toggle switch. (A). The time taken to reach 90% of maximum value for the protein undergoing a low-to-high transition as a function of the Load, normalized by the steady-state amount of Repressor 1. Normalized time is a unit-less number defined by the transition time (rise or decay) of the system at a given loading condition divided by the transition time (rise or decay) of an unloaded system. (B). The time taken for the concentration of the protein undergoing a high-to-low transition to reach 10% of its maximum value. The x- and y- axes are the same as for the previous panel.

We found that both the rise time and the decay time increase with increasing load concentration. Interestingly, this relationship was approximately linear in all cases (Fig. 3A & B). The slope of the linear relationship represents the increase in response time due to unit increase in load. We found that the slope of the line was larger when the load was applied to the opposite side of the system before the switching rather than the same side (Fig. 3A), indicating that it is harder to switch out of a state without a load to a state with a load than the reverse. However when a load was applied to both sides, the slope of the linear fit was higher than when the load was only on the opposite side, suggesting that both the “opposite side” and the “same side” delays are operating.

While we also found an approximately linear relationship between the decay time and the concentration of the load, there was little difference between the decay times for the state with the load (“same side load”) and the state without a load (“opposite side load”) at our base parameter values. Thus the load affects rise time and decay time differently. When a load was applied to both sides of the switch, the slope of the decay time linear fit was larger, again indicating the operation of both delays.

We tested these results by changing parameter values for the binding of the load (Table S1) and found that in all cases we obtain a good linear fit for the response time. For the rise time, the slope was uniformly larger when the load was applied to the opposite side as compared to the same side, and it was the largest when loads were applied on both sides. For the decay time, the slope could be larger or smaller when the load was applied to the opposite side of the decaying state compared with the same side, but it was always larger than both when a load was applied on both sides. The slope depended non-monotonically upon the dissociation constant (Kd) of the binding between the repressor protein and the load, with both low Kd and high Kd having a smaller effect that those in between (Fig. S1). This was because when the Kd was low, i.e. strong binding, the concentration of the load-repressor complex was unaffected by the state of the switch. However when the Kd was high, the maximum concentration of the load-repressor complex was smaller, thereby having a lesser effect on the system (Fig. S2). Thus response times are maximized when the load acts as a dynamic sink, i.e. it takes up newly synthesized repressor when the state changes from the unloaded to the loaded side, and releases the bound repressor when switching from the loaded side to the unloaded side.

Previous studies of response times of biochemical networks with and without a load have also seen monotonic increases in the response time of simple transcriptional circuits [37]. However the extremely consistent approximately linear response we see under wide variation in parameter values is extremely intriguing.

An increase in response time should also imply that the concentration of inducer required to shift states should also be affected, especially when it can decay. In accordance with this expectation we also found that the concentration of inducer required to switch states increased exponentially with increasing load, as seen in Table S2. The parameter of the exponential fit was dependent on the inducer decay rate, indicating that the amount of time the inducer remains above a threshold is the key factor governing the switching. We find that this response to a load is unaffected by the mode of switching the toggle, and induction by repression of the current state yields the same qualitative results (Table S2 & S3).

In our analysis so far we have assumed that the total concentration of the load is fixed. We now analyze the case when the load is generated by a constitutively active promoter and can decay at a first order rate. We find that in this case too the qualitative features of the transition time remain the same as the toggle switch with a fixed load, i.e. it was approximately linear in all parameter regimes tested (Supplementary Text S1 section 1.5, Fig. S3 and Table S4). The reason why we do not see a difference from the basic toggle switch is that the transition times ultimately measures time between steady states, and we wait for the system to come quite close to the steady state value (90%). Thus the concentration of the load has also reached a steady state value and the system behaves as it would with a fixed load.

**Figure 4:**
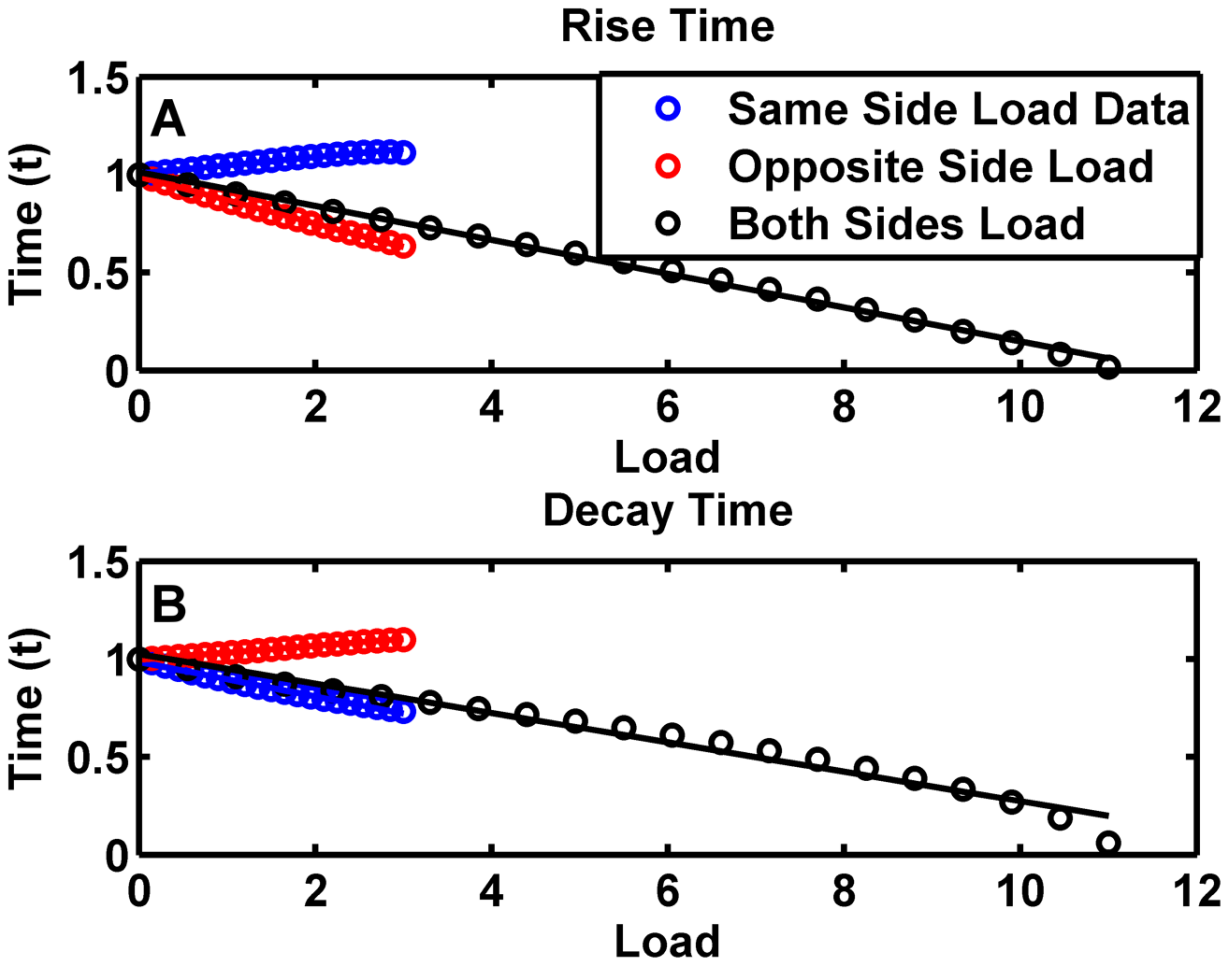
Effects of a load on transition times of a toggle switch without the protection assumption. (A). The time taken to reach 90% of maximum value for the protein undergoing a low-to-high transition as a function of the load. The system is de-dimensionalized as described in Supplementary Text S1 section 1.1 and 3.2.1. (B). The time taken for the concentration of the protein undergoing a high-to-low transition to reach 10% of its maximum value. Note that the linear relationship for both-sided load transition times, and same-sided decay time, and opposite-sided rise time has a negative slope. The relationship for same-sided rise time and opposite sided decay time has a very small, but positive slope.

We also tested the response times when the repressor can leak away from the systems after binding with the load. Here we find that (Fig. 4) when a load is applied to the same side, the rise time continues to increase monotonically linearly with the load but the decay times decreases monotonically with the load. However when a load is applied to both sides, we find a negative linear relation between the transition times for both rise and decay and the load.

The reasons for the change in behavior is because as we saw previously, when the repressor can leak away from the repressor-load complex, a load has a dramatic effect on the bistability properties of the switch, abrogating bistability very quickly (Fig. 1). When only one repressor has a load, the high state of that repressor approaches the unstable state, indicating a decrease in the domain of attraction. Shifting out of that state thus becomes easier with increasing load. When both sides have loads, both stable states approach the unstable state, therefore shifting out of either state becomes easier, and both transition times decrease.

### Dramatic changes in the potential energy landscape and probability distributions of the toggle switch

The modulation in the dynamic properties of the basic genetic toggle switch discussed above suggests that the load has altered the potential energy landscape of the toggle switch, making it harder to switch. For two-dimensional and higher systems, such as the toggle switch, analytical methods to construct the potential landscape are not available, but a quasi-potential can be constructed from the probability distribution function of the concentrations of the repressor molecules, where the quasi-potential is given by the negative of the natural logarithm of the probability distribution [43], [44]. To calculate this we performed Monte Carlo simulations of the toggle switch using a Gillespie type algorithm elaborated in the Methods section. When the toggle switch is symmetrically balanced, both the probability distribution function and the potential energy landscape are completely symmetric. If the system is started in State 1, random fluctuations can drive it into State 2 and vice versa. The probability distribution can then be constructed by counting the frequencies of these random fluctuations. However since the genetic toggle switch can be very stable, a numerical computation of the potential energy landscape requires impractically long simulation times (as we show below). While computational methods to sample rare trajectories in such cases exist, they are very sensitive to choices of parameters [42], [45]. We developed a computational protocol in order to numerically obtain the probability distribution function of both protein concentrations and the transition times. We chose an appropriate volume for the genetic toggle switch such that exactly the same parameters as in the deterministic simulations led to the operation of the toggle switch with only a small number of proteins. The toggle remains bistable in this regime but the small protein numbers vastly increases spontaneous stochastic fluctuations arising out of multiplicative noise in the system and allows the simulation to explore parameter space and collect enough data.

**Figure 5:**
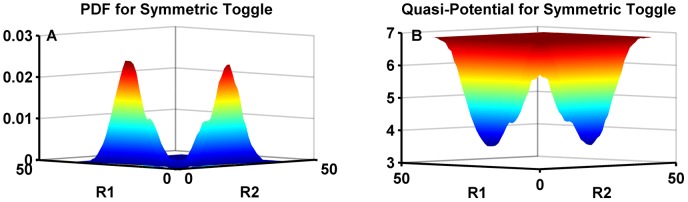
The probability distribution function and the quasi-potential of the genetic toggle switch without a load. (A). The probability distribution function of a toggle switch without a load. The x- and y- axes here represent the number of molecules of Repressor 1 and Repressor 2 respectively, while the z-axis is the frequency of its occurrence. Note that the distribution is symmetric as expected. (B). The quasi-potential of the symmetric toggle switch, showing the symmetric double-well potential constructed by taking the negative logarithm of the probabilities shown in (A). A small offset of 0.001 was added to the probabilities to prevent taking the logarithm of zero. This does not change the shape of the well.

Our simulations showed that the switch switched states a large number of times. In order to account for differences in the time step in different states, the probability density function of the concentrations was constructed using a time trace collected after approximately 1 second intervals. As Fig. 5 shows, for a symmetric switch we obtain a symmetric bi-modal probability distribution that corresponds to a double-well potential.

**Figure 6:**
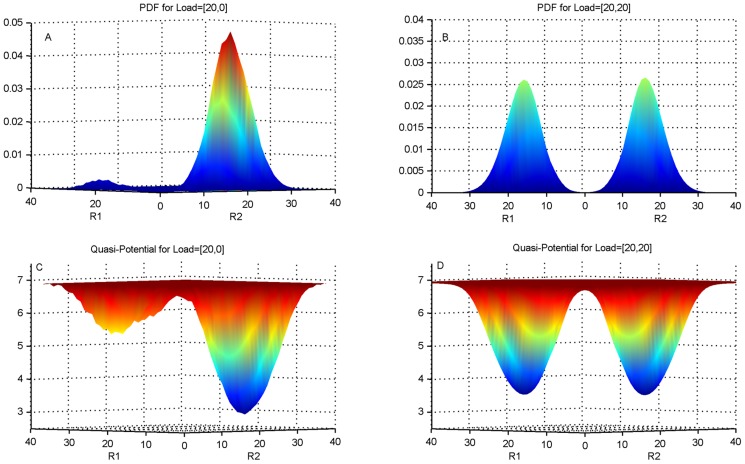
The probability distribution function and quasi-potential of a toggle switch with a load. The 3-dimensional plot is viewed with the xy-plane horizontal for better contrast. The x- and y-axes are numbers of molecules of R1 and R2 while the z-axis is either probabilities or the quasi-potential. (A). The probability distribution function (pdf) of the toggle switch of Fig. 5 but now with a load of 20 molecules on Repressor 1 (R1). (B). The pdf of the toggle switch with a load of 20 molecules on R1 and 20 molecules on R2. (C). The quasi-potential of the toggle with a load of 20 molecules on R1, i.e. corresponding to panel A. (D). The quasi-potential of the toggle with equal loads of 20 molecules on each repressor, i.e. corresponding to panel B.

When we add a load to the system asymmetrically, in the form of a binding partner for the Repressor 1, we find that the probability distribution becomes extremely skewed, and the total weight of the probability distribution corresponding to the other side, i.e. Repressor 2, dramatically increases (Fig. 6A). This indicates that the underlying double well potential has become skewed and the state 2, corresponding to high Repressor 2, has increased its stability at the cost of State 1 (Fig. 6C). When a load is applied to both sides symmetrically, the concentration probability distribution reverts to a symmetric bimodal distribution corresponding to a symmetric double-well potential (Fig. 6B & D).

**Figure 7:**
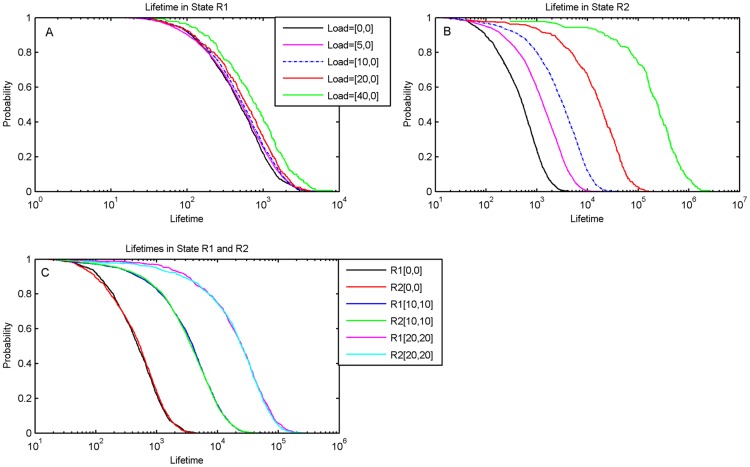
Distribution of the lifetimes of the toggle switch with and without loads. The time the system spent in either state R1 or state R2 was calculated from the time trace of the stochastic simulations and a histogram made of the results. The histogram is shown on a semi-log plot to accommodate the data on a single chart. (A). Lifetimes in State R1. The unloaded state is the solid curve that is to the extreme left of the others, showing that the lifetimes in state R1 increase slightly on addition of load on R1 alone due to the “same side effect”. (B) Lifetimes in State R2 when load is on R1. The solid curve on the extreme left is the unloaded state. There is a significant increase in lifetimes due to the “opposite-side effect” of the load on R1. (C). Lifetimes with a balanced load, showing that both the states R1 and R2 get stabilized with a significant increase in lifetimes on addition of a small load on both sides. Note that the distributions for R1 and R2 for equivalent cases coincide as should be expected.

In order to test this directly we calculated the distribution of lifetimes in state 1 and the lifetimes in state 2. As shown in Fig. 7, when the switch is symmetric with no load, the lifetime distribution is exponential, as should be expected for a simple two-state system. However when the load is applied to Repressor 1, the probability distribution of the lifetime in state 2 increases dramatically. The average lifetime of state 1 also increases but only by a very small amount. The time spent in state 2 does not appear to saturate, and continues to increase with increasing load. When loads are applied symmetrically to both sides, the lifetime histogram in Fig. 7 indicates that both sides have been stabilized since the system spends significantly longer time in each state. Note that in an equilibrium system this would have been indicated by the deepening of the potential well. However in non-equilibrium systems the potential well picture does not completely capture the dynamics and there is an additional contribution from a “curl flux” [43], [46] that needs to be taken into account. For our purposes calculating both the distribution of concentrations and the distributions of lifetimes captures the dynamics of the toggle switch.

To test whether our results change for higher protein concentrations, we increased protein concentrations about fivefold and recalculated the probability distribution function. We find that our qualitative results remain robust despite the increase in protein concentrations (Supplementary Text S1 section 1.3 and Fig. S4). Switching between states is rare at these protein numbers, with a mean residence time in state R1 for the unloaded switch being approximately 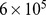 min against about 700 min for the basal case considered, a difference of almost three orders of magnitude. However as for the basal case, the quasi-potential landscape skews significantly with the addition of a load on the switch.

### “Opposite Side effect” dominates the load effect in the basic toggle switch

These results allow us to interpret the dynamic results that we obtained earlier. If the system is in state 2 and there is a load on state 1, a transition requires an increase in Repressor 1 concentration in order to suppress the production of Repressor 2. A load on Repressor 1 however competes with the promoter of Repressor 2 for binding with Repressor 1, and thereby reduces the effective concentration of Repressor 1. This effectively stabilizes state 2. The dynamic analysis shows that state 1 not only remains an attractor state but in fact it takes a longer time, and more inducer, to shift out of state 1 as compared with the no-load situation. This is because the load also acts as a reservoir for Repressor 1, and in fact increases its total concentration. This slows down the transition to state 2. Interestingly this “same side effect” is generally weaker than the “opposite side effect” above. In agreement with this picture, the stochastic simulations show that the distributions of lifetimes in state 1 broaden slightly on addition of a load.

If the load is present symmetrically on both sides, the concentration histograms in Fig. 6 and the time histograms in Fig. 7 indicate that both states have been stabilized, due to a combination of the ‘same side’ and the ‘opposite side’ effect now acting together to stabilize each state of the switch. In the dynamical simulations this is seen by the increased slope of the response time line for the case of a load on both sides. Results for additional parameter values are shown in Fig S15 and Fig S16.

### Positive feedback moiety makes toggle switch tunable

**Figure 8:**
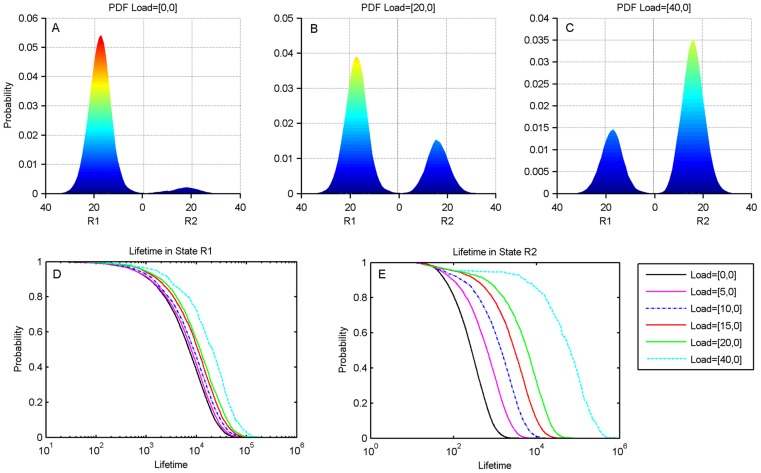
The genetic toggle switch with a positive feedback motif on Repressor 1 (R1). (A). The probability distribution function (pdf) with no load. The positive feedback on Repressor 1 leads to a pdf skewed in favor of R1. (B). The pdf with a load of 20 molecules on R1 showing the increase in the weight of R2 due to the “opposite side effect”. (C). The pdf with a load of 40 molecules on R1. This load is more than enough to skew the pdf in favor of state R2. (D). Histogram of lifetimes in R1 with varying levels of load on R1. Comparison with panel A shows that the unloaded state has been stabilized by the positive feedback. Note that the lifetimes increase very slightly due to the “same side effect”. (E). Histogram of lifetimes in R2 with varying levels of load on R1. The unloaded case is the curve on the extreme left. Note the initial asymmetry in the lifetime distribution due to the positive feedback, as well as the large increase in lifetimes with the inclusion of a load.

When a positive feedback moiety is introduced in the toggle switch, we again see a linear relationship between the rise time and the decay time of the two states of the switch and the load (Fig. S5). Therefore here too the load appears to be skewing the underlying potential landscape of the switch. Using stochastic simulations we constructed the probability distribution function of this toggle switch as described above. We found that even in the absence of a load, when a positive feedback moiety is introduced on one side of a toggle switch, the probability distribution for the toggle switch, and hence the quasi-potential landscape, becomes extremely skewed in favor of the state with positive feedback as shown in Fig. 8A. Even with no load on the system, the switch is biased to State 1 and the lifetime spent in State 1 is much longer than in State 2. If a load is added to R2, the opposite side effect additionally favors State 1. If a load is added to R1 however, the opposite side effect favors State 2 (Fig. 8B). It is possible to balance these effects resulting in a more even distribution by adjusting the load on R1 and the strength of positive feedback. As the load on R1 is increased beyond this balance point, the opposite side effect dominates and the probability distribution becomes skewed toward State 2 (Fig. 8C). As the opposite side effect increases with increasing load, the lifetime in State 2 also increases in agreement with the findings for the regular toggle switch (Fig. 8D). The lifetime in State 1 also increases by a smaller amount, as for the regular toggle switch (Fig. 8E).

For the toggle switch with the positive feedback moiety, we can also check the consequences of allowing repressor leakage through the repressor-load complex. As shown in Fig. S13, this addition to the system affects the steady state properties of the switch and bistability is abrogated after the load increases beyond a critical value, when load is present for both sides or only one side.

### Loads fundamentally transform positive feedback based switches in signal transduction

**Figure 9:**
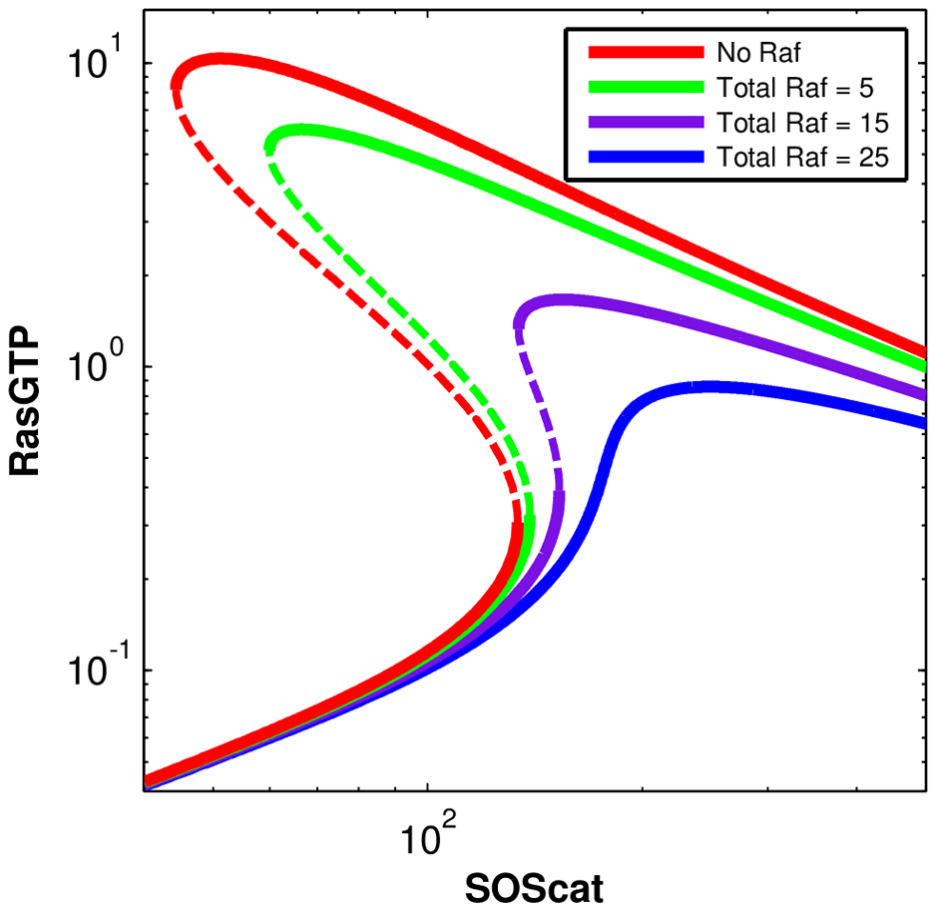
Bifurcation diagram of the Ras switch with different levels of Raf (load) on the system. The total number of SOS in the simulation box is used as the parameter being tuned, which varies from 0 to 1000. For Raf = 0, Raf = 10 and Raf = 30, there are two bifurcations as SOS is increased. In the first bifurcation a new high valued stable steady state appears along with the low valued stable steady state. In the second bifurcation, the low valued stable state disappears leaving behind only the high valued state. The dotted line marks the unstable steady state that also comes into existence in the bistable region. As total Raf increases, the two bifurcations approach each other. When Raf = 50, the system has lost both of its bifurcations and is characterized by a single stable steady state at all values of Raf.

The RasGTP system shows a bistable transition from a low RasGTP state to a high RasGTP state as the activating signal, in our case the number of SOS molecules, are varied. As Fig. 9 shows, a system with no Raf shows a classic Z-shaped bifurcation diagram with two bifurcations as SOS is varied. The first bifurcation marks the transition from a monostable low-RasGTP state to a bistable system with a “high” RasGTP state (and an unstable intermediate state). The second bifurcation marks the transition from the bistable state to another monostable state with a high concentration of RasGTP.

When Raf is added to the system, the bifurcation diagram changes and the two bifurcations start approaching each other. This is because the effect of adding Raf is equivalent to sequestering away some of the activated RasGTP in an “inactive” complex. When Raf concentration crosses a threshold, the bifurcations annihilate each other and disappear. This system is now characterized by a single stable point for all concentrations of SOS, and the disappearance of the threshold for Ras activation. While there appears very little free Ras, in reality, even for low SOS concentrations there is a large concentration of the activated RasGTP-Raf complex (since RasGTP in these complexes is also protected from the action of the Ras GTPases).

This can be seen in another way in Fig. S8 where the stable state of RasGTP is plotted against the level of total Raf in the system, keeping the level of SOS constant. Again we see that a bistable system is transformed into a monostable system when Raf increases beyond a threshold. These results are exactly the same for the model which assumes Michaelis-Menten kinetics, except for small changes in molecule numbers, as can be seen in Fig. S7 and S9. Results do not change on changing load-binding parameters (Fig. S10, S11)

Thus the addition of the Raf scaffold, which is an integral part of the MAPK cascade, fundamentally changes the qualitative behavior of the positive feedback switch. The main reason why the steady state bifurcation properties are affected here in contrast to the basic genetic toggle switch is that for this signaling circuit, as seen in Eq. 22–25, total Raf and Ras are conserved, as is typical for a short timescale signal transduction system. These conservation laws couple Raf concentration to RasGTP concentration even at steady state. Therefore adding Raf to the system effectively reduces total Ras concentration since Raf sequesters away Ras from the switch.

To see this more generally, consider for example a chemical reaction system comprising of n-species 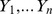. Let us assume without loss of generality that the species 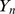 is coupled to a downstream circuit through a binding reaction with a load, 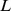. The (n+2) differential equations describing this system are:
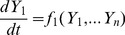
(26)

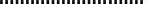


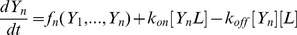
(27)

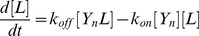
(28)

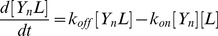
(29)


Note that for simplicity of notation we have not indicated the dependence of the dynamical system on its own parameter values. Now in the steady state, if the set of equations is complete, the left side uniformly goes to zero and we recover the result that the steady state remains exactly the same with or without a load, as for the genetic toggle switch. However let us now assume that we have an additional conservation law, say,
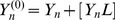
(30)


This conservation law implies that one equation in our dynamical system is redundant, and we need to drop one equation to make the system linearly independent. We can decide to drop Eq. 19, and substitute 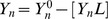 in Eq. 20 and Eq. 21 and solve the resulting (n+1) equations for the (n+1) unknowns, 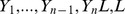, obtaining 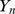 as a residual from Eq. 22. Thus the steady state solutions of the 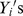 now involve the amount of the load. Clearly, the existence of the conservation law has led to a change in the steady state properties of the dynamical system. Note that 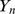 itself would usually enter (by itself or in the form of other complexes, which then would also need to be accounted for in the conservation law Eq. 22) into one or more of the equations for the remaining species, 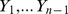. This would result in the equations for those other species explicitly involving, and thus depending upon the level of the load. For the Ras system above, Eq. 16 couples the load, Raf, to the concentration of Ras. However Ras concentration and SOS concentration are also coupled. Thus the load explicitly affects the steady state values of all species concentrations in this system. This leads to a fundamental qualitative change in the bifurcation properties of the system.

## Discussion

It has been pointed out previously that significant sequestration effects can abrogate zero order ultrasensitivity [26], [47], [48], can change the dynamics of simple phosphorylation circuits [23], [24] and change oscillatory behavior in some circuits [27]. We add to this body of work by demonstrating that the addition of a simple binding partner to the output protein of a genetic or signaling switch can have dramatic effects on its properties, and can fundamentally change the operation of the switch.

For a genetic toggle switch with two mutually repressing proteins such as the classic switch built by Gardner et al. [5] we showed that even though the presence of the binding partner does not alter steady state properties of the switch, it can drastically change the dynamic properties. Using a novel potential landscape analysis, we showed that this is because the addition of the binding partner skews the underlying quasi-potential, making one state significantly more stable than the other. In practice therefore, a genetic toggle switch that is significantly skewed towards one side may never properly function as a switch. Thus the downstream consequences of such loads need to be taken into account when designing larger synthetic circuits with the toggle switch as one of the elements.

On the other hand this phenomenon actually provides a way of making artificial switches tunable. It is possible to engineer a biased switch merely by adding a load on the opposite side of the toggle, which is a useful device when engineering a switch that is designed to be switched on only in special circumstances. A load on both repressor proteins similarly stabilizes both sides of the toggle switch. This could be useful when working with synthetic components with low concentrations in cells, especially those that display stochastic switching. A load on both repressor proteins can significantly increase the stability of such a toggle.

In natural systems, mutually repressing toggle switches are often found with other complexities, such as a positive feedback motif on one side. The positive feedback motif by itself biases the toggle switch by stabilizing the side it is on at the expense of the other side. A load on the same side then stabilizes the opposite side, and can re-establish balance between the two quasi-potential wells. For engineering circuits in multi-cellular organisms, it is worth noting that that feedback between the load on a toggle switch and the strength of the positive feedback may ensure that the switch operates efficiently even in the presence of cell to cell variability in the load. How loads vary between cells and in multi-cellular organisms is an interesting question to explore in future work. The presence of the positive feedback provides a potential target for evolutionary fine-tuning of the switch.

In the above analyses we use novel potential landscape methods that have proved useful and insightful in fields such as protein folding to discuss the fundamental properties of a dynamical system that shows not apparent changes in its stability properties. We demonstrate that these methods, though still relatively underdeveloped for use with non-equilibrium chemical reaction systems, hold promise for understanding the dynamics of such systems beyond what linear stability analysis can provide. However there are certain conditions when addition of a load changes the stability properties of the genetic toggle switch. One class of such effects happen when the repressor can leak away from the repressor-load complex, as can happen either when the repressor can decay or degrade when bound to the load, or when the load can modify the repressor and make it unable to repress. We show, employing standard bifurcation analysis, that additional loads in this system can abrogate the switch-like properties of the toggle switch entirely.

In switches based on autocatalysis or positive feedback with an enzymatic deactivation, such as is often found in signaling systems, the effects of a load are equally dramatic. We show that in a simple model of Ras activation, adding a small concentration of Raf molecules changes the bifurcation diagram of the signaling circuit and can completely abrogate the bistability in the system. While we have chosen a specific example of Ras activation, our simplified model, with an autocatalytic forward reaction and an enzymatic backward reaction is a minimal model for a many positive feedback switches. The change in the bifurcation diagram arises from the conservation laws that couple the concentration of the load with the concentrations of the proteins in the upstream module. Given the sensitivity of non-linear dynamical systems to initial conditions, it should probably be expected that many, if not all, positive feedback based switches that operate at the short timescales of signal transduction, and therefore must possess these conservation laws, should exhibit this sensitivity to the effect of a load.

Our results throw up an interesting puzzle for quantitative biologists. In many natural signal transduction systems such as the MAPK cascade, the concentration of the output of a bistable switch is quite comparable to the concentration of the load, thus significant changes in load concentrations could have dramatic effects on the behavior of the switch. However it has also been shown that there is a significant cell to cell variability in protein concentrations [49]. How do cells ensure that positive feedback based switches such as the Ras switch continue to operate robustly in the bistable regime? Additional regulatory mechanisms involving feedback between the load and its partner protein may exist that confer robustness to the qualitative behavior of the biochemical switch. Arguably some of the bells and whistles of natural protein networks that are often disregarded when analyzing the network may in fact be performing this role. In other words, self-assembled switches have to be complex! In this context it is worth mentioning that it has been persuasively argued [50], [51] that some biological circuits maintain robustness of “fold-change’ behavior rather than absolute levels of protein concentration. It is possible that additional protein-protein interactions that couple concentrations of loads with output proteins may end up in performing this function. Another significant factor that needs consideration is the role of spatial segregation in producing feedback from the downstream module to the upstream one. In fact it has been shown experimentally that MAPK substrates sequester activated MAPK in the nucleus, and thus protect it from cytoplasmic phosphatases. Changing the concentration of one substrate therefore affects the concentration of activated MAPK [52].

Previous discussions of the effect of loads on the operation of circuits have suggested the use of insulators, that is circuit elements that insulate the upstream module from the downstream module [22]. The initial suggestions for building insulators in Ref. [22] involved incorporating signal amplification along with negative feedback in the upstream circuit. Another way of insulating the circuit is to ensure that the demand of the load for its cognate repressor is never significant compared to the total amount of repressor. For a genetic switch therefore, a possible insulating mechanism is if the link to the downstream circuit is through a promoter. For example, consider making an AND gate from an output of the toggle switch. This can be done by inserting a constitutively produced protein Y that binds to R1 such that the complex is a transcription factor for another protein, say Z. Thus there is an AND relationship between the two inputs, Y and R1 and the output Z. To offset the effect of load induced modulation of the dynamics of R1, an additional step can be inserted such that R1 first binds to the promoter region of another gene that codes for protein X and activates its transcription, and it is the protein X, rather than R1, that can bind to Y and activate production of Z. The advantage of adding this extra step is that the concentration of the promoter for X is very small compared to the concentration of R1, and therefore load induced modulation of the upstream toggle can be kept at a minimum. Note however that this cannot be done without the additional cost of the time delay required for the transcription and translation of X.

As can be seen, any additional step or series of steps that can amplify a weak signal can act as an insulator. Another standard example of an amplifying circuit is a phosphorylation cascade which is especially relevant when considering Ras activation since it directly leads to the MAPK phosphorylation cascade. Phosphorylation cascades are also very fast, and therefore do not face the additional time delays of an additional transcriptional step. From the point of view of synthetic circuit design, the insulating mechanism here could be constructed by designing a weak binding affinity of Ras (or the synthetic protein that plays that role) for Raf (or the equivalent protein). The bound complex then catalyzes a phosphorylation cascade that ends by connecting to the downstream circuit.

Note that this method of insulation does not have the same time delay costs as the additional transcription steps. However it does come with the metabolic costs of having to produce large amounts of proteins that are essentially serving no useful physiological purpose for the cell. This cost could be relevant in some synthetic biology applications, and certainly needs to be evaluated during circuit design. It has been shown in the context of phosphorylation cycles that insulation always carries a metabolic cost, and in general better insulation carries a greater metabolic cost [53].

The existence of the MAPK phosphorylation cascade however begs the question whether it serves the purpose of insulation of the upstream Ras circuit from the downstream circuit. While it is not possible to answer this intriguing question without further experiments, it does appear that the Ras-Raf complex is present is quite large numbers on activated cells. This would suggest that insulation is not the function for which the cascade may have evolved. Our own analysis of the genetic toggle switch with the positive feedback motif suggests that Nature may prefer more complicated forms of regulation that balance the different components of the circuit. However there is no reason why both methods cannot be utilized. To our mind this is a very exciting question that requires more attention from experimentalists and theorists alike.

It should also be noted that due to non-specific binding of transcription factors with DNA as well as between proteins, every circuit in the cell, real or synthetic, operates in the presence of a load. Variability in the functioning of circuits that are seen when transferring synthetic circuits between species, or even in different cells, may be a result of not only differences in basic protein concentrations, but also of this undervalued but nevertheless tangible load. Based on this reasoning we predict that some of the host-dependent effects that complicate synthetic biology, i.e. a synthetic circuit that works in one organism not performing well in another, are in fact due to changes in the intrinsic load due to non-specific binding when changing hosts.

Our analysis underscores the importance of incorporating loads when simulating models of switches in natural and synthetic systems. Mathematical analysis of switch-like motifs therefore would do well to at least include a load on their output proteins, in order to incorporate the possible effects of load induced modulation on the circuit.

## Supporting Information

Figure S1 **Surface plots showing response times of the simple genetic toggle switch with changes in load (L) and changes in the dissociation constant (Kd) of binding with load.** The units of L and Kd are (molecules/µm^3^). The z-axis measures the response time indicated in the title. “Same Side Rise” and “Same Side Decay” refers to the rise time and decay time when the load is on the same side as the repressor whose concentration is increasing. “Opposite Side Rise” and “Opposite Side Decay” refers to the rise time and the decay time when the load is on the other side of the repressor whose concentration is increasing. “Both Sides Rise” or decay refer to the rise and decay times when a load is present on both sides (symmetrically). The plot shows that at every Kd, the relation between the response time and load is approximately linear. The response time is largest for the case of “Both Sides Rise” followed by “Opposite Side Rise”. The response time is also non-monotonic with respect to the Kd for a given load, and is maximized at intermediate values of Kd.
(TIF)

Figure S2 **Time plot of switching of the simple toggle switch with a load on Repressor 1, at three different values of the dissociation constant.** In all three cases the system is switched by providing 150 molecules/µm^3^ of an inducer at 1000 minutes. The inducer stays constant at that value and is not shown in the plots. The left panel has a very high dissociation constant (Kd = 1000 molecules/µm^3^) of binding between the load and the repressor, due to which the load has a minimal effect on the system. The middle panel has an intermediate value (Kd = 1 molecules/µm^3^) because of which the load acts as a dynamic sink by releasing Repressor 1 and slowing the switching. The right panel shows the effect of a small dissociation constant (Kd = 10^−3^ molecules/µm^3^). At such strong binding affinities, all of the load is always bound to Repressor 1. Thus the load has minimal effect on the switching dynamics. In all cases total load concentration is 100 molecules/µm^3^.
(TIF)

Figure S3 **Effects of a dynamic load on dynamics of a symmetric toggle switch.** (A). The time taken to reach 90% of maximum value for the protein undergoing a low-to-high transition as a function of the equilibrium constant of a dynamic load. Normalized time is a unit-less number defined by the transition time (rise or decay) of the system at a given loading condition divided by the transition time (rise or decay) of an unloaded system. (B). The time taken for the concentration of the protein undergoing a high-to-low transition to reach 10% of its maximum value.
(TIF)

Figure S4 **Stochastic time trace and the probability distribution function of repressor concentrations for the large volume simulations.** (A). Comparison of time traces of the stochastic simulations of the simple toggle switch with basal parameters (top panel) and a larger volume (bottom panel). The average molecule number is about 5 times greater, and the number of transitions are significantly fewer. (B). The probability distribution function of the genetic toggle switch with the larger molecular number without (left) and with (right) a load. The effect of a load on R1 is qualitatively the same for this system as for the smaller system. Since transitions are slower the data are more uneven for this simulation.
(TIF)

Figure S5 **Transition times in a genetic toggle switch with a positive feedback moiety.** In all cases the strength of the positive feedback (denoted here by P instead of *ρ*) is 3.5 on either Repressor 1 (R1) or Repressor 2 (R2). Top Left: Rise time - time to transition INTO state R1 with the positive feedback on R1. Note that the rise time is larger at nonzero loads when the load is on R2 or when the load is on both sides, in agreement with the simple toggle switch. Top Right: Rise time - time to transition INTO state R1 with the positive feedback on R2. Bottom Left: Decay time - time to transition OUT OF state R1 with the positive feedback on R1. Bottom Right: Decay time - time to transition OUT OF state R1 with the positive feedback on R2.
(TIF)

Figure S6 **Probability distribution functions of repressor concentrations for the toggle with a positive feedback moiety.** The left panel shows that when ρ = 0, the switch is balanced evenly. As ρ increases, the side of the switch with the positive feedback becomes more and more prominent, at the expense of the other side. When ρ = 5, the system spends most of its time in one state.
(TIF)

Figure S7 **Bifurcation diagram of the Ras switch with different levels of Raf (load) on the system for the model with Pseudo Steady State Assumption (PSSA).** The total number of SOS in the simulation box is used as the parameter being tuned, which varies from 0 to 1000. For Raf = 0, Raf = 10 and Raf = 30, there are two bifurcations points as SOS is increased. In the first bifurcation a new high valued stable steady state appears along with the low valued stable steady state. In the second bifurcation, the low valued stable state disappears leaving behind only the high valued state. The dotted line marks the unstable steady state that also comes into existence in the bistable region. As total Raf increases, the two bifurcations approach each other. When Raf = 50, the system has lost both of its bifurcations and is characterized by a single stable steady state at all values of Raf.
(TIF)

Figure S8 **Bifurcation diagram of the Ras activation model based on Law of Mass Action (LMA).** Here the total number of Raf molecules (Raf_T_) is the primary parameter being varied. Without Raf, the Ras activation system is bistable as reported. With increasing Raf_T_, the “high” stable steady state branch comes closer with the unstable steady state branch and both are eliminated after a threshold of Raf_T_. A monostable region is maintained beyond the threshold.
(TIF)

Figure S9 **Bifurcation diagram of the Ras activation PSSA model with total number of Raf molecules (Raf_T_) as the primary parameter.** Without Raf, the Ras activation system is bistable as reported. With increasing Raf_T_, the “high” stable steady state branch comes closer with the unstable steady state branch and both are eliminated after a threshold of Raf_T_. A monostable region is maintained beyond the threshold.
(TIF)

Figure S10 **Parameter sensitivity of the bistability of Ras switch to changes in k_off6_.** Increase of k_off6_ results in leftward shifts of both stable fixed points, increase in the bistable regime and increase in maximal RasGTP activation level (Green Line) when compared to baseline with original value (Blue Line). Decrease of k_off6_ (Red Line) results in right shift of both limit points, increase in unstable bistable regime and decrease in maximal RasGTP activation level. Qualitative features of bistability are maintained.
(TIF)

Figure S11 **Parameter sensitivity of the bistability of Ras switch to changes in k_on6_.** Increase of k_on6_ (Green Line) results in right shift of both limit points, increase in unstable bistable regime and decrease in maximal RasGTP activation level when compared to baseline original value (Blue Line). Decrease of k_on6_ (Red Line) results in left shifts of both limit points, increase in bistable regime and increase in maximal RasGTP activation level. Qualitative features of bistability are maintained.
(TIF)

Figure S12 **Comparison between bifurcation diagrams of toy genetic toggle switch with and without protection of repressor degradation when bound with promoters.** If the protection is not included (Blue Line), a minor increase in the bistable region can be observed with right shift of upper limit point and left shift of lower limit point compared to the case with protection assumed (Red Line). Note that this is not the same as degradation after being bound with the load.
(TIF)

Figure S13 **Bifurcation diagram of the genetic toggle switch with positive feedback loop on one side after removal of the protection assumption.** The left panel shows the bifurcation diagram when the load is added symmetrically to both sides. Without load molecule, the toggle switch is bistable as predicted. With the increase in L_T_, the unstable steady state and the “low” stable steady state come closer and meet at certain threshold. The value of “high” stable steady state decreases with increase in L_T_. Beyond the threshold, the toggle switch becomes monostable. The right panel shows the effect of just adding a load to R1. In this case the high state of R1 approaches the unstable steady state, and annihilates itself. The system jumps to the low stable state, which is equivalent to the “high” state of the other repressor.
(TIF)

Figure S14 **Bifurcation diagram of the Ras activation model when Ras can degrade when bound with Raf.** As the number of Raf molecules increase, the bistable region decreases. However unlike the case with no protection, the curve moves to the left. When Raf molecules increase by a large amount, bistability is abrogated.
(TIF)

Figure S15 **Transition times for various k′_on_ and k′_off_ values plotted as a function of load for the basic toggle switch.** Even if the binding-unbinding rates are slower or much faster than protein decay rates, the load-transition time relationship stays linear. A, C, E, G, I, K, M, O, Q and S show the rise time. B, D, F, H, J, L, N, P, R, and T show decay time. (A,B) k′_on_ = 4, k′_off_ = 0.5, Kd = 0.125. (C,D) k′_on_ = 10, k′_off_ = 0.5, Kd = 0.05. (E,F) k′_on_ = 4, k′_off_ = 4, Kd = 1. (G,H) k′_on_ = 10, k′_off_ = 10, Kd = 1. (I,J) k′_on_ = 4, k′_off_ = 20, Kd = 5. (K,L) k′_on_ = 10, k′_off_ = 50, Kd = 5. (M,N) k′_on_ = 4, k′_off_ = 40, Kd = 10. (O,P) k′_on_ = 10, k′_off_ = 100, Kd = 10. (Q,R) k′_on_ = 40, k′_off_ = 400, Kd = 10. (S,T) k′_on_ = 100, k′_off_ = 1000, Kd = 10.
(TIF)

Figure S16 **Probability distributions of repressor concentrations for various values of k′_on_ and k′_off_ for the basic toggle switch.** Even when the binding-unbinding with the load is several times faster than protein decay rates, the basic phenomena discussed in the paper remains unchanged. (A) k′_on_ = 50, k′_off_ = 500 (B) k′_on_ = 500 k′_off_ = 500 (C) k′_on_ = 500 k′_off_ = 5000.
(TIF)

Figure S17 **Bistability of the toggle switch with positive feedback.** A bifurcation diagram of the simple toggle switch with a positive feedback moiety on one side, with respect to the parameter ρ that measures the strength of the positive feedback. Only the concentration of R1 is shown for simplicity. The switch remains bistable till ρ becomes larger than a little over 200.
(TIF)

Table S1 **Slopes of linear fits to rise and decay time with various values of K_off_, K_on_ and β.** The first column reports the values of the dissociation constant (Kd = Koff/Kon) and the kinetic constants of the binding of Repressor 1, 2 or the value for β, which represents promoter strength. The other columns report the slopes of the linear fits of the various rise times and decay times. In all cases the fits have high R-squared values (>0.95). Intercept is 1, as the slopes are normalized to the un-loaded transition time. For K_d_ we change the parameters by two orders of magnitude in both directions to show that the linear relation is robust despite these changes. Note that the relation between rise time or decay time and the binding constant is non-monotonic. Units are as reported in the text.
(DOC)

Table S2 **Exponential Fits of the amount of inducer required to transition states as a function of load.** The basic genetic toggle switch switch was toggled to its other state by production of the other repressor protein by an inducer, given here as a bolus with a decay rate as shown. The size of the bolus was increased until the state changed. This was repeated at different levels of load and the minimum size of the bolus required was fit by an exponential function of the load. The fits are shown here, along with their R-squared values. “Load applied to the opposite side” means switching from a state without a load to a state with a load. “Load applied to the same side” means switching from a state with a load to a state without a load.
(DOC)

Table S3 **Exponential Fits of the amount of inducer required to transition states as a function of load, in the case of induction by repression.** The switch was toggled to its other state by repression of the current state by an external molecule, given to the system as a bolus with a decay rate as shown. The size of the bolus was increased until the state changed. This was repeated at different levels of load and the minimum size of the bolus required was fit by an exponential function of the load. The fits are shown here, along with their R-squared value. Thus the inducer required depends exponentially on the load in both the methods of induction. “Load applied to the opposite side” means switching from a state without a load to a state with a load. “Load applied to the same side” means switching from a state with a load to a state without a load.
(DOC)

Table S4 **Slopes of linear fits to rise and decay time with a dynamic load, with varying values of load decay rate K_d_, load binding rates K_on_ and K_off_, and constant K_1_.** The first four columns report the values of the various parameters. The other columns report the slopes of the linear fits of the various rise times and decay times. In most cases the fits have high R-squared values (>0.95). The two exceptions are >0.90 and starred. Intercept is 1, as the slopes are normalized to the un-loaded transition time. Note that for all cases, the relationship between load (expressed here as K_eq_ = K_b_/K_d_) and transition time is a positive linear relationship.
(DOC)

Table S5 **Rate expressions used for the stochastic simulations of the genetic toggle switch.** The rate expressions used for the stochastic simulation of the toggle switch along with the description of the reaction are listed.
(DOC)

Table S6 **List of reactions in the minimal model of Ras activation.** The reactions in the minimal model of Ras activation, along with the labels of the corresponding rate constants are shown. Parameters used in the simulations are given in Table S7.
(DOCX)

Table S7 **Kinetic rate parameters used for the simulations of the Ras model.** Here the numbers in the subscript of the rate constants in the “Constant” column refer to the reactions shown in the corresponding row of Supplementary Table S6. The meaning of the rate constants are as follows: k_on_ refers to the on-rate, k_off_ is the off rate and k_cat_ is the catalytic rate. The sources for the rates are as shown in the last column.
(DOC)

Table S8 **List of reactions in the toy model of genetic toggle switch.** The reactions in the toy model of the genetic toggle switch, discussed in Supplementary Text S1 section 3.1 are listed. The description of the various chemical species in the reactions are also provided in the Supplementary Text S1.
(DOCX)

Text S1 **Supporting Information including derivation and analysis of toggle switch and Ras models, parameters used for the simulation and their sources, results of the parameter sensitivity analysis, details of the effect of a dynamic load on the genetic toggle switch, results for the Ras model with Michealis-Menton kinetics, results for the models without the protection assumption.**
(PDF)

## References

[1] Wagner GP, Pavlicev M, Cheverud JM (2007) The road to modularity. Nature reviews Genetics 8: 921–931. 10.1038/nrg2267 18007649

[2] Cooling MT, Rouilly V, Misirli G, Lawson J, Yu T, et al (2010) Standard virtual biological parts: a repository of modular modeling components for synthetic biology. Bioinformatics 26: 925–931. 20160009 10.1093/bioinformatics/btq063

[3] Prasad A (2012) Computational Modeling of Signal Transduction Networks: A Pedagogical Exposition. In: Liu X, Betterton M, editors. Computational Modeling of Signaling Networks: Springer. 10.1007/978-1-61779-833-7_10 23361987

[4] Basu S, Mehreja R, Thiberge S, Chen M-T, Weiss R (2004) Spatiotemporal control of gene expression with pulse-generating networks. Proc Natl Acad Sci U S A 101: 6355–6360. 15096621 10.1073/pnas.0307571101 PMC404049

[5] Gardner TS, Cantor CR, Collins JJ (2000) Construction of a genetic toggle switch in Escherichia coli. Nature 403: 339–342. 10659857 10.1038/35002131

[6] Chang DE, Leung S, Atkinson MR, Reifler A, Forger D, et al (2010) Building biological memory by linking positive feedback loops. Proceedings of the National Academy of Sciences of the United States of America 107: 175–180. 20018658 10.1073/pnas.0908314107 PMC2806707

[7] Kramer BP, Viretta AU, Daoud-El-Baba M, Aubel D, Weber W, et al (2004) An engineered epigenetic transgene switch in mammalian cells. Nat Biotechnol 22: 867–870. 15184906 10.1038/nbt980

[8] Ham TS, Lee SK, Keasling JD, Arkin AP (2008) Design and construction of a double inversion recombination switch for heritable sequential genetic memory. PLoS One 3. 10.1371/journal.pone.0002815 PMC2481393 18665232

[9] Anderson JC, Voigt CA, Arkin AP (2007) Environmental signal integration by a modular AND gate. Mol Syst Biol 3: 133–133. 17700541 10.1038/msb4100173 PMC1964800

[10] Tamsir A, Tabor JJ, Voigt CA (2011) Robust multicellular computing using genetically encoded NOR gates and chemical ‘wires’. Nature 469: 212–215. 21150903 10.1038/nature09565 PMC3904220

[11] Elowitz MB, Leibler S (2000) A synthetic oscillatory network of transcriptional regulators. Nature 403: 335–338. 10659856 10.1038/35002125

[12] Atkinson MR, Savageau MA, Myers JT, Ninfa AJ (2003) Development of genetic circuitry exhibiting toggle switch or oscillatory behavior in Escherichia coli. Cell 113: 597–607. 12787501 10.1016/s0092-8674(03)00346-5

[13] Stricker J, Cookson S, Bennett MR, Mather WH, Tsimring LS, et al (2008) A fast, robust and tunable synthetic gene oscillator. Nature 456: 516–519. 18971928 10.1038/nature07389 PMC6791529

[14] Morey KJ, Antunes MS, Albrecht KD, Bowen TA, Troupe JF, et al (2011) Developing a synthetic signal transduction system in plants. Methods Enzymol 497: 581–602. 21601104 10.1016/B978-0-12-385075-1.00025-1

[15] de Lorenzo V (2008) Systems biology approaches to bioremediation. Current opinion in biotechnology 19: 579–589. 19000761 10.1016/j.copbio.2008.10.004

[16] Alper H, Stephanopoulos G (2009) Engineering for biofuels: exploiting innate microbial capacity or importing biosynthetic potential? Nat Rev Microbiol 7: 715–723. 19756010 10.1038/nrmicro2186

[17] Lu TK, Collins JJ (2007) Dispersing biofilms with engineered enzymatic bacteriophage. Proc Natl Acad Sci U S A 104: 11197–11202. 17592147 10.1073/pnas.0704624104 PMC1899193

[18] Anderson JC, Clarke EJ, Arkin AP, Voigt CA (2006) Environmentally controlled invasion of cancer cells by engineered bacteria. J Mol Biol 355: 619–627. 16330045 10.1016/j.jmb.2005.10.076

[19] Lu TK, Collins JJ (2009) Engineered bacteriophage targeting gene networks as adjuvants for antibiotic therapy. Proc Natl Acad Sci U S A 106: 4629–4634. 19255432 10.1073/pnas.0800442106 PMC2649960

[20] Ro D-K, Paradise EM, Ouellet M, Fisher KJ, Newman KL, et al (2006) Production of the antimalarial drug precursor artemisinic acid in engineered yeast. Nature 440: 940–943. 16612385 10.1038/nature04640

[21] Purnick PE, Weiss R (2009) The second wave of synthetic biology: from modules to systems. Nature reviews Molecular cell biology 10: 410–422. 19461664 10.1038/nrm2698

[22] Del Vecchio D, Ninfa AJ, Sontag ED (2008) Modular cell biology: retroactivity and insulation. Mol Syst Biol 4: 161. 18277378 10.1038/msb4100204 PMC2267736

[23] Ventura AC, Jiang P, Van Wassenhove L, Del Vecchio D, Merajver SD, et al (2010) Signaling properties of a covalent modification cycle are altered by a downstream target. Proc Natl Acad Sci U S A 107: 10032–10037. 20479260 10.1073/pnas.0913815107 PMC2890436

[24] Jiang P, Ventura AC, Sontag ED, Merajver SD, Ninfa AJ, et al (2011) Load-induced modulation of signal transduction networks. Sci Signal 4: ra67. 21990429 10.1126/scisignal.2002152 PMC8760836

[25] Kim KH, Sauro HM (2010) Fan-out in gene regulatory networks. J Biol Eng 4: 16–16. 21167053 10.1186/1754-1611-4-16 PMC3024275

[26] Bluthgen N, Bruggeman FJ, Legewie S, Herzel H, Westerhoff HV, et al (2006) Effects of sequestration on signal transduction cascades. The FEBS journal 273: 895–906. 16478465 10.1111/j.1742-4658.2006.05105.x

[27] Jayanthi S, Del Vecchio D (2012) Tuning genetic clocks employing DNA binding sites. PLoS One 7: e41019. 22859962 10.1371/journal.pone.0041019 PMC3409220

[28] Pomerening JR, Sontag ED, Ferrell JE Jr (2003) Building a cell cycle oscillator: hysteresis and bistability in the activation of Cdc2. Nat Cell Biol 5: 346–351. 12629549 10.1038/ncb954

[29] Prasad A, Zikherman J, Das J, Roose JP, Weiss A, et al (2009) Origin of the sharp boundary that discriminates positive and negative selection of thymocytes. Proc Natl Acad Sci U S A 106: 528–533. 19098101 10.1073/pnas.0805981105 PMC2626737

[30] Das J, Ho M, Zikherman J, Govern C, Yang M, et al (2009) Digital signaling and hysteresis characterize ras activation in lymphoid cells. Cell 136: 337–351. 19167334 10.1016/j.cell.2008.11.051 PMC2662698

[31] Bagowski CP, Ferrell JE Jr (2001) Bistability in the JNK cascade. Curr Biol 11: 1176–1182. 11516948 10.1016/s0960-9822(01)00330-x

[32] Arkin A, Ross J, McAdams HH (1998) Stochastic kinetic analysis of developmental pathway bifurcation in phage lambda-infected Escherichia coli cells. Genetics 149: 1633–1648. 9691025 10.1093/genetics/149.4.1633 PMC1460268

[33] Ferrell JE Jr (1996) Tripping the switch fantastic: how a protein kinase cascade can convert graded inputs into switch-like outputs. Trends Biochem Sci 21: 460–466. 9009826 10.1016/s0968-0004(96)20026-x

[34] Maamar H, Dubnau D (2005) Bistability in the Bacillus subtilis K-state (competence) system requires a positive feedback loop. Molecular microbiology 56: 615–624. 15819619 10.1111/j.1365-2958.2005.04592.x PMC3831615

[35] Legewie S, Bluthgen N, Herzel H (2006) Mathematical modeling identifies inhibitors of apoptosis as mediators of positive feedback and bistability. PLoS computational biology 2: e120. 16978046 10.1371/journal.pcbi.0020120 PMC1570177

[36] Kobayashi H, Kaern M, Araki M, Chung K, Gardner TS, et al (2004) Programmable cells: interfacing natural and engineered gene networks. Proc Natl Acad Sci U S A 101: 8414–8419. 15159530 10.1073/pnas.0402940101 PMC420408

[37] Jayanthi S, Nilgiriwala KS, Del Vecchio D (2013) Retroactivity controls the temporal dynamics of gene transcription. ACS synthetic biology 2: 431–441. 23654274 10.1021/sb300098w

[38] Kuhlman T, Zhang Z, Saier MH Jr, Hwa T (2007) Combinatorial transcriptional control of the lactose operon of Escherichia coli. Proceedings of the National Academy of Sciences of the United States of America 104: 6043–6048. 17376875 10.1073/pnas.0606717104 PMC1851613

[39] Kim K-Y, Wang J (2007) Potential energy landscape and robustness of a gene regulatory network: toggle switch. PLoS Comput Biol 3: e60. 17397255 10.1371/journal.pcbi.0030060 PMC1848002

[40] Wang J, Zhang J, Yuan Z, Zhou T (2007) Noise-induced switches in network systems of the genetic toggle switch. BMC Syst Biol 1: 50–50. 18005421 10.1186/1752-0509-1-50 PMC2214838

[41] Tian T, Burrage K (2006) Stochastic models for regulatory networks of the genetic toggle switch. Proc Natl Acad Sci U S A 103: 8372–8377. 16714385 10.1073/pnas.0507818103 PMC1482501

[42] Warren PB, ten Wolde PR (2005) Chemical models of genetic toggle switches. J Phys Chem B 109: 6812–6823. 16851767 10.1021/jp045523y

[43] Wang J, Xu L, Wang E, Huang S (2010) The Potential Landscape of Genetic Circuits Imposes the Arrow of Time in Stem Cell Differentiation. Biophysical Journal 99: 29–39. 20655830 10.1016/j.bpj.2010.03.058 PMC2895388

[44] Strasser M, Theis FJ, Marr C (2012) Stability and multiattractor dynamics of a toggle switch based on a two-stage model of stochastic gene expression. Biophys J 102: 19–29. 22225794 10.1016/j.bpj.2011.11.4000 PMC3250690

[45] Allen RJ, Warren PB, Ten Wolde PR (2005) Sampling rare switching events in biochemical networks. Phys Rev Lett 94: 018104–018104. 15698138 10.1103/PhysRevLett.94.018104

[46] Wang J, Xu L, Wang E (2008) Potential landscape and flux framework of nonequilibrium networks: robustness, dissipation, and coherence of biochemical oscillations. Proceedings of the National Academy of Sciences of the United States of America 105: 12271–12276. 18719111 10.1073/pnas.0800579105 PMC2527901

[47] Buchler NE, Louis M (2008) Molecular titration and ultrasensitivity in regulatory networks. Journal of molecular biology 384: 1106–1119. 18938177 10.1016/j.jmb.2008.09.079

[48] Lee TH, Maheshri N (2012) A regulatory role for repeated decoy transcription factor binding sites in target gene expression. Molecular Systems Biology 8: 576. 22453733 10.1038/msb.2012.7 PMC3322331

[49] Raj A, Van Oudenaarden A (2008) Nature, nurture, or chance: stochastic gene expression and its consequences. Cell 135: 216–226. 18957198 10.1016/j.cell.2008.09.050 PMC3118044

[50] Shoval O, Goentoro L, Hart Y, Mayo A, Sontag E, et al (2010) Fold-change detection and scalar symmetry of sensory input fields. Proc Natl Acad Sci USA 107: 15995–16000. 20729472 10.1073/pnas.1002352107 PMC2936624

[51] Goentoro L, Shoval O, Kirschner MW, Alon U (2009) The incoherent feedforward loop can provide fold-change detection in gene regulation. Mol Cell 36: 894–899. 20005851 10.1016/j.molcel.2009.11.018 PMC2896310

[52] Kim Y, Paroush Z, Nairz K, Hafen E, Jimenez G, et al (2011) Substrate-dependent control of MAPK phosphorylation in vivo. Mol Syst Biol 7: 467. 21283143 10.1038/msb.2010.121 PMC3063690

[53] Barton JP, Sontag ED (2013) The energy costs of insulators in biochemical networks. Biophys J 104: 1380–1390. 23528097 10.1016/j.bpj.2013.01.056 PMC3602777

