# Supplementary figures and images for: Loads Bias Genetic and Signaling Switches in Synthetic and Natural Systems
Source: PLoS Comput Biol. 2014 Mar 27;10(3):e1003533. doi: 10.1371/journal.pcbi.1003533 (PMC3967935; doi:10.1371/journal.pcbi.1003533)

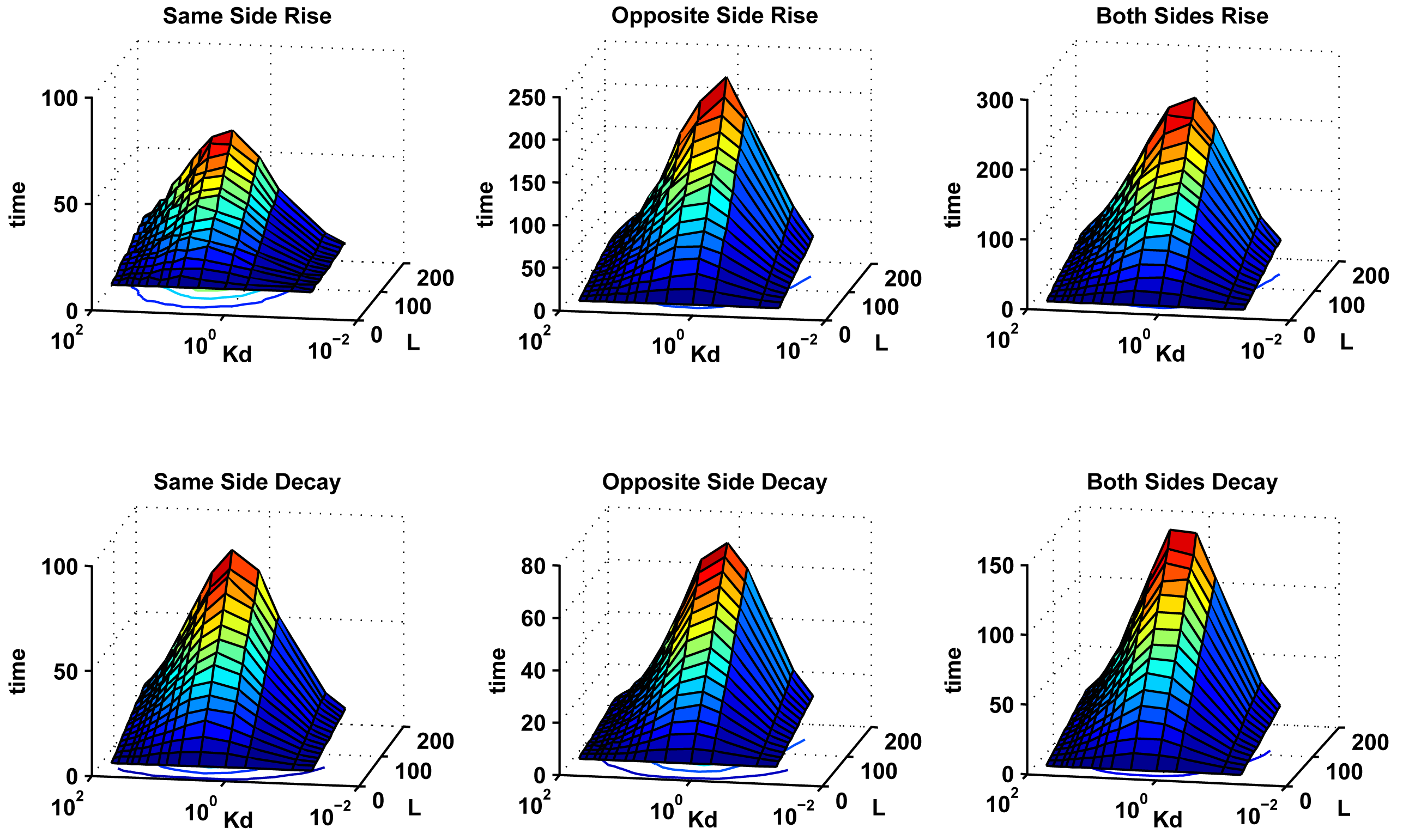

Supplement: Figure S1 — Surface plots showing response times of the simple genetic toggle switch with changes in load (L) and changes in the dissociation constant (Kd) of binding with load. The units of L and Kd are (molecules/µm3). The z-axis measures the response time indicated in the title. “Same Side Rise” and “Same Side Decay” refers to the rise time and decay time when the load is on the same side as the repressor whose concentration is increasing. “Opposite Side Rise” and “Opposite Side Decay” refers to the rise time and the decay time when the load is on the other side of the repressor whose concentration is increasing. “Both Sides Rise” or decay refer to the rise and decay times when a load is present on both sides (symmetrically). The plot shows that at every Kd, the relation between the response time and load is approximately linear. The response time is largest for the case of “Both Sides Rise” followed by “Opposite Side Rise”. The response time is also non-monotonic with respect to the Kd for a given load, and is maximized at intermediate values of Kd. (TIF) [file pcbi.1003533.s001.tif]

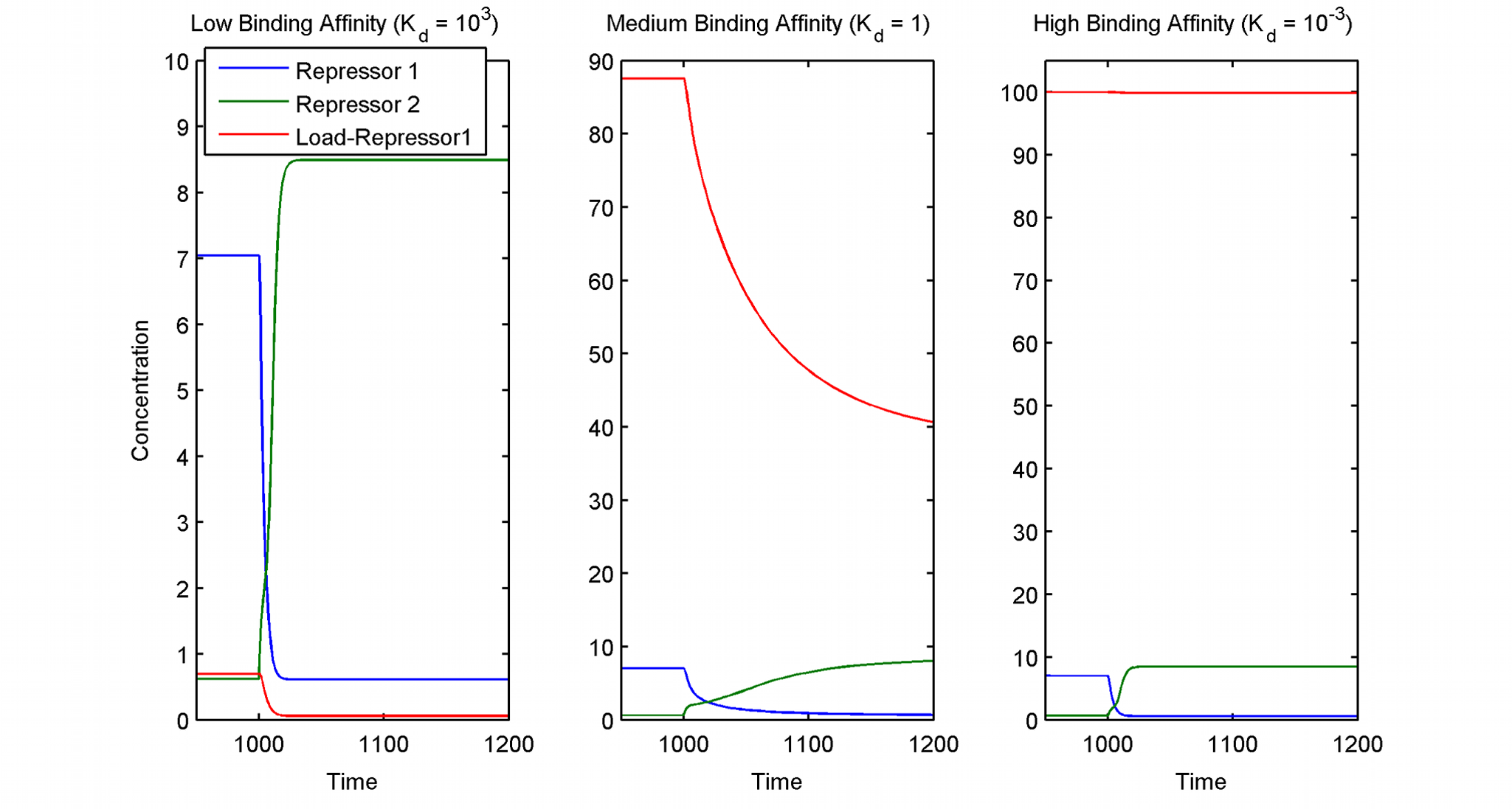

Supplement: Figure S2 — Time plot of switching of the simple toggle switch with a load on Repressor 1, at three different values of the dissociation constant. In all three cases the system is switched by providing 150 molecules/µm3 of an inducer at 1000 minutes. The inducer stays constant at that value and is not shown in the plots. The left panel has a very high dissociation constant (Kd = 1000 molecules/µm3) of binding between the load and the repressor, due to which the load has a minimal effect on the system. The middle panel has an intermediate value (Kd = 1 molecules/µm3) because of which the load acts as a dynamic sink by releasing Repressor 1 and slowing the switching. The right panel shows the effect of a small dissociation constant (Kd = 10−3 molecules/µm3). At such strong binding affinities, all of the load is always bound to Repressor 1. Thus the load has minimal effect on the switching dynamics. In all cases total load concentration is 100 molecules/µm3. (TIF) [file pcbi.1003533.s002.tif]

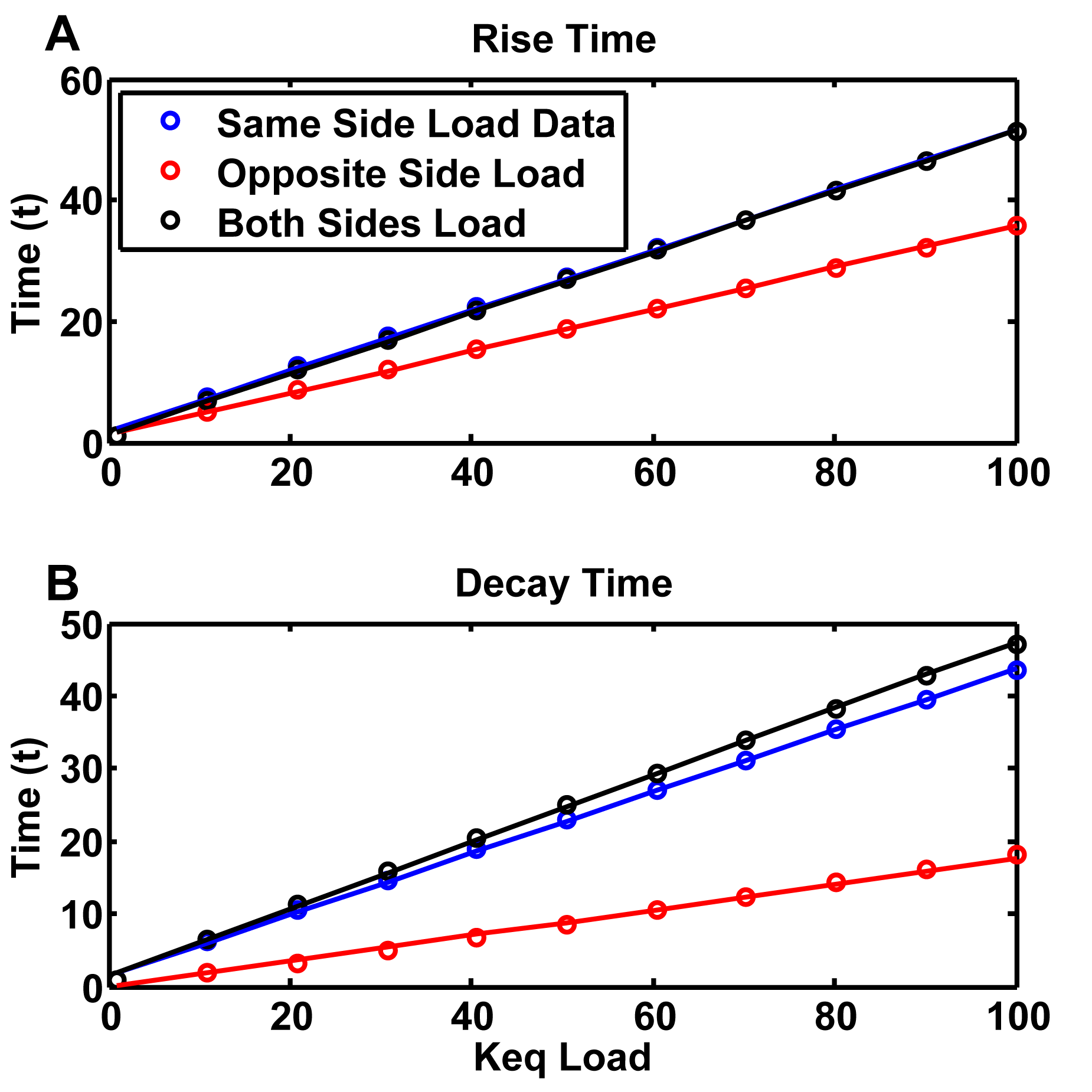

Supplement: Figure S3 — Effects of a dynamic load on dynamics of a symmetric toggle switch. (A). The time taken to reach 90% of maximum value for the protein undergoing a low-to-high transition as a function of the equilibrium constant of a dynamic load. Normalized time is a unit-less number defined by the transition time (rise or decay) of the system at a given loading condition divided by the transition time (rise or decay) of an unloaded system. (B). The time taken for the concentration of the protein undergoing a high-to-low transition to reach 10% of its maximum value. (TIF) [file pcbi.1003533.s003.tif]

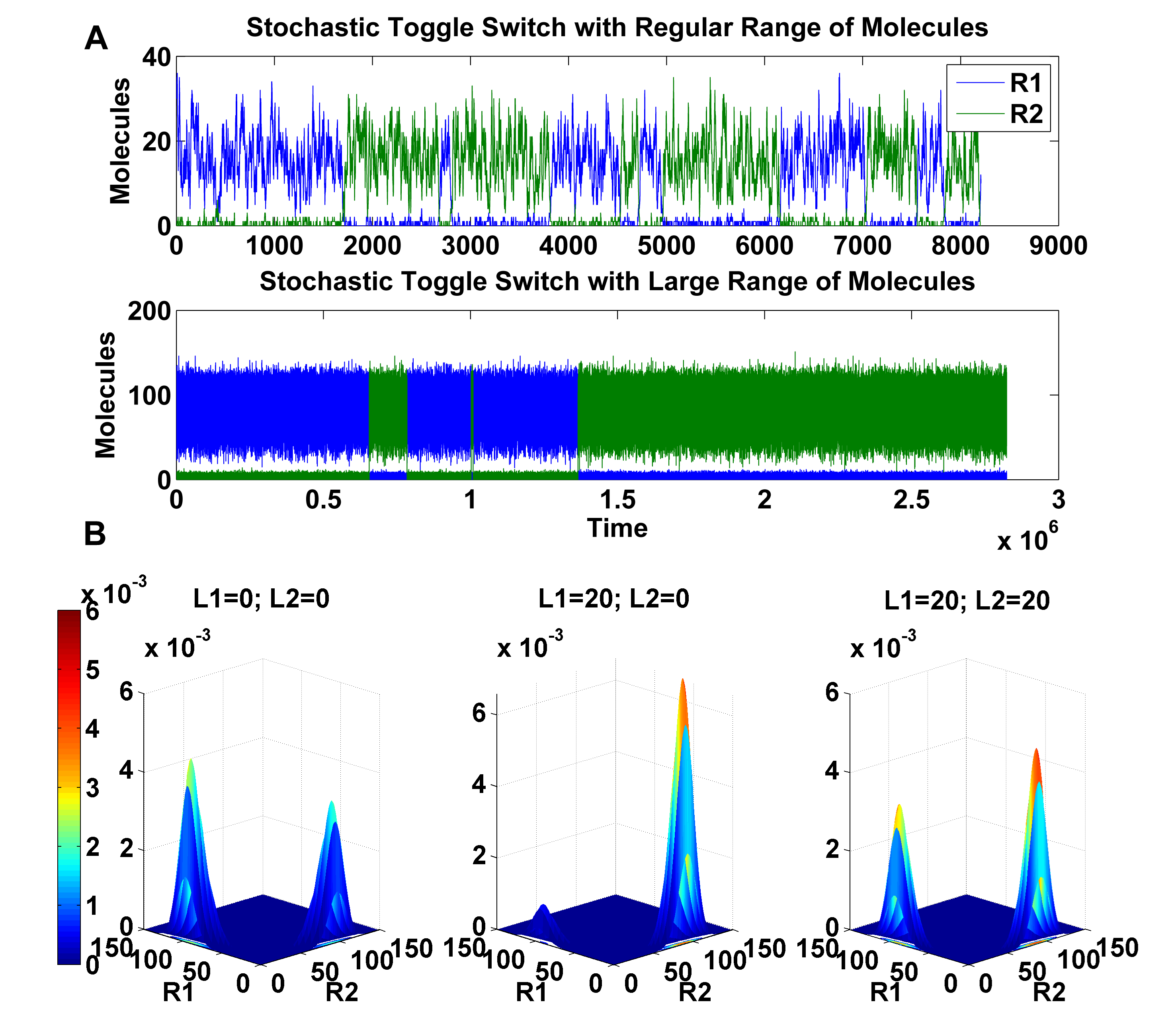

Supplement: Figure S4 — Stochastic time trace and the probability distribution function of repressor concentrations for the large volume simulations. (A). Comparison of time traces of the stochastic simulations of the simple toggle switch with basal parameters (top panel) and a larger volume (bottom panel). The average molecule number is about 5 times greater, and the number of transitions are significantly fewer. (B). The probability distribution function of the genetic toggle switch with the larger molecular number without (left) and with (right) a load. The effect of a load on R1 is qualitatively the same for this system as for the smaller system. Since transitions are slower the data are more uneven for this simulation. (TIF) [file pcbi.1003533.s004.tif]

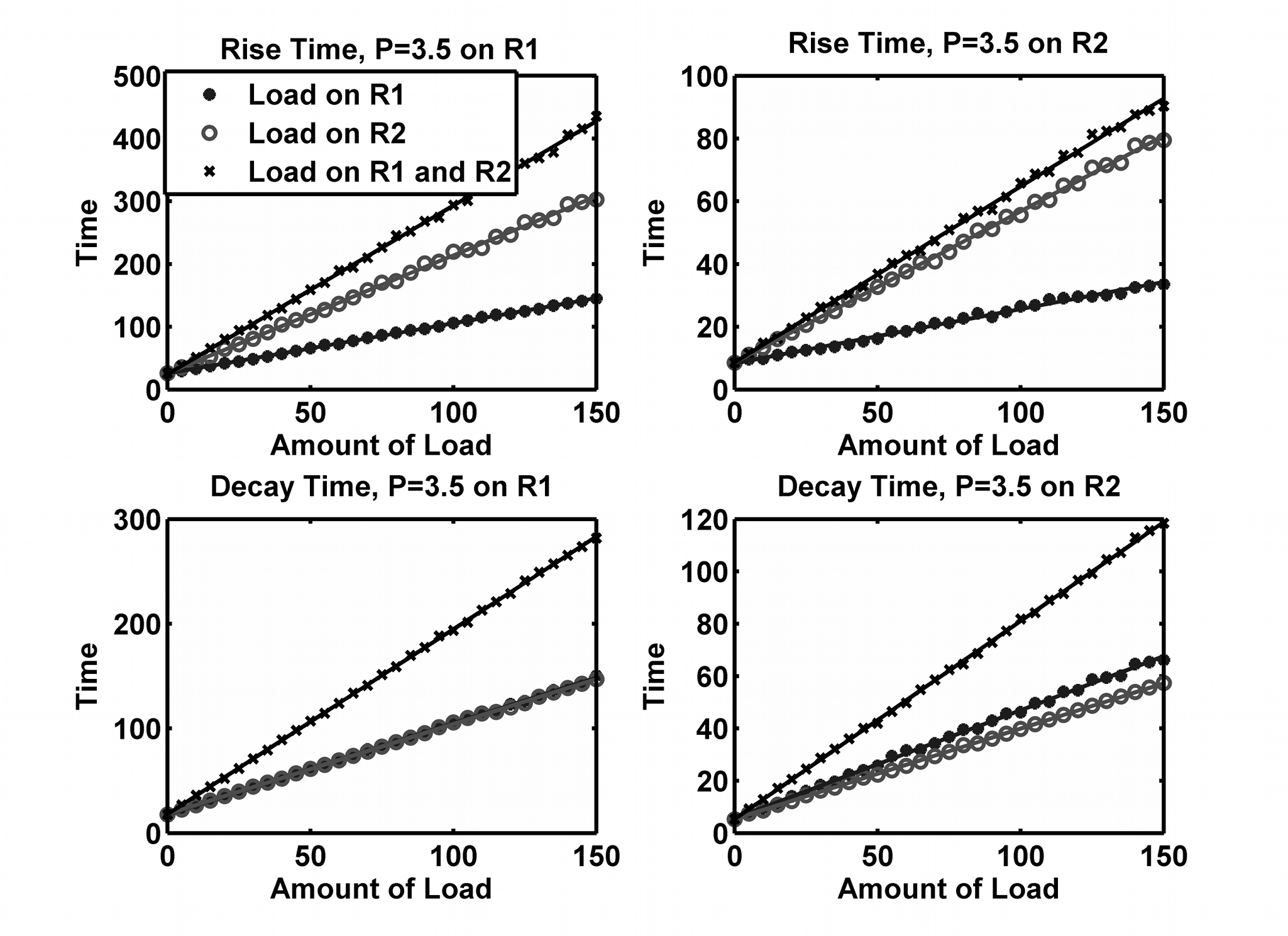

Supplement: Figure S5 — Transition times in a genetic toggle switch with a positive feedback moiety. In all cases the strength of the positive feedback (denoted here by P instead of ρ) is 3.5 on either Repressor 1 (R1) or Repressor 2 (R2). Top Left: Rise time - time to transition INTO state R1 with the positive feedback on R1. Note that the rise time is larger at nonzero loads when the load is on R2 or when the load is on both sides, in agreement with the simple toggle switch. Top Right: Rise time - time to transition INTO state R1 with the positive feedback on R2. Bottom Left: Decay time - time to transition OUT OF state R1 with the positive feedback on R1. Bottom Right: Decay time - time to transition OUT OF state R1 with the positive feedback on R2. (TIF) [file pcbi.1003533.s005.tif]

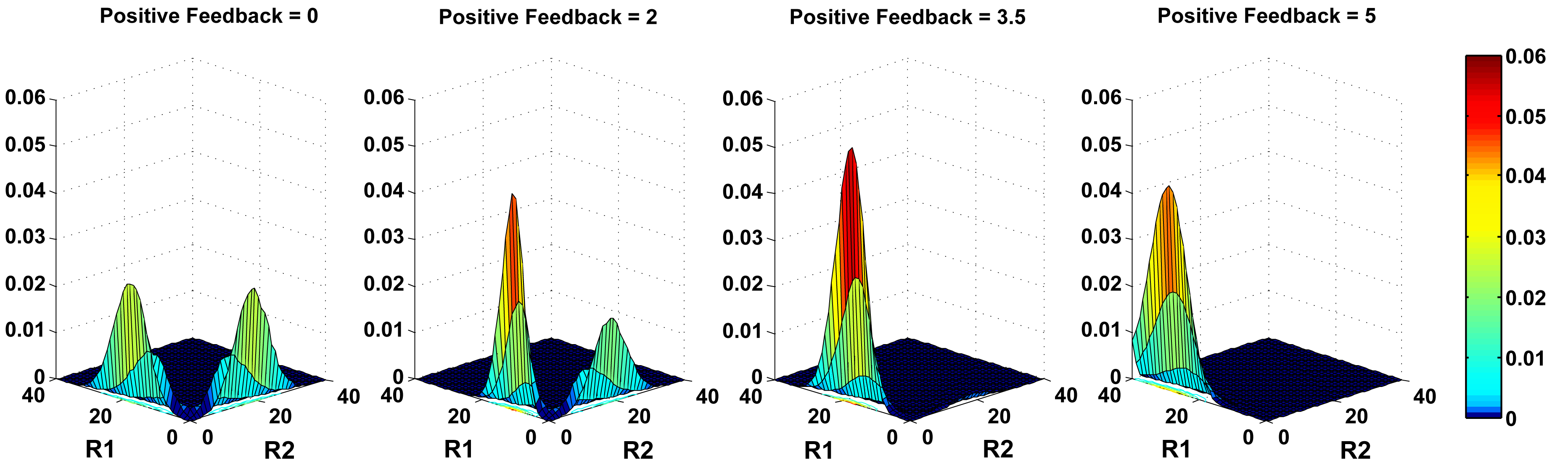

Supplement: Figure S6 — Probability distribution functions of repressor concentrations for the toggle with a positive feedback moiety. The left panel shows that when ρ = 0, the switch is balanced evenly. As ρ increases, the side of the switch with the positive feedback becomes more and more prominent, at the expense of the other side. When ρ = 5, the system spends most of its time in one state. (TIF) [file pcbi.1003533.s006.tif]

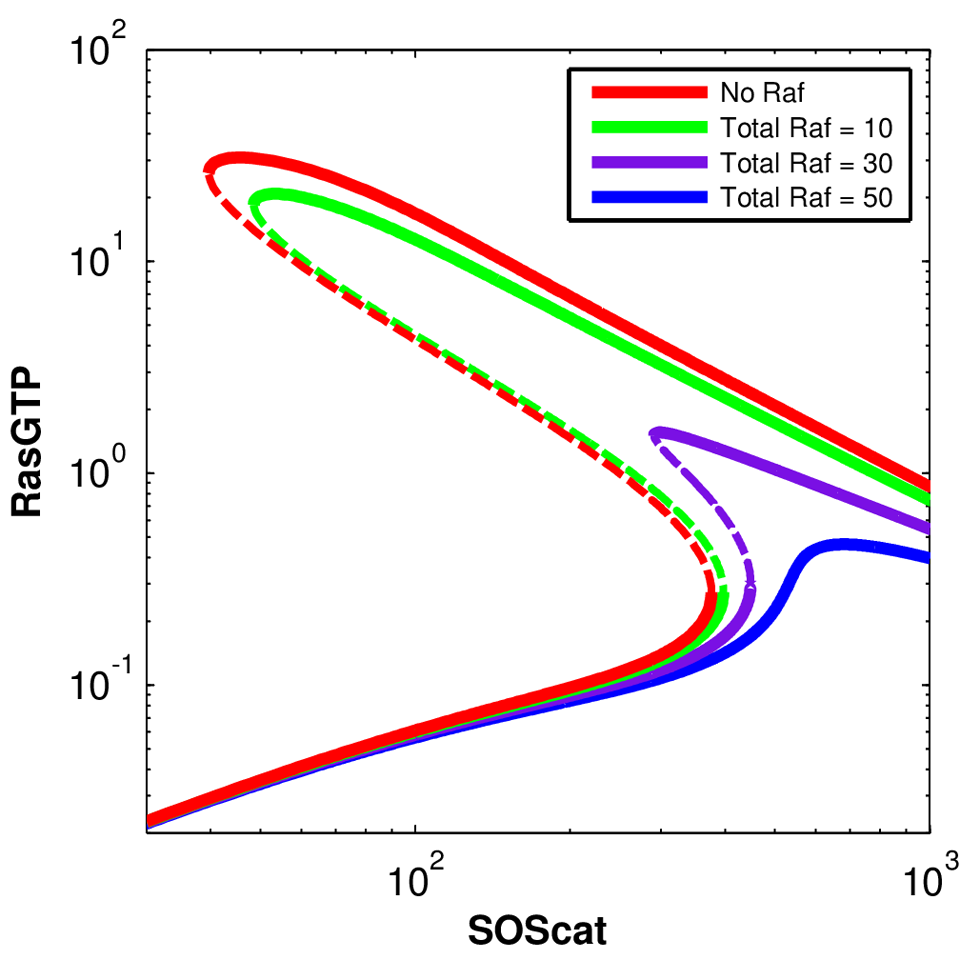

Supplement: Figure S7 — Bifurcation diagram of the Ras switch with different levels of Raf (load) on the system for the model with Pseudo Steady State Assumption (PSSA). The total number of SOS in the simulation box is used as the parameter being tuned, which varies from 0 to 1000. For Raf = 0, Raf = 10 and Raf = 30, there are two bifurcations points as SOS is increased. In the first bifurcation a new high valued stable steady state appears along with the low valued stable steady state. In the second bifurcation, the low valued stable state disappears leaving behind only the high valued state. The dotted line marks the unstable steady state that also comes into existence in the bistable region. As total Raf increases, the two bifurcations approach each other. When Raf = 50, the system has lost both of its bifurcations and is characterized by a single stable steady state at all values of Raf. (TIF) [file pcbi.1003533.s007.tif]

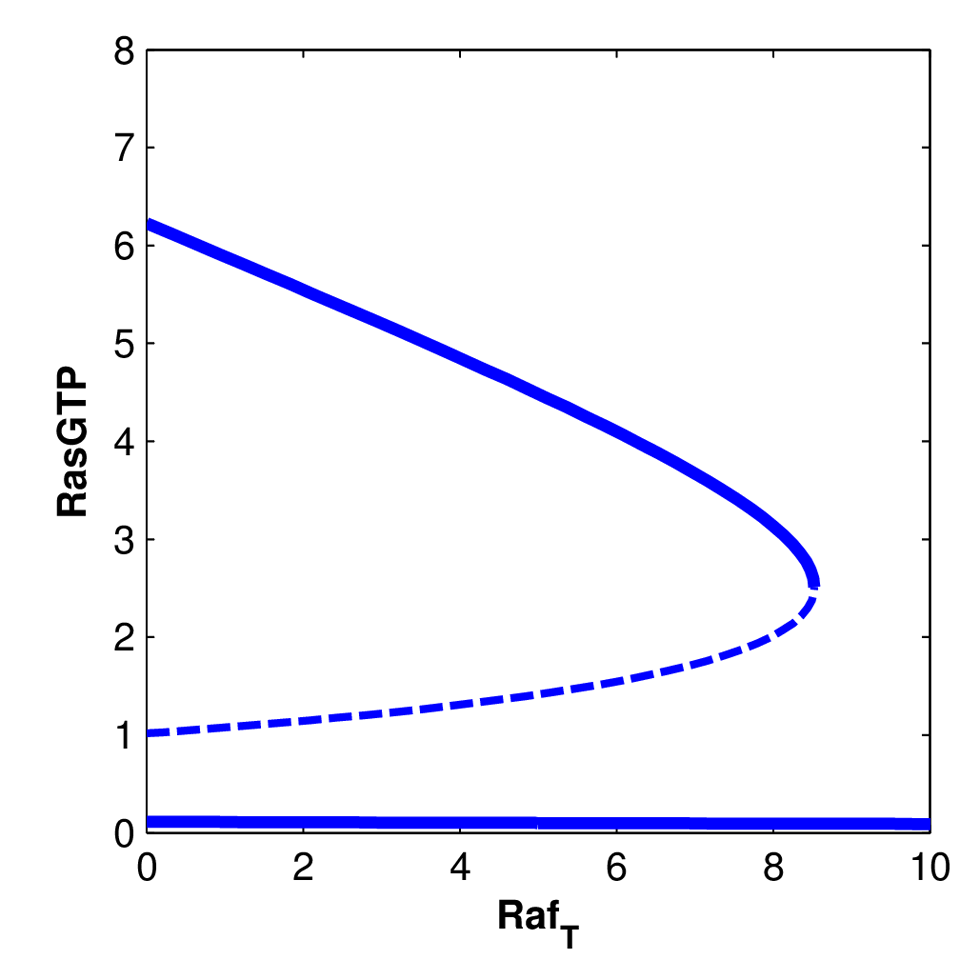

Supplement: Figure S8 — Bifurcation diagram of the Ras activation model based on Law of Mass Action (LMA). Here the total number of Raf molecules (RafT) is the primary parameter being varied. Without Raf, the Ras activation system is bistable as reported. With increasing RafT, the “high” stable steady state branch comes closer with the unstable steady state branch and both are eliminated after a threshold of RafT. A monostable region is maintained beyond the threshold. (TIF) [file pcbi.1003533.s008.tif]

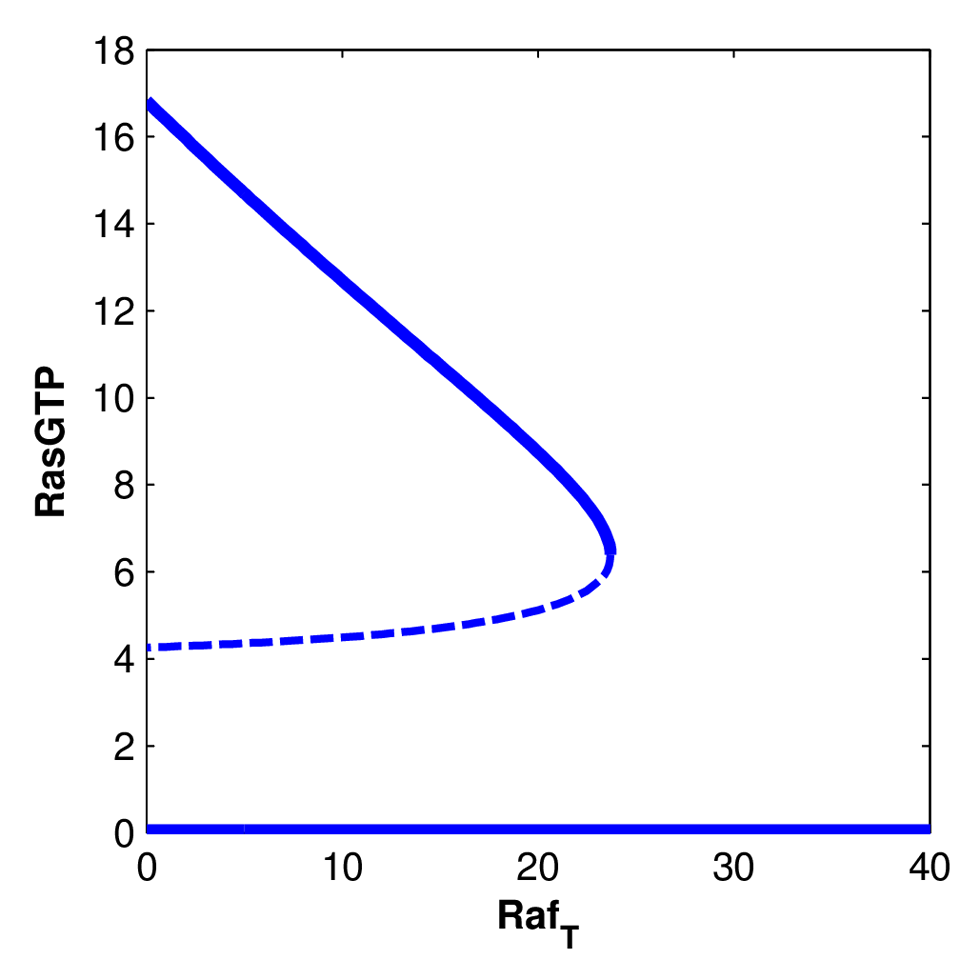

Supplement: Figure S9 — Bifurcation diagram of the Ras activation PSSA model with total number of Raf molecules (RafT) as the primary parameter. Without Raf, the Ras activation system is bistable as reported. With increasing RafT, the “high” stable steady state branch comes closer with the unstable steady state branch and both are eliminated after a threshold of RafT. A monostable region is maintained beyond the threshold. (TIF) [file pcbi.1003533.s009.tif]

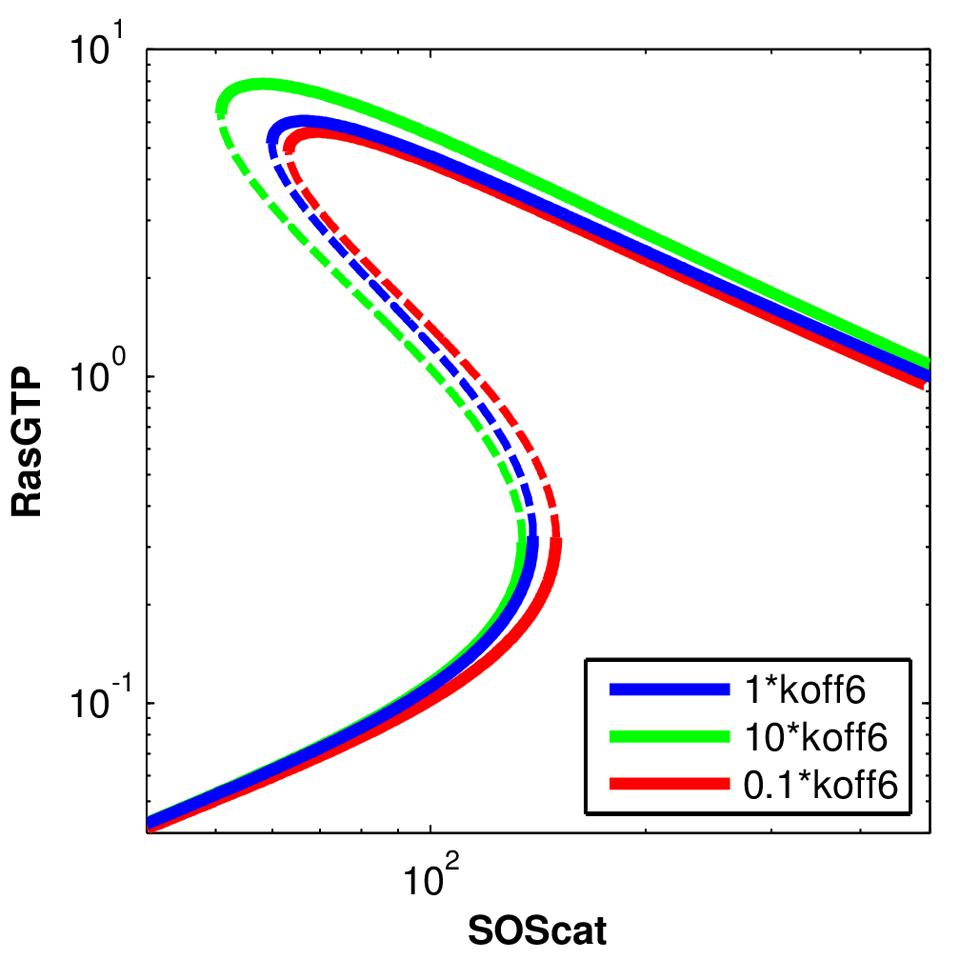

Supplement: Figure S10 — Parameter sensitivity of the bistability of Ras switch to changes in koff6. Increase of koff6 results in leftward shifts of both stable fixed points, increase in the bistable regime and increase in maximal RasGTP activation level (Green Line) when compared to baseline with original value (Blue Line). Decrease of koff6 (Red Line) results in right shift of both limit points, increase in unstable bistable regime and decrease in maximal RasGTP activation level. Qualitative features of bistability are maintained. (TIF) [file pcbi.1003533.s010.tif]

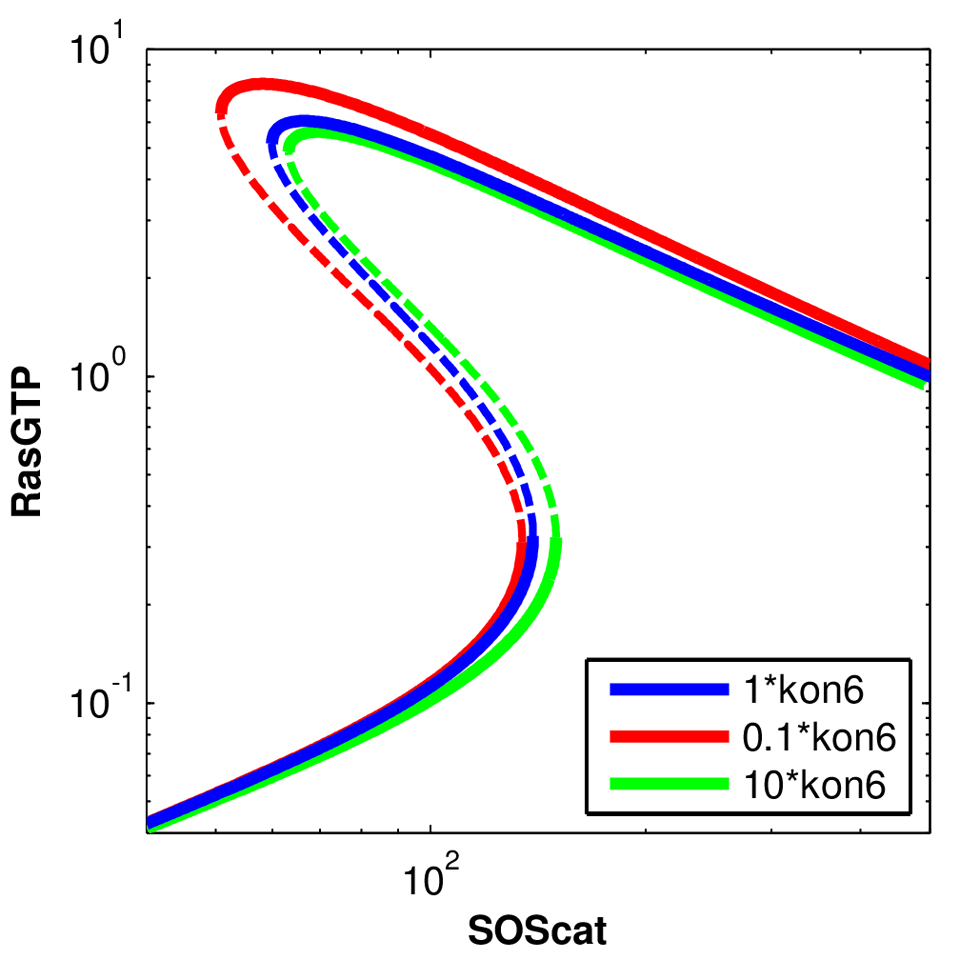

Supplement: Figure S11 — Parameter sensitivity of the bistability of Ras switch to changes in kon6. Increase of kon6 (Green Line) results in right shift of both limit points, increase in unstable bistable regime and decrease in maximal RasGTP activation level when compared to baseline original value (Blue Line). Decrease of kon6 (Red Line) results in left shifts of both limit points, increase in bistable regime and increase in maximal RasGTP activation level. Qualitative features of bistability are maintained. (TIF) [file pcbi.1003533.s011.tif]

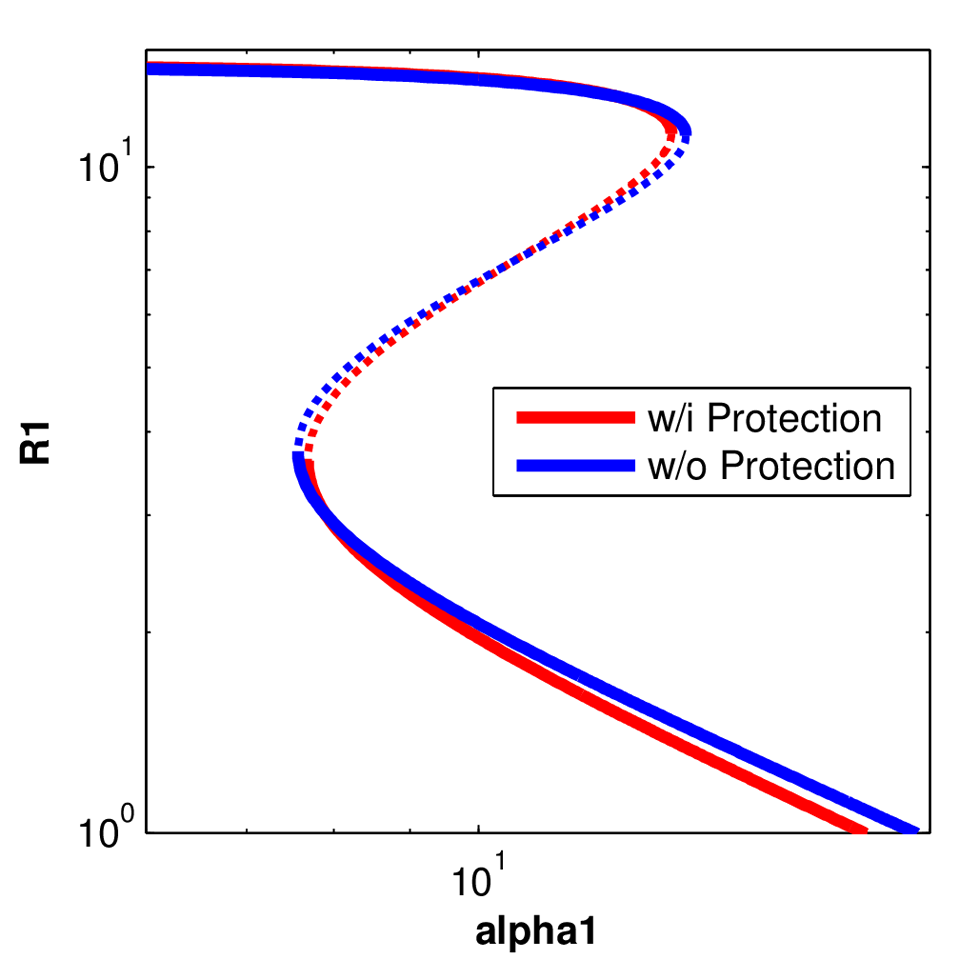

Supplement: Figure S12 — Comparison between bifurcation diagrams of toy genetic toggle switch with and without protection of repressor degradation when bound with promoters. If the protection is not included (Blue Line), a minor increase in the bistable region can be observed with right shift of upper limit point and left shift of lower limit point compared to the case with protection assumed (Red Line). Note that this is not the same as degradation after being bound with the load. (TIF) [file pcbi.1003533.s012.tif]

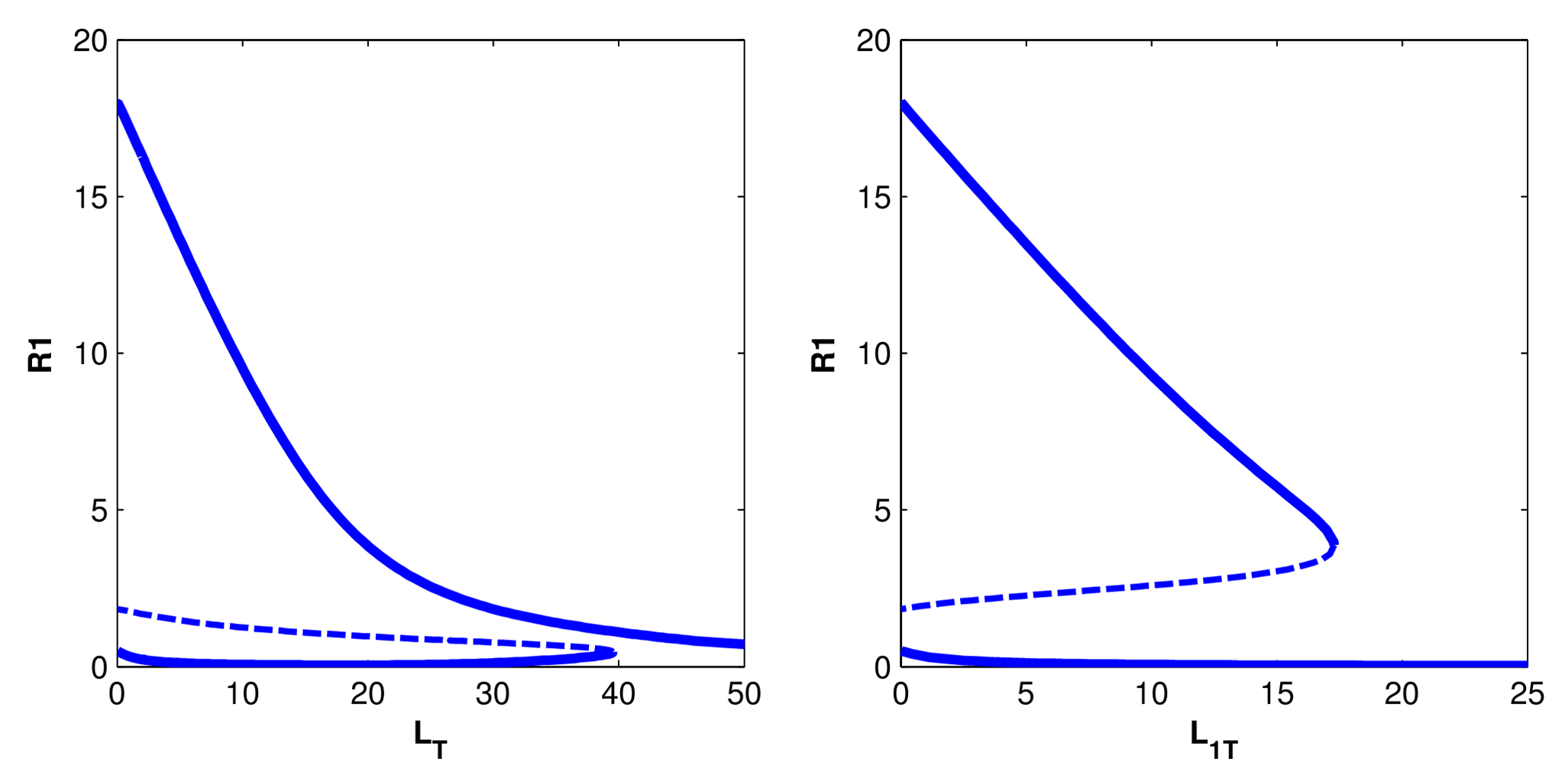

Supplement: Figure S13 — Bifurcation diagram of the genetic toggle switch with positive feedback loop on one side after removal of the protection assumption. The left panel shows the bifurcation diagram when the load is added symmetrically to both sides. Without load molecule, the toggle switch is bistable as predicted. With the increase in LT, the unstable steady state and the “low” stable steady state come closer and meet at certain threshold. The value of “high” stable steady state decreases with increase in LT. Beyond the threshold, the toggle switch becomes monostable. The right panel shows the effect of just adding a load to R1. In this case the high state of R1 approaches the unstable steady state, and annihilates itself. The system jumps to the low stable state, which is equivalent to the “high” state of the other repressor. (TIF) [file pcbi.1003533.s013.tif]

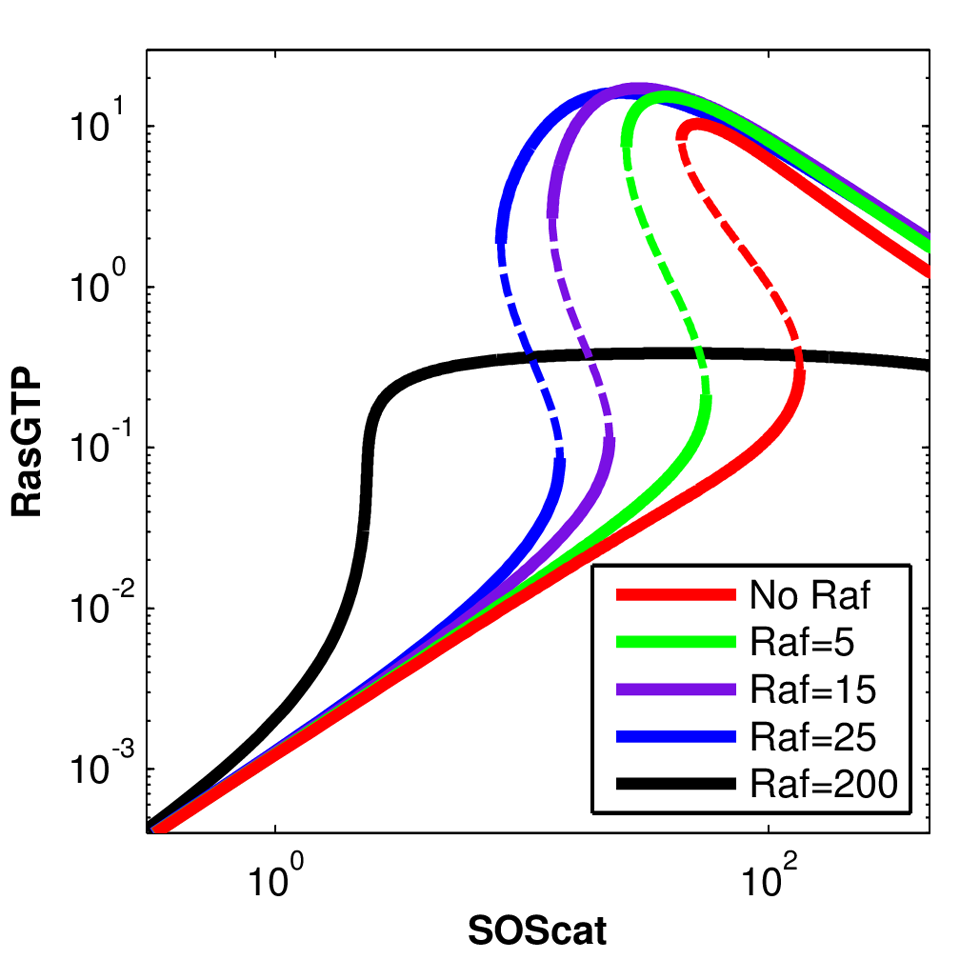

Supplement: Figure S14 — Bifurcation diagram of the Ras activation model when Ras can degrade when bound with Raf. As the number of Raf molecules increase, the bistable region decreases. However unlike the case with no protection, the curve moves to the left. When Raf molecules increase by a large amount, bistability is abrogated. (TIF) [file pcbi.1003533.s014.tif]

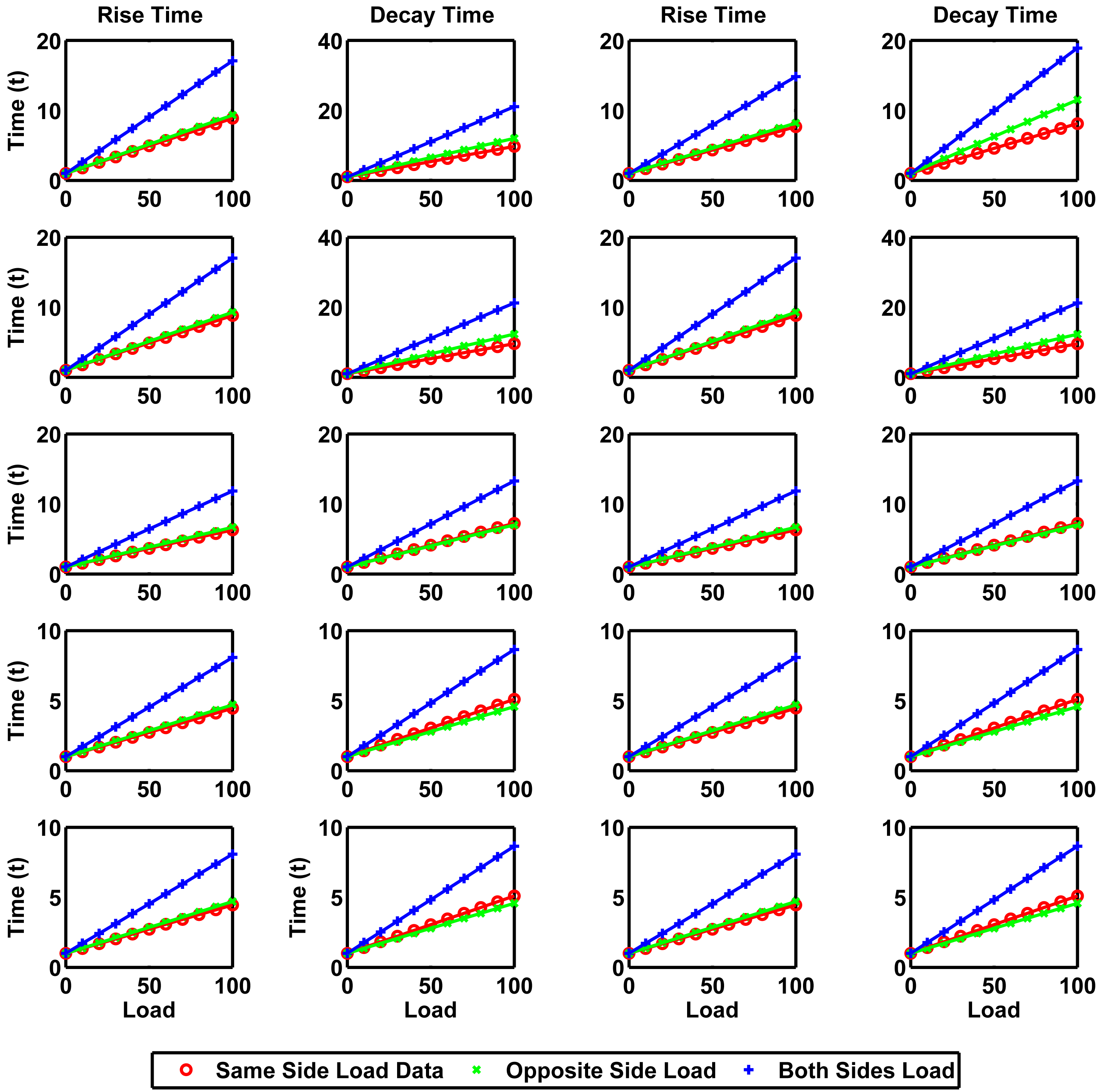

Supplement: Figure S15 — Transition times for various k′on and k′off values plotted as a function of load for the basic toggle switch. Even if the binding-unbinding rates are slower or much faster than protein decay rates, the load-transition time relationship stays linear. A, C, E, G, I, K, M, O, Q and S show the rise time. B, D, F, H, J, L, N, P, R, and T show decay time. (A,B) k′on = 4, k′off = 0.5, Kd = 0.125. (C,D) k′on = 10, k′off = 0.5, Kd = 0.05. (E,F) k′on = 4, k′off = 4, Kd = 1. (G,H) k′on = 10, k′off = 10, Kd = 1. (I,J) k′on = 4, k′off = 20, Kd = 5. (K,L) k′on = 10, k′off = 50, Kd = 5. (M,N) k′on = 4, k′off = 40, Kd = 10. (O,P) k′on = 10, k′off = 100, Kd = 10. (Q,R) k′on = 40, k′off = 400, Kd = 10. (S,T) k′on = 100, k′off = 1000, Kd = 10. (TIF) [file pcbi.1003533.s015.tif]

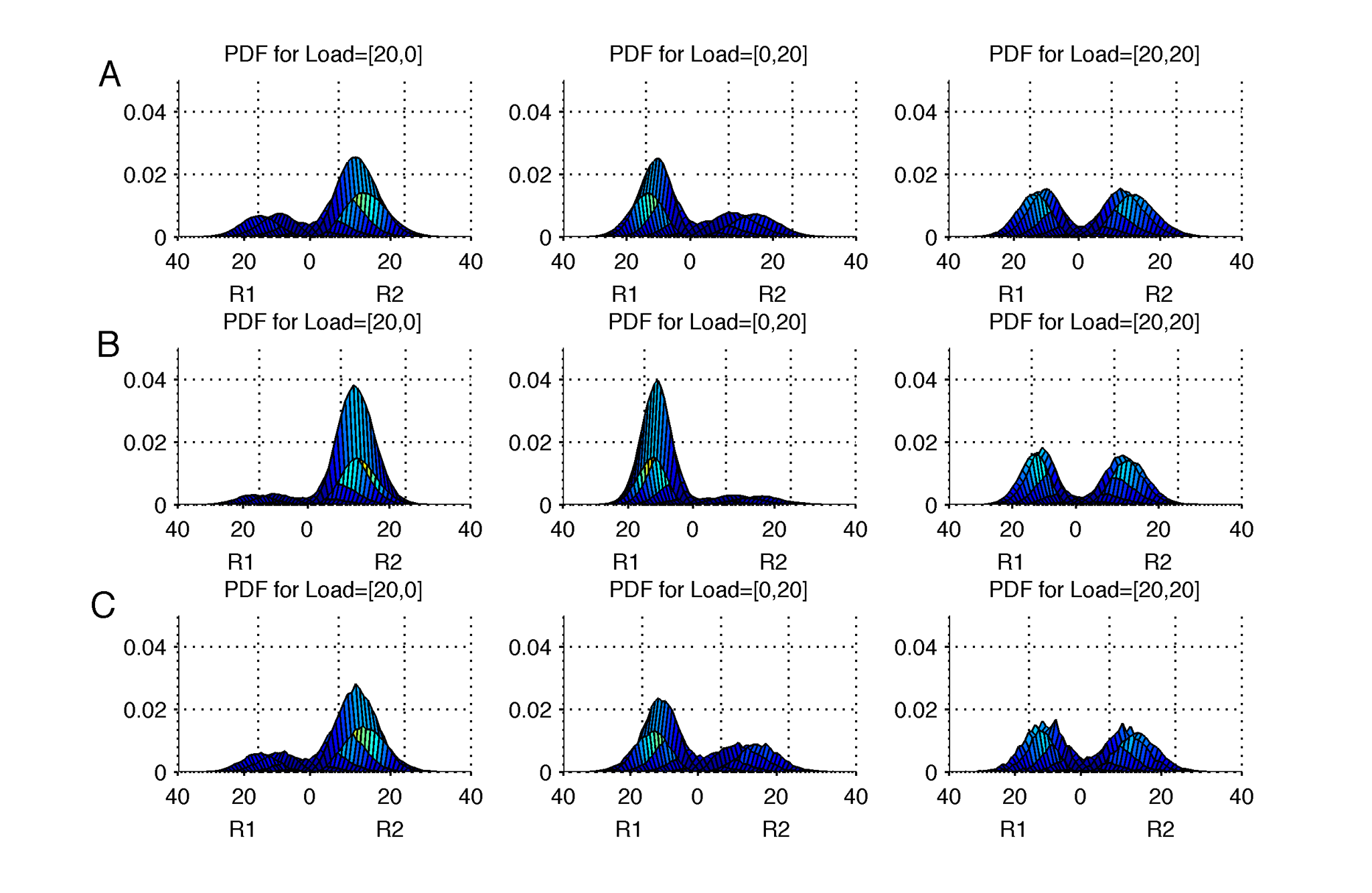

Supplement: Figure S16 — Probability distributions of repressor concentrations for various values of k′on and k′off for the basic toggle switch. Even when the binding-unbinding with the load is several times faster than protein decay rates, the basic phenomena discussed in the paper remains unchanged. (A) k′on = 50, k′off = 500 (B) k′on = 500 k′off = 500 (C) k′on = 500 k′off = 5000. (TIF) [file pcbi.1003533.s016.tif]

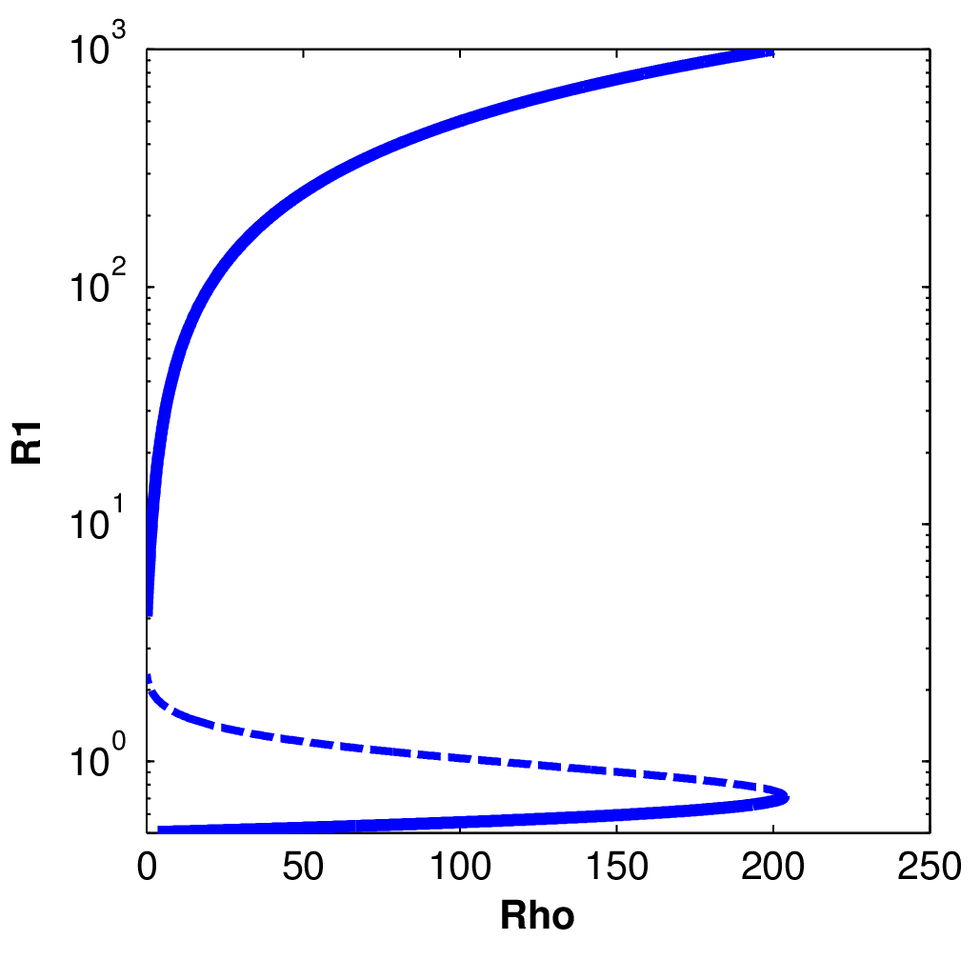

Supplement: Figure S17 — Bistability of the toggle switch with positive feedback. A bifurcation diagram of the simple toggle switch with a positive feedback moiety on one side, with respect to the parameter ρ that measures the strength of the positive feedback. Only the concentration of R1 is shown for simplicity. The switch remains bistable till ρ becomes larger than a little over 200. (TIF) [file pcbi.1003533.s017.tif]
