# Supplementary material for: Loads Bias Genetic and Signaling Switches in Synthetic and Natural Systems
Source: PLoS Comput Biol. 2014 Mar 27;10(3):e1003533. doi: 10.1371/journal.pcbi.1003533 (PMC3967935; doi:10.1371/journal.pcbi.1003533)
Supplement: Table S1 — Slopes of linear fits to rise and decay time with various values of Koff, Kon and β. The first column reports the values of the dissociation constant (Kd = Koff/Kon) and the kinetic constants of the binding of Repressor 1, 2 or the value for β, which represents promoter strength. The other columns report the slopes of the linear fits of the various rise times and decay times. In all cases the fits have high R-squared values (>0.95). Intercept is 1, as the slopes are normalized to the un-loaded transition time. For Kd we change the parameters by two orders of magnitude in both directions to show that the linear relation is robust despite these changes. Note that the relation between rise time or decay time and the binding constant is non-monotonic. Units are as reported in the text. (DOC) [file pcbi.1003533.s018.doc]

Table S1 Slopes of linear fits to rise and decay time with various values of K­_off_, K_on_ and β

|  | Rise Time | | | Decay Time | | |
| --- | --- | --- | --- | --- | --- | --- |
|  | Same-Sided | Opposite Side | Both Sides | Same-Sided | Opposite Side | Both Sides |
| Koff=0.005; Kon=0.5; Kd= 0.01 | 4.59E-03 | 2.12E-03 | 6.72E-03 | 1.76E-03 | 1.01E-02 | 1.19E-02 |
| Koff=0.05; Kon=0.5; Kd= 0.1 | 2.46E-02 | 2.75E-02 | 5.19E-02 | 2.50E-02 | 4.47E-02 | 7.10E-02 |
| Koff=0.5; Kon=0.5; Kd= 1 | 7.80E-02 | 8.32E-02 | 1.60E-01 | 8.59E-02 | 1.13E-01 | 2.03E-01 |
| Koff=5; Kon=0.5; Kd= 10 | 3.46E-02 | 3.71E-02 | 7.08E-02 | 4.11E-02 | 3.57E-02 | 7.66E-02 |
| Koff=50; Kon=0.5; Kd= 100 | 4.68E-03 | 4.98E-03 | 9.62E-03 | 5.51E-03 | 4.25E-03 | 9.72E-03 |
| Koff=0.5; Kon=50; Kd= 0.01 | 2.50E-04 | 3.93E-04 | 6.44E-04 | 3.34E-04 | 4.68E-04 | 8.01E-04 |
| Koff=0.5; Kon=5; Kd= 0.1 | 2.83E-03 | 3.31E-03 | 6.14E-03 | 3.05E-03 | 5.36E-03 | 8.41E-03 |
| Koff=0.5; Kon=0.5; Kd= 1 | 7.80E-02 | 8.32E-02 | 1.60E-01 | 8.59E-02 | 1.13E-01 | 2.03E-01 |
| Koff=0.5; Kon=0.05; Kd= 10 | 3.45E-02 | 3.80E-02 | 7.15E-02 | 4.28E-02 | 3.47E-02 | 7.77E-02 |
| Koff=0.5; Kon=0.005; Kd= 100 | 5.29E-03 | 5.24E-03 | 1.07E-02 | 5.89E-03 | 4.12E-03 | 1.03E-02 |
| β1= β2 = 0.4 | 7.22E-02 | 1.48E-02 | 1.42E-01 | 2.08E-01 | 3.76E-02 | 2.46E-01 |
| β1= β2 = 4 | 3.69E-03 | 1.62E-01 | 1.50E-01 | 5.29E-03 | 8.70E-02 | 9.76E-02 |
| β1= β2 = 40 | - | 2.09E-01 | 2.02E-01 | 3.68E-05 | 9.20E-02 | 9.23E-02 |

The first column reports the values of the dissociation constant (Kd = Koff/Kon) and the kinetic constants of the binding of Repressor1, 2 or the value for β. The other columns report the slopes of the linear fits of the various rise times and decay times. In all cases the fits have high R-squared values ( > 0.95). Intercept is 1, as the slopes are normalized to the un-loaded transition time. For K_d_ we change the parameters by two orders of magnitude in both directions to show that the linear relation is robust despite these changes. Note that the relation between rise time or decay time and the binding constant is non-monotonic. Units are as reported in the text.
