# Supplementary material for: Loads Bias Genetic and Signaling Switches in Synthetic and Natural Systems
Source: PLoS Comput Biol. 2014 Mar 27;10(3):e1003533. doi: 10.1371/journal.pcbi.1003533 (PMC3967935; doi:10.1371/journal.pcbi.1003533)
Supplement: Table S2 — Exponential Fits of the amount of inducer required to transition states as a function of load. The basic genetic toggle switch switch was toggled to its other state by production of the other repressor protein by an inducer, given here as a bolus with a decay rate as shown. The size of the bolus was increased until the state changed. This was repeated at different levels of load and the minimum size of the bolus required was fit by an exponential function of the load. The fits are shown here, along with their R-squared values. “Load applied to the opposite side” means switching from a state without a load to a state with a load. “Load applied to the same side” means switching from a state with a load to a state without a load. (DOC) [file pcbi.1003533.s019.doc]

Table S2 Exponential Fits of the amount of inducer required to transition states as a function of load.

| Inducer Decay Rate (1/min) | Equation | R^2^ Value |
| --- | --- | --- |
|  | Load Applied to both sides | |
| 0.5 | Inducer = 18.44*exp(0.305*Load) | 0.99971 |
| 0.1 | Inducer = 2.41*exp(0.0629*Load) | 0.99627 |
| 0.05 | Inducer = 2.00*exp(0.0308*Load) | 0.99995 |
| 0.01 | Inducer = 1.69*exp(0.006147*Load) | 0.99271 |
| 0.005 | Inducer = 2.23*exp(0.00294*Load) | 0.99204 |
|  | Load Applied to the “opposite side” | |
| 0.5 | Inducer = 16.80*exp(0.108*Load) | 0.999188 |
| 0.1 | Inducer = 1.94*exp(0.0224*Load) | 0.994335 |
| 0.05 | Inducer = 1.54*exp(0.0111*Load) | 0.993154 |
| 0.01 | Inducer = 1.05*exp(0.00233*Load) | 0.998114 |
| 0.005 | Inducer = 0.972*exp(0.00116*Load) | 0.996343 |
|  | Load Applied to the “same side” | |
| 0.5 | Inducer = 17.7*exp(0.219*Load) | 0.999907 |
| 0.1 | Inducer = 2.34*exp(0.0484*Load) | 0.996021 |
| 0.05 | Inducer = 1.91*exp(0.0239*Load) | 0.997584 |
| 0.01 | Inducer = 1.45*exp(0.00496*Load) | 0.992406 |
| 0.005 | Inducer = 1.46*exp(0.00245*Load) | 0.991991 |

The basic genetic toggle switch switch was toggled to its other state by production of the other repressor protein by an inducer, given here as a bolus with a decay rate as shown. The size of the bolus was increased until the state changed. This was repeated at different levels of load and the minimum size of the bolus required was fit by an exponential function of the load. The fits are shown here, along with their R-squared. “Load applied to the opposite side” means switching from a state without a load to a state with a load. “Load applied to the same side” means switching from a state with a load to a state without a load.
