# Supplementary material for: Loads Bias Genetic and Signaling Switches in Synthetic and Natural Systems
Source: PLoS Comput Biol. 2014 Mar 27;10(3):e1003533. doi: 10.1371/journal.pcbi.1003533 (PMC3967935; doi:10.1371/journal.pcbi.1003533)
Supplement: Table S3 — Exponential Fits of the amount of inducer required to transition states as a function of load, in the case of induction by repression. The switch was toggled to its other state by repression of the current state by an external molecule, given to the system as a bolus with a decay rate as shown. The size of the bolus was increased until the state changed. This was repeated at different levels of load and the minimum size of the bolus required was fit by an exponential function of the load. The fits are shown here, along with their R-squared value. Thus the inducer required depends exponentially on the load in both the methods of induction. “Load applied to the opposite side” means switching from a state without a load to a state with a load. “Load applied to the same side” means switching from a state with a load to a state without a load. (DOC) [file pcbi.1003533.s020.doc]

Table S3 Exponential Fits of the amount of inducer required to transition states as a function of load, in the case of induction by repression

| Inducer Decay Rate | Equation | R^2^ Value |
| --- | --- | --- |
|  | Load Applied to Both Sides | |
| 0.5 | Inducer = 43.56*exp(0.506*Load) | 0.999911 |
| 0.1 | Inducer = 5.61*exp(0.111*Load) | 0.998743 |
| 0.05 | Inducer = 3.90*exp(0.0586*Load) | 0.993461 |
| 0.01 | Inducer = 3.08*exp(0.0117*Load) | 0.991054 |
| 0.005 | Inducer = 3.07*exp(0.00580*Load) | 0.991298 |
|  | Load Applied to Opposite Side | |
| 0.5 | Inducer = 45.13*exp(0.0700 *Load) | 0.999526 |
| 0.1 | Inducer = 5.12*exp(0.0127*Load) | 0.999407 |
| 0.05 | Inducer = 3.42*exp(0.00663*Load) | 0.996845 |
| 0.01 | Inducer = 2.68*exp(0.00130*Load) | 0.99715 |
| 0.005 | Inducer = 2.49*exp(0.000665*Load) | 0.994754 |
|  | Load Applied to Same Side | |
| 0.5 | Inducer = 47.01*exp(0.413*Load) | 0.999474 |
| 0.1 | Inducer = 8.35*exp(0.0839*Load) | 0.993501 |
| 0.05 | Inducer = 5.35*exp(0.0450*Load) | 0.995209 |
| 0.01 | Inducer = 4.98*exp(0.00881*Load) | 0.995697 |
| 0.005 | Inducer = 4.54*exp(0.00450*Load) | 0.993970 |

The switch was toggled to its other state by repression of the current state by an external molecule, given to the system as a bolus with a decay rate as shown. The size of the bolus was increased until the state changed. This was repeated at different levels of load and the minimum size of the bolus required was fit by an exponential function of the load. The fits are shown here, along with their R-squared. Thus the inducer required depends exponentially on the load in both the methods of induction. “Load applied to the opposite side” means switching from a state without a load to a state with a load. “Load applied to the same side” means switching from a state without a load to a state with a load.
