# Supplementary material for: Loads Bias Genetic and Signaling Switches in Synthetic and Natural Systems
Source: PLoS Comput Biol. 2014 Mar 27;10(3):e1003533. doi: 10.1371/journal.pcbi.1003533 (PMC3967935; doi:10.1371/journal.pcbi.1003533)
Supplement: Table S4 — Slopes of linear fits to rise and decay time with a dynamic load, with varying values of load decay rate Kd, load binding rates Kon and Koff, and constant K1. The first four columns report the values of the various parameters. The other columns report the slopes of the linear fits of the various rise times and decay times. In most cases the fits have high R-squared values (>0.95). The two exceptions are >0.90 and starred. Intercept is 1, as the slopes are normalized to the un-loaded transition time. Note that for all cases, the relationship between load (expressed here as Keq = Kb/Kd) and transition time is a positive linear relationship. (DOC) [file pcbi.1003533.s021.doc]

Table S4 Slopes of linear fits to rise and decay time with a dynamic load, with varying values of load decay rate K_d_, load binding rates K_on_ and K_off_, and constant K_1_.

|  |  |  |  | Rise Time | | | Decay Time | | |
| --- | --- | --- | --- | --- | --- | --- | --- | --- | --- |
| Kd | K1 | Kon | Koff | Same | Opposite | Both | Same | Opposite | Both |
| 0.5 | 1 | 0.5 | 0.5 | 0.498 | 0.345 | 0.502 | 0.423 | 0.179 | 0.460 |
| 0.5 | **5** | 0.5 | 0.5 | 1.333 | 0.518 | 0.340 | 0.463 | 0.773 | 0.435 |
| 0.5 | **0.5** | 0.5 | 0.5 | 0.264 | 0.224 | 0.343 | 0.308 | 0.089 | 0.337 |
| **0.05** | 1 | 0.5 | 0.5 | 0.044 | 0.483 | 0.547 | 0.622 | 0.058 | 0.628 |
| **5** | 1 | 0.5 | 0.5 | 0.357 | 0.332 | 0.437 | 0.393 | 0.187 | 0.427 |
| 0.5 | 1 | **5** | 0.5 | 4.367 | 0.540 | 0.520 | 0.535 | 1.767 | 0.578 |
| 0.5 | 1 | **0.05** | 0.5 | 0.062 | 0.059 | 0.100 | 0.093 | 0.014 | 0.105 |
| 0.5 | 1 | 0.5 | **5** | 0.057 | 0.055 | 0.093 | 0.084 | 0.017 | 0.093 |
| 0.5 | 1 | 0.5 | **0.05** | 4.538 | 0.582 | 0.506 | 0.578 | 1.835 | 0.625 |

The first four columns report the values of the various parameters. The other columns report the slopes of the linear fits of the various rise times and decay times. In most cases the fits have high R-squared values ( > 0.95) The two exceptions are >0.90 and starred. Intercept is 1, as the slopes are normalized to the un-loaded transition time. Note that for all cases, the relationship between load (expressed here as K_eq_ = K_b_/K_d_) and transition time is a positive linear relationship.
