# Supplementary material for: Loads Bias Genetic and Signaling Switches in Synthetic and Natural Systems
Source: PLoS Comput Biol. 2014 Mar 27;10(3):e1003533. doi: 10.1371/journal.pcbi.1003533 (PMC3967935; doi:10.1371/journal.pcbi.1003533)
Supplement: Table S5 — Rate expressions used for the stochastic simulations of the genetic toggle switch. The rate expressions used for the stochastic simulation of the toggle switch along with the description of the reaction are listed. (DOC) [file pcbi.1003533.s022.doc]

Table S5 Rate expressions used for the stochastic simulations of the genetic toggle switch.

| Rxn | Rate Expression | h_i_ | Description of rate |
| --- | --- | --- | --- |
| 1 | h_1_*R1 | α_1_*V | Basal production promoter 1 |
| 2 | h_2_*R1 | β_1_/(1+R2/V)^n_1_) | Repressed production promoter 1 |
| 3 | -h_3_*R1 | D*R1 | Degradation |
| 4 | h_4_*R1-h_4_[R1:L1] | k_off_*[R1:L1] | Unbinding from load |
| 5 | -h_5_*R1+h_5_[R1:L1] | k_on_*R1 | Binding to load |
| 6 | h_6_*R2 | α_2_*V | Basal production promoter 2 |
| 7 | h_7_*R2 | β_2_/(1+R2/V)^n_2_) | Repressed production promoter 2 |
| 8 | -h_8_*R2 | D*R2 | Degradation |
| 9 | h_9_*R2-h_9_[R2:L2] | k_off2_*[R2:L2] | Unbinding from load |
| 10 | -h_10_*R2+h_10_[R2:L2] | k_on2_*R2 | Binding to load |

The rate expressions used for the stochastic simulation of the toggle switch along with the description of the reaction are shown.
