# Supplementary material for: Loads Bias Genetic and Signaling Switches in Synthetic and Natural Systems
Source: PLoS Comput Biol. 2014 Mar 27;10(3):e1003533. doi: 10.1371/journal.pcbi.1003533 (PMC3967935; doi:10.1371/journal.pcbi.1003533)
Supplement: Table S6 — List of reactions in the minimal model of Ras activation. The reactions in the minimal model of Ras activation, along with the labels of the corresponding rate constants are shown. Parameters used in the simulations are given in Table S7. (DOCX) [file pcbi.1003533.s023.docx]

Table S6. List of reactions in the minimal model of Ras activation.

| $Reactions$ | *S No.* |
| --- | --- |
| $SOScat+RasGDP \underset{\leftrightarrow}{kon1, koff1} SOScat(RasGDP)$ | *[R1]* |
| $SOScat+RasGTP \underset{\leftrightarrow}{kon2, koff2} SOScat(RasGTP)$ | *[R2]* |
| $SOScat(RasGTP)+RasGDP \underset{\leftrightarrow}{kon3, koff3}SOScat\left( RasGTP \right):RasGDP \underset{\to}{kcat3} SOScat(RasGTP)+RasGTP$ | *[R3]* |
| $SOScat(RasGDP)+RasGDP \underset{\leftrightarrow}{kon4, koff4}SOScat(RasGDP):RasGDP \underset{\to}{kcat4} SOScat(RasGDP)+RasGTP$ | *[R4]* |
| $RasGAP+RasGTP \underset{\leftrightarrow}{kon5, koff5} RasGAP:RasGTP \underset{\to}{kcat5} RasGAP+RasGDP$ | *[R5]* |
| $RasGTP+Raf \underset{\leftrightarrow}{kon6, koff6} RasGTP:Raf$ | *[R6]* |

The reactions in the minimal model of Ras activation, along with the labels of the corresponding rate constants are shown. Parameters used in the simulations are given in Table S7.
