# Supplementary material for: Loads Bias Genetic and Signaling Switches in Synthetic and Natural Systems
Source: PLoS Comput Biol. 2014 Mar 27;10(3):e1003533. doi: 10.1371/journal.pcbi.1003533 (PMC3967935; doi:10.1371/journal.pcbi.1003533)
Supplement: Table S7 — Kinetic rate parameters used for the simulations of the Ras model. Here the numbers in the subscript of the rate constants in the “Constant” column refer to the reactions shown in the corresponding row of Supplementary Table S6. The meaning of the rate constants are as follows: kon refers to the on-rate, koff is the off rate and kcat is the catalytic rate. The sources for the rates are as shown in the last column. (DOC) [file pcbi.1003533.s024.doc]

Table S7 Kinetic rate parameters used for the simulations of the Ras model.

| Rxn | Constant | 3D Rate Values | Units | 2D Rate Values | Units | Reference |
| --- | --- | --- | --- | --- | --- | --- |
| 1 | *k_on1_* | 0.12 | µM^-1^ s^-1^ | 0.028 | Molecules^-1^ s^-1^ | [2] |
| 1 | *k_off1_* | 3.0 | s^-1^ | 3.0 | s^-1^ | [2] |
| 2 | *k_on2_* | 0.11 | µM^-1^ s^-1^ | 0.026 | Molecules^-1^ s^-1^ | [2] |
| 2 | *k_off2_* | 0.4 | s^-1^ | 0.4 | s^-1^ | [2] |
| 3 | *k_on3_* | 0.05 | µM^-1^ s^-1^ | 0.0118 | Molecules^-1^ s^-1^ | [2] |
| 3 | *k_off3_* | 0.1 | s^-1^ | 0.1 | s^-1^ | [2] |
| 3 | *k_cat3_* | 0.038 | s^-1^ | 0.038 | s^-1^ | [2] |
| 4 | *k_on4_* | 0.07 | µM^-1^ s^-1^ | 0.0165 | Molecules^-1^ s^-1^ | [2] |
| 4 | *k_off4_* | 1.0 | s^-1^ | 1.0 | s^-1^ | [2] |
| 4 | *k_cat4_* | 0.003 | s^-1^ | 0.003 | s^-1^ | [2] |
| 5 | *k_on5_* | 1.74 | µM^-1^ s^-1^ | 0.41 | Molecules^-1^ s^-1^ | [2] |
| 5 | *k_off5_* | 0.2 | s^-1^ | 0.2 | s^-1^ | [2] |
| 5 | *k_cat5_* | 0.1 | s^-1^ | 0.1 | s^-1^ | [2] |
| 6 | *k_on6_* | 29.6e6 | M^-1^ s^-1^ | 6.96 | Molecules^-1^ s^-1^ | [3] |
| 6 | *k_off6_* | 5.22 | s^-1^ | 5.22 | s^-1^ | [3] |

Here the numbers in the subscript of the rate constants in the “Constant” column refer to the reactions shown in the corresponding row of Supplementary Table S6. The meaning of the rate constants are as follows: k_on_ refers to the on-rate, k_off_ is the off rate and k_cat_ is the catalytic rate. The sources for the rates are as shown in the last column.
