# Supplementary material for: Loads Bias Genetic and Signaling Switches in Synthetic and Natural Systems
Source: PLoS Comput Biol. 2014 Mar 27;10(3):e1003533. doi: 10.1371/journal.pcbi.1003533 (PMC3967935; doi:10.1371/journal.pcbi.1003533)
Supplement: Table S8 — List of reactions in the toy model of genetic toggle switch. The reactions in the toy model of the genetic toggle switch, discussed in Supplementary Text S1 section 3.1 are listed. The description of the various chemical species in the reactions are also provided in the Supplementary Text S1. (DOCX) [file pcbi.1003533.s025.docx]

Table S8. List of reactions in the toy model of genetic toggle switch.

| *Reactions* | *Index* |
| --- | --- |
| $AR_{1}$ *Module* |  |
|  | *P1* |
|  | *P2* |
|  | *P3* |
|  | *P4* |
|  | *P5* |
|  | *P6* |
|  | *P7* |
| $AR_{2}$ *Module* |  |
|  | *P8* |
|  | *P9* |
|  | *P10* |
|  | *P11* |
|  | *P12* |
|  | *P13* |
|  | *P14* |

The reactions in the toy model of the genetic toggle switch discussed in Supplementary Text. The description of the various chemical species are also provided in Supplementary Text.
