# Supplementary material for: Loads Bias Genetic and Signaling Switches in Synthetic and Natural Systems
Source: PLoS Comput Biol. 2014 Mar 27;10(3):e1003533. doi: 10.1371/journal.pcbi.1003533 (PMC3967935; doi:10.1371/journal.pcbi.1003533)
Supplement: Text S1 — Supporting Information including derivation and analysis of toggle switch and Ras models, parameters used for the simulation and their sources, results of the parameter sensitivity analysis, details of the effect of a dynamic load on the genetic toggle switch, results for the Ras model with Michealis-Menton kinetics, results for the models without the protection assumption. (PDF) [file pcbi.1003533.s026.pdf]

Supplementary Text

1. **Genetic Toggle Switch**
    1.1. Derivation and De-dimensionalization of Genetic Toggle Switch equations.
    1.2. The simulation box and concentrations for the stochastic simulations.
    1.3. Sensitivity to molecule number of the genetic toggle switch
    1.4. Parameter Sensitivity in Deterministic Simulations and Alternative Induction Method
        1.4.1. Alternative Induction Method
        1.4.2. Transition Time
        1.4.3. Inducer Required to Transition
    1.5. Effects of a Dynamic Load
    1.6. Positive Feedback on the Toggle Switch
        1.6.1. Derivation of Composite Promoter Term
        1.6.2. Strength of Positive Feedback
        1.6.3. Effect of Positive Feedback on Transition Times
2. **Ras System Model**
    2.1. Assumptions underlying the Ras Model
    2.2. Reactions modeled in Ras model
    2.3. ODE formulation
    2.4. Simulation box and parameters
    2.5. Results for both cases of Law of Mass Action (LMA) and Pseudo Steady State Assumption (PSSA)
    2.6. Parameter Sensitivity Analysis (PSA)
3. **Discussion on protection assumption with toggle switch and Ras Model**
    3.1. Toy Toggle Switch model
        3.1.1. Assumptions
        3.1.2. ODE model
        3.1.3. Results
    3.2. Toggle Switch without and with Positive Feedback Motif
        3.2.1. Modifications to original models
        3.2.2. Results
        3.2.3. Transition times in the absence of protection
    3.3. Ras Model
        3.3.1. Modifications to original model
        3.3.2. Results

1. Genetic Toggle Switch

1.1. Derivation and Dedimensionalization of Genetic Toggle Switch

Let $[R_1]$, $[R_2]$ be the concentration repressors 1 and 2. Let $[L_{1F}]$ and $[L_{2F}]$ be the concentration of unbound load 1 and 2 with total load concentration $[L_{1T}]$ and $[L_{2T}]$, and which can bind R1 and R2 reversibly. Let $[C_1]$ and $[C_2]$ be the concentration of a load-receptor complex.

$$\left[R_1\right]+\left[L_{1F}\right]\underset{k_{off1}}{\overset{k_{on1}}{\rightleftarrows}}\left[C_1\right]$$

$$\left[R_2\right]+\left[L_{2F}\right]\underset{k_{off2}}{\overset{k_{on2}}{\rightleftarrows}}\left[C_2\right]$$

R_1 is produced at a maximal rate of β_1 and is repressed by R_2. R_2 is produced at a maximal rate of β_2 and repressed by R_1. R_1 and R_2 degrade as a first order process at rate δ. Thus the differential equations governing $[R_1]$ and $[R_2]$ are:

$$\frac{d[R_1]}{dt} = \frac{\beta_1}{1+\left([R_2]/k_2\right)^n} - \delta[R_1] - k_{on1}[R_1][L_{1F}] + k_{off1}[C_1]$$

$$\frac{d[R_2]}{dt} = \frac{\beta_2}{1+\left([R_1]/k_1\right)^n} - \delta[R_2] - k_{on2}[R_2][L_{2F}] + k_{off2}[C_2]$$

We now de-dimensionalize these equations and define the following parameters. L_{10}, L_{20} are total amount of load 1 and 2 respectively.

$$u = [R_1]/k_1 \qquad v = [R_2]/k_2 \qquad \tau = t\delta$$

$$\ell_1 = [L_{1F}]/[L_{1T}] \qquad [C_1] = [L_{1T}] - [L_{1F}] \quad C_1' = [L_{1T}](1-\ell_1)$$

$$\ell_2 = [L_{2F}]/[L_{2T}] \qquad [C_2] = [L_{2T}] - [L_{2F}] \quad C_2' = [L_{2T}](1-\ell_2)$$

$$\frac{d\delta k_1 u}{d\tau} = \frac{\beta_1}{1+v^n} - \delta k_1 u - k_{on1}k_1 u \ell_1 L_{1T} + k_{off1}L_{1T}(1-\ell_1)$$

$$\frac{d\delta k_2 v}{d\tau} = \frac{\beta_2}{1+u^n} - \delta k_2 v - k_{on2}k_2 v \ell_2 L_{2T} + k_{off2}L_{2T}(1-\ell_2)$$

These equations reduce to:

$$\frac{du}{d\tau} = \frac{\beta_1/\delta k_1}{1+v^n} - u - \frac{k_{on1}[L_{1T}]}{\delta}u\ell_1 + \frac{k_{off1}[L_{1T}]}{\delta k_1}(1-\ell_1)$$

$$\frac{dv}{d\tau} = \frac{\beta_2/\delta k_2}{1+u^n} - v - \frac{k_{on2}[L_{2T}]}{\delta}v\ell_2 + \frac{k_{off2}[L_{2T}]}{\delta k_2}(1-\ell_2)$$

We now redefine several parameters and add a basal production rate of u and v, α_1 and α_2:

$$\beta_1' = \beta_1/\delta k_1 \qquad k_{on1}' = \frac{k_{on1}}{\delta} \qquad k_{off1}' = \frac{k_{off1}}{\delta k_1}$$

$$\beta_2' = \beta_2/\delta k_2 \qquad k_{on2}' = \frac{k_{on2}}{\delta} \qquad k_{off2}' = \frac{k_{off2}}{\delta k_2}$$

The final differential equations are therefore:

$$\frac{du}{d\tau} = \alpha_1 + \frac{\beta_1'}{1+v^n} - u - k_{on1}'[L_{1T}]u\ell_1 + k_{off1}'[L_{1T}](1-\ell_1) \tag{S1}$$

$$\frac{dv}{d\tau} = \alpha_2 + \frac{\beta_2'}{1+u^n} - v - k_{on2}'[L_{2T}]v\ell_2 + k_{off2}'[L_{2T}](1-\ell_2) \tag{S2}$$

$$\frac{d\ell_1}{d\tau} = -k_{on1}'k_1 u\ell_1 + k_{off1}'k_1(1-\ell_1) \tag{S3}$$

$$\frac{d\ell_2}{d\tau} = -k_{on2}'k_2 v\ell_2 + k_{off2}'k_2\left(1-\ell_2\right) \quad \text{(S4)}$$

$\alpha_1 = \alpha_2 = 0.2$; $\beta_1' = \beta_2' = 4$; $n=3$; $k_{on1}' = k_{on2}' = 0.5$; $k_{off1}' = k_{off2}' = 0.5$; $k_1 = k_2 = 1$; $[L_{1T}]$ and $[L_{2T}]$ are variable. Note that the bifurcation analysis presented later in the Supplementary figures demonstrates that the various models that we study possess at least one stable state, i.e. the Jacobian of the system has eigenvalues with all negative real parts at the fixed points, and hence are asymptotically stable (see for example Theorem 4.6, p121 in "Differential Dynamic Systems" by Meiss). Thus all models discussed can be assumed to show convergence to equilibrium.

1.2. The simulation box and concentrations for the stochastic simulations.

We used a volume of 1 μm^3 for the simulation box and the base parameters for simulation of the genetic toggle switch as in the deterministic simulations. This corresponded to small numbers of about 10-20 molecules of the two repressors in the simulation box. The small numbers of molecules led to frequent stochastic events and many spontaneous transitions between the two states. The rate expressions used for the stochastic simulations of the genetic toggle switch are shown in Table S5.

1.3. Sensitivity to molecule number of the genetic toggle switch

In order to test whether our procedure for constructing the quasi-potential landscape for the genetic toggle switch was robust to larger molecule numbers, we ran the simulation with the average number of molecules in each state about 5 times larger than that reported in the text. As shown in Figure S4, the trends are similar to those reported in the main paper. Note that the number of transitions seen in any block of time is much fewer and hence it takes a significantly longer computational time to actually collect enough data for smooth and accurate plots. However there is no qualitative change in the results due to a larger molecule number.

1.4. Parameter Sensitivity in Deterministic Simulations and Alternative Induction Method

1.4.1. Alternative Induction Method

We considered a second method of induction that utilizes an inducer that directly reduces the level of a repressor. We derived similar a system of four differential equations listed below:

$$\frac{du}{d\tau} = \alpha_1 + \frac{\beta_1'}{1+v^n} - \left(u + \frac{\gamma_3 I_1}{1+I_1}\right) - k_{on1}'\left[L_{1T}\right]u\ell_1 + k_{off1}'\left[L_{1T}\right]\left(1-\ell_1\right) \quad \text{(S5)}$$

$$\frac{dv}{d\tau} = \alpha_2 + \frac{\beta_2'}{1+u^n} - \left(v + \frac{\gamma_4 I_2}{1+I_2}\right) - k_{on2}'\left[L_{2T}\right]v\ell_2 + k_{off2}'\left[L_{2T}\right]\left(1-\ell_2\right) \quad \text{(S6)}$$

$$\frac{d\ell_1}{d\tau} = -k_{on1}'k_1 u\ell_1 + k_{off1}'k_1\left(1-\ell_1\right) \quad \text{(S7)}$$

$$\frac{d\ell_2}{d\tau} = -k_{on2}{}' k_2 v\ell_2 + k_{off2}{}' k_2 \left(1-\ell_2\right) \quad \text{(S8)}$$

γ_3 and γ_4 represent the activities of a factor (I_1 or I_2) that degrades R1 or R2, similar to the degradation of λ CI by RecA, modeled as in [1].
Because the qualitative results for both transition time and inducer required to transition did not vary with induction method, we report the results for the second method below in this Supplementary Text.

1.4.2. Transition Time

The relationship between amount of load and transition time was found to be linear across a range of parameters and both induction methods. This was true for a range of load binding on rates from K_{on}= 5 to 0.005. Of note is the identification of an optimal K_d for load binding which results in maximal effect on transition time. This can be seen in Figure S1. The effects of a low, medium and high K_d is demonstrated in Figure S2. We additionally varied β across two orders of magnitude; the relationship between transition time and load was found to be linear as shown in Table S1.

1.4.3. Inducer Required to Transition

The inducer decay rate, d_{I1}, affects the exponential parameter. As decay rate increases, more inducer is required to transition because the inducer persists in the system for less time. The exponential relationship can actually be written as a function of the decay rate: Inducer=C*exp(k*d_I*Load). This fact shows that the exponential relationship between the amount of inducer required to transition states and the load applied to the system is dependent upon the decay rate of the inducer. This procedure was repeated for both induction methods resulting in similar qualitative results. The results are shown in Tables S2 and S3.

1.5. Effects of a Dynamic Load

We explored the possibility that a load was not present in a constant amount but rather varied as the load was created and degraded. The equations used for a dynamic load are:

$$\frac{du}{d\tau} = \alpha_1 + \frac{\beta_1'}{1+v^n} - u - k_{on1}{}' \frac{k_{b1}}{k_{d1}} u\ell_1 + k_{off1}{}' \frac{k_{b1}}{k_{d1}} \left(c_1\right) \quad \text{(S9)}$$

$$\frac{dv}{d\tau} = \alpha_2 + \frac{\beta_2'}{1+u^n} - v - k_{on2}{}' \frac{k_{b2}}{k_{d2}} v\ell_2 + k_{off2}{}' \frac{k_{b2}}{k_{d2}} \left(c_2\right) \quad \text{(S10)}$$

$$\frac{d\ell_1}{d\tau} = -k_{on1}{}' k_1 \frac{k_{b2}}{k_{d2}} u\ell_1 + k_{off1}{}' k_1 \frac{k_{b1}}{k_{d1}} \left(c_1\right) + \frac{k_{d1}}{\delta} - \frac{k_{d1}}{\delta} \ell_1 \quad \text{(S11)}$$

$$\frac{d\ell_2}{d\tau} = -k_{on2}'k_2\frac{k_{b2}}{k_{d2}}v\ell_2 + k_{off2}'k_2\frac{k_{b2}}{k_{d2}}(c_2) + \frac{k_{d2}}{\delta} - \frac{k_{d2}}{\delta}\ell_2 \tag{S12}$$

$$\frac{dc_1}{d\tau} = k_{on1}'k_1 u\ell_1 - k_{off1}'k_1 c_1 \tag{S13}$$

$$\frac{dc_2}{d\tau} = k_{on2}'k_2 u\ell_2 - k_{off2}'k_2 c_2 \tag{S14}$$

Where k_{b1} is the creation rate of load 1 and k_{d1} is the degradation rate. K_{eq1}, defined as k_{b1}/k_{d1} was chosen as the de-dimensionalization constant (similar to L_{1T}, L_{2T} used above). As a result, the transition times were plotted against K_{eq} rather than L_T. The default parameter values used were: k_{d1}=k_{d2}=0.5; k_1=k_2=1; δ=1; k_{b1} and k_{b2} were varied from 0.5 to 50 to cover a range of loading conditions. In addition to the default parameters, we tested the effects of k_d, k_1, k_{on} and k_{off} on the relationship between load and transition time. Because the transitions between states are not induced until the system has reached a steady state, there was no qualitative effect on the deterministic results. The relationship between K_{eq} for the load and transition time was found to be linear in all parameter regimes. This is shown for the default parameter conditions in Figure S3 and for the other parameter conditions in Table S4.

1.6. Positive Feedback on the Toggle Switch

1.6.1. Derivation of Composite Promoter Term

Let *P* be a constitutively active promoter which produces repressor R_1 at rate β:

$$P \xrightarrow{\beta} P + R_1$$

Let R_1 have positive feedback on *P*:

$$P + R_1 \underset{k_2}{\overset{k_1}{\rightleftarrows}} PR_1 \xrightarrow{\rho} PR_1 + R_1$$

$$P + R_1 \xleftarrow{k_2} PR_1$$

Let R_1 be a repressor of *P* which binds in n copies:

$$P + nR_2 \underset{k_4}{\overset{k_3}{\rightleftarrows}} PnR_2$$

We assume quasi-steady state:

$$\frac{d[PR_1]}{dt} = k_1[P][R_1] - k_2[PR_1] = 0$$

$$\frac{d[PR_2]}{dt} = k_3[P][R_2]^n - k_4[PR_2] = 0$$

Therefore:

$$[PR_1] = \frac{k_1}{k_2}[P][R_1];\ [PR_2] = \frac{k_3}{k_4}[P][R_2]^n$$

We assume a constant amount of P. From the law of conservation:

$$P_0 = [P] + [PR_1] + [PnR_2]$$

Let $k_1/k_2 = k'$ and $k_3/k_4 = k''$

$$P_0 = [P] + k'[P][R_1] + k''[P][R_2]^n$$

$$[P] = \frac{P_0}{1 + k'[R_1] + k''[R_2]^n}$$

We now solve for the rate of R1:

$$\frac{d[R_1]}{dt} = \rho[PR_1] - k_1[P][R_1] + k_2[PR_1] + \beta P = \rho k'[P][R_1] + \beta P$$

$$\frac{d[R_1]}{dt} = [P](\rho k'[R_1] + \beta) = \frac{P_0 \rho k'[R_1] + P_0 \beta}{1 + k'[R_1] + k''[R_2]^n}$$

Let $\rho' = P_0 \rho k'$ and $\beta' = P_0 \beta$

$$\frac{d[R_1]}{dt} = \frac{\rho'[R_1] + \beta'}{1 + k'[R_1] + k''[R_2]^n}$$

This yields the positive feedback term in Eq. 10 in the text.

1.6.2. Strength of Positive Feedback

To assess the effects of a positive feedback on repressor 1, we tested various values of parameter *ρ*, the strength of positive feedback. First note that the positive feedback moiety, even in the presence of the load, does not abrogate bistability, unless *ρ* is very large, as shown in Figure S17. We then tested the probability distribution functions for the toggle with positive feedback without a load. The results of this are shown in Figure S6. When the positive feedback is 0, the probability distribution function for R1 and R2 is perfectly balanced. As the strength of positive feedback increases from 0 to 5, the pdf is increasingly skewed to R1. When *ρ*=5, the effects of the positive feedback are so strong that the system never switches stochastically into R2. As shown in the paper, this effect may be overcome by increasing the load on R1.

1.6.3. Effect of Positive Feedback on Transition Time

Even in the case of a positive feedback, the relationship between transition time and load remains linear. We explored the effect of transition time when the positive feedback was applied to the R1 and R2. In all cases, the relationship was linear. This is shown in Supplementary Figure S5.

2. Motivations of the Ras System Model

2.1. Assumptions underlying the Ras Model:

The model we used for the Ras-Kinase system is mainly adopted from the minimal model of the Ras Switch proposed by Das et. al. [1] In the following section, we briefly discuss the underlying assumptions of the minimal model of Ras Switch:

1. SOS: As in [1], only SOS (Son of Sevenless) family of Ras Guanine Nucleotide Exchange Factors (GEFs) is included in the model. The RasGRP (Ras Guanine Nucleotide Release Protein) family (including RasGRP1 and RasGRP2) which are also GEFs are not included. SOS is ubiquitously distributed, while RasGRP family is restricted to the nervous and hematopoietic systems.
2. SOScat: Not all the domains of SOS are taken into consideration in this model. Cdc25 and REM, together named as SOScat, are only two included, which are essential domains for GEF catalytic activities. The domains flanking SOScat, both N-terminal and C-terminal, are shown

to be inhibitory to GEF activities. *In vivo,* when SOS is recruited to the plasma membrane, the resulting conformational changes release this inhibition. In this minimal model this inhibiting effect is not modeled. For our purpose of investigating the effects of adding loads to the positive feedback based bistable Ras switch, we also only consider SOScat in our model.

3. SOS basal GEF activity: The original GEF activity of SOS is very low. However, its GEF activity is strongly influenced by the allosteric pocket in REM domain. When RasGDP binds to this pocket a 5-fold increase is observed in its GEF activity, while binding of RasGTP to this site results in an increase of 75 times. Based on the main aim of the paper, we also choose to neglect the original GEF activity of SOScat. However, we also tested this assumption by including this basal GEF activity in the Ras model, whose behaviors show no qualitative differences with the model we described in the main text (data not shown).
4. Intrinsic GTPase activity of Ras: intrinsic GTPase activity of Ras is relatively low. Proteins we have generically called RasGAPs are constitutively present that promote Ras deactivation from RasGTP into RasGDP. For simplicity the intrinsic GTPase activity of Ras is neglected and the enhanced deactivation of RasGTP by RasGAP is modeled as an enzymatic reaction.
5. Truncated Raf cascade: after RasGTP binds to Raf *in vivo*, Raf will be phosphorylated and activated by the RasGTP:Raf complex. Then the activated Raf proteins activate the RAF-MEK-ERK-CD69 pathway. However, for the purpose of our study here, Raf is simplified into only a binding partner of RasGTP. Thus, the downstream phosphorylation and activation steps are not considered.
6. Since we are interested in the short-term behavior of the system, no synthesis or degradation dynamics is considered in our model, i.e. total amounts of all primary molecules (SOScat, Ras protein, RasGAP, Raf) are conserved. Note that experiments show Ras activation peaking in one or two minutes after activation [1,2].
7. All enzymatic reactions are modeled by sequential reactions of enzyme (E) and substrate (S) firstly bind together to form enzyme:substrate complex (ES) with a reaction rate constant of $k_{on(i)}$, then the complex disassociates reversibly with $k_{off(i)}$ or produces the product (P) irreversibly with $k_{cat(i)}$. These reactions are shown schematically as:

$$E + S \underset{koff(i)}{\overset{kon(i)}{\rightleftarrows}} ES \xrightarrow{kcat(i)} P$$

2.2. Reactions modeled in Ras model

Based on the abovementioned assumptions, all reactions considered in our model are listed in Table S6. In particular, [R1] and [R2] describe the allosteric pocket reactions of binding and unbinding reactions between RasGDP/RasGTP and the allosteric pocket in SOS REM domain. [R3] and [R4] describe the reactions catalyzed by GEF pocket of SOScat with allosteric pocket occupied by RasGTP and RasGDP, indicated by SOS(RasGTP) and SOS(RasGDP) correspondingly. [R5] describes the RasGTP deactivation reaction into RasGDP enhanced by RasGAP. The last but not least, [R6] describes the binding and unbinding of RasGTP and Raf. An underlying assumption here is the protection model, where RasGTP is assumed to be free from deactivation of RasGAP after being bound to Raf. We refer the reader to a later section where this assumption is relaxed.

2.3. ODE formulation

To be more general, we use Law of Mass Action (LMA) to model all the rates of reactions listed in Supplementary Table S6. Then, the following set of ODEs is achieved for the changing rate of each of the involving species, which we will call the LMA model. For the following sections, we use the following notations for the species involved in the system:

$$x_1 \equiv [SOScat];\ x_2 \equiv [RasGDP];\ x_3 \equiv [RasGTP];\ x_4 \equiv [SOScat(RasGDP)];$$
$$x_5 \equiv [SOScat(RasGTP)];\ x_6 \equiv [SOScat(RasGDP):RasGDP];$$
$$x_7 \equiv [SOScat(RasGTP):RasGDP];\ x_8 \equiv [RasGAP];\ x_9 \equiv [RasGAP:RasGTP];$$
$$x_{10} \equiv [Raf];\ x_{11} \equiv [RasGTP:Raf]$$

$$\frac{dx_1}{dt} = -k_{on1}x_1x_2 + k_{off1}x_4 - k_{on2}x_1x_3 + k_{off2}x_5 \quad \text{(S15)}$$

$$\frac{dx_2}{dt} = -k_{on1}x_1x_2 + k_{off1}x_4 - k_{on3}x_2x_5 + k_{off3}x_7 - k_{on4}x_2x_4 + k_{off4}x_6 + k_{cat5}x_9 \quad \text{(S16)}$$

$$\frac{dx_3}{dt} = -k_{on2}x_1x_3 + k_{off2}x_5 + k_{cat3}x_7 + k_{cat4}x_6 - k_{on5}x_3x_8 + k_{off5}x_9 - k_{on6}x_3x_{10} + k_{off6}x_{11} \quad \text{(S17)}$$

$$\frac{dx_4}{dt} = k_{on1}x_1x_2 - k_{off1}x_4 - k_{on4}x_2x_4 + k_{off4}x_6 + k_{cat4}x_6 \quad \text{(S18)}$$

$$\frac{dx_5}{dt} = k_{on2}x_1x_3 - k_{off2}x_5 - k_{on3}x_2x_5 + k_{off3}x_7 + k_{cat3}x_7 \quad \text{(S19)}$$

$$\frac{dx_6}{dt} = k_{on4}x_2x_4 - k_{off4}x_6 - k_{cat4}x_6 \quad \text{(S20)}$$

$$\frac{dx_7}{dt} = k_{on3}x_2x_5 - k_{off3}x_7 - k_{cat3}x_7 \quad \text{(S21)}$$

$$\frac{dx_8}{dt} = -k_{on5}x_3x_8 + k_{off5}x_9 + k_{cat5}x_9 \quad \text{(S22)}$$

$$\frac{dx_9}{dt} = k_{on5}x_3x_8 - k_{off5}x_9 - k_{cat5}x_9 \quad \text{(S23)}$$

$$\frac{dx_{10}}{dt} = -k_{on6}x_3x_{10} + k_{off6}x_{11} \quad \text{(S24)}$$

$$\frac{dx_{11}}{dt} = k_{on6}x_3x_{10} - k_{off6}x_{11} \quad \text{(S25)}$$

The followings are conservation laws for primary molecules (SOS, Ras, GAP and Raf) involved in the system.

$$SOS_T = x_1 + x_4 + x_5 + x_6 + x_7 \quad \text{(S26)}$$

$$Ras_T = x_2 + x_3 + x_4 + x_5 + 2x_6 + 2x_7 + x_9 + x_{11} \quad \text{(S27)}$$

$$GAP_T = x_8 + x_9 \quad \text{(S28)}$$

$$Raf_T = x_{10} + x_{11} \quad \text{(S29)}$$

We can recover the equations used in Ref. [1] with: 1) classic Pseudo Steady State Assumption (PSSA) for all the time derivatives of enzyme-substrate complexes; 2) defining Michealis constants as $K_{(i)M} = (k_{off(i)} + K_{cat(i)})/k_{on(i)}$; 3) Approximations of conservation law by ignoring

enzyme-substrate complexes under PSSA. Then the enzymatic reaction rates can be simplified into classical Michealis Menten Kinetics and the overall set of ODEs simplified as:

$$x_1 \equiv \left[SOScat\right];\ x_2 \equiv \left[RasGDP\right];\ x_3 \equiv \left[RasGTP\right];\ x_4 \equiv \left[SOScat\left(RasGDP\right)\right];$$
$$x_5 \equiv \left[SOScat\left(RasGTP\right)\right];\ x_6 \equiv \left[SOScat\left(RasGDP\right):RasGDP\right];$$
$$x_7 \equiv \left[SOScat\left(RasGTP\right):RasGDP\right];\ x_8 \equiv \left[RasGAP\right];\ x_9 \equiv \left[RasGAP:RasGTP\right];$$
$$x_{10} \equiv \left[Raf\right];\ x_{11} \equiv \left[RasGTP:Raf\right]$$

$$\frac{dx_1}{dt} = -k_{on1}x_1x_2 + k_{off1}x_4 - k_{on2}x_1x_3 + k_{off2}x_5 \quad \text{(S30)}$$

$$\frac{dx_3}{dt} = -k_{on2}x_1x_3 + k_{off2}x_5 + \frac{k_{cat3}x_2x_5}{K_{m3}+x_2} + \frac{k_{cat4}x_2x_4}{K_{m4}+x_2} - \frac{k_{cat5}GAP_Tx_3}{K_{m5}+x_3} - k_{on6}x_3x_{10} + k_{off6}x_{11} \quad \text{(S31)}$$

$$\frac{dx_5}{dt} = k_{on2}x_1x_3 - k_{off2}x_5 \quad \text{(S32)}$$

$$\frac{dx_{11}}{dt} = k_{on6}x_3x_{10} - k_{off6}x_{11} \quad \text{(S33)}$$

The total molecular numbers of SOS, Ras and Raf are conserved in the PSSA system resulting in three additional conservation equations:

$$SOS_T = x_1 + x_4 + x_5 \quad \text{(S34)}$$

$$Ras_T = x_2 + x_3 + x_4 + x_5 + x_{11} \quad \text{(S35)}$$

$$Raf_T = x_{10} + x_{11} \quad \text{(S36)}$$

2.4. Simulation box and parameters

A quasi-two dimensional simulation box, similar to the one used by Das et al. [1] was utilized. Our simulation box is a 2μm by 2 μm surface with a height of 1.7 nm. Every molecular species is assumed to be well-mixed in this box.

Reaction rate constants were referenced from [1] and [3]. In our analysis, molecular concentrations were converted to molecular numbers in the simulation box for both deterministic and stochastic analyses. Thus, reaction rate constants need to be converted accordingly. For our simulation box, all the reactions are assumed to happen in the membrane area, which can be considered as two-dimensional. Therefore, the reaction rate constants need conversions not only from concentrations to molecular numbers, but also from 3D to 2D. Based on the simulation box used, a factor of 0.941/4 is used for the conversion from 3D rate constants with unit of (μM^{-1} s^{-1}) to 2D rate constants with unit of ($Molecules^{-1}$ s^{-1}). All the parameters we used in our deterministic and stochastic studies are listed in Supplementary Table 7 with both 3D and 2D values.

For all the following studies, 75 molecules of Ras, 6 molecules of RasGAP were used unless otherwise indicated.

2.5. Results for both cases of LMA and PSSA

Results for both the case of LMA and PSSA are shown in Figure 9 (main text) and Figure S7 correspondingly. For the case of LMA, the red line shows the bifurcation analysis results for the Ras system without Raf inside. A bistable regime is observed. While adding more Raf molecules into the system, both limit points are shifting to the right, bistable regime is decreasing and maximal excitable level of RasGTP is decreasing. When more than 21 molecules of Raf are added, as shown by the curve of "Total Raf = 25", the bistable regime totally diminishes and for all values of SOScat only one monostable point of the system remains.
Supplementary Figure S7 shows the case of PSSA, similar pattern of the changes in bistable region can be observed as shown in Figure 9 (main text). The only difference between these two cases is for the case of PSSA slightly more Raf molecules are needed to achieve same effect.
To directly examine the effects of adding different amount of Raf into the Ras activation system for both LMA and PSSA models, we also carried out bifurcation analyses with total number of Raf, i.e. Raf_T as primary parameter, which are shown in Supplementary Figure S8 and S9 correspondingly. Both Supplementary Figure S8 and S9 start with bistable region when there is no Raf in the system as we can predict. With the increase of total number of Raf in the system, the values of "high" steady state decrease together with increase in the values of the unstable steady state. This results in vanishing of both steady states and only one monostable region after Raf_T reaches a certain threshold. Again, similar patterns are observed for both cases of LMA and PSSA, and the only difference between them is the scale.

2.6. Parameter Sensitivity Analysis (PSA)

For PSA of the Ras Minimal Model, we refer the reader to Das et. al. [1]. For our main purpose of investigating the load to the Ras Switch, we varied the values of k_{on6} and k_{off6} and check their influences on the bistability of the system. We first maintained the same ratio of k_{on6} to k_{off6}, then we changed this ratio and check individual influences.
When keep the ratio between k_{on6} and k_{off6} the same and vary absolute values of k_{on6} and k_{off6}, no difference in bifurcation diagram is noticed (data not shown). This means there are no changes in steady state values if the ratio between these two parameters is maintained.
Then k_{on6} and k_{off6} are varied separately. As shown in Figure S10, increase of k_{off6} results in left shifts of both limit points, increase in bistable regime and increase in maximal RasGTP activation level. Qualitative features of bistability are maintained. Decrease of k_{off6} results in right shift of both limit points, increase in unstable bistable regime and decrease in maximal RasGTP activation level. Qualitative features of bistability are also maintained.
Supplementary Figure S11 shows the results of an increase in k_{on6} value. This results in reverse effects as shown in Supplementary Figure S10. Noticeably, increase k_{off6} by 10 times has exactly the same effects as decrease k_{on6} by 10 times and *vice versa*. This indicates the key player of k_{on6} and k_{off6} in the Ras system is the value of their ratio and is additional assurance that no changes would be observed when the ratio between these two parameters is maintained.

3. Discussion on protection assumption with a toy toggle switch and Ras Model

Assumption of Protection Model

For both the toggle switch and the Ras Switch, we assumed that the output molecule is protected when bound with the Load. For the toggle switch model, we assumed the repressor proteins are free from first order degradation when bound in the repressor-load complex; for the Ras Switch model, we assumed RasGTP is relieved from enhanced GTPase activity by RasGAP in its complex form with Raf. We show the effects of removing this assumption in the main text. Here we report additional data.

The Hill function form of the genetic toggle we have used does not allow for explicit binding of the repressors with the promoters they repress. We also considered the question whether allowing the repressor to decay when bound with the promoter (note, not the load) would have an effect on the system. To elucidate this point, a toy model is proposed to make the lumped processes in the Toggle Switch model more explicit. We present below an LMA based model for the toggle switch which we use to test whether allowing the *decay of the repressor when bound to the promoter it represses* can has any effect on the system.

3.1. Toy Toggle Switch model

For this model, we construct a demonstrative classical toggle switch similar to the one discussed in the text, but using LMA. In this model system, two equivalent repressors are expressed by corresponding genes. Inactive repressors monomers then become activated after a trimerization process. Activated repressor trimmers can then bind to corresponding promoters and repress the transcriptions of the other repressor monomer. Without repressor bound to promoters, genes can be transcribed at full rate.

3.1.1. Assumptions

Several assumptions were made for this model to both meet our purpose and maintain it in an intuitive form.

1) Two sides of the toggle switch are identical, i.e. same reactions involved and same parameters for same reactions.
2) Since we are interested in the steady state behaviors of the model system, transcriptional and translational processes are lumped together into one overall reaction and assumed to happen immediately without delay.
3) Promoter values are approximated as continuous concentrations rather than more appropriate discrete number of sites.
4) "Leaky" transcriptions of the promoters when they are bound by corresponding repressors are ignored.
5) Repressor monomers and trimers are identical in their degrading dynamics, thus same degrading parameters are used.

Reactions included in this toggle switch model were then formulated and listed in Supplementary Table 8. Particularly, [P1] and [P8] describe the trimerization reactions of inactive repressor monomers ($R_{(i)}$) into active repressor trimers ($AR_{(i)}$). [P2] and [P9] describe the binding and unbinding reactions of active repressor trimers to corresponding promoters ($Pro_{(i)}$) to form repressor:promoter complex ($AR_{(i)}$: $Pro_{(i)}$). [P3] and [P10] describe the "ON state" of the promoters without being bound by repressor, which directly give birth to repressor monomers. [P4] and [P11] will be used to test the protection model, which describes the degradations of repressor trimers when they are bound to promoters. Degradation rate constants of this reaction will be set to zero for protection assumption as control and set equal to other degradation rate constant for our purpose. [P5], [P6], [P12] and [P13] are degradation reaction of both repressor monomers and trimers. [P7] and [P14] describe binding and unbinding reactions

between repressor monomers and load molecules ($L_{(i)}$) to form repressor:load complex ($R_{(i)}$:$L_{(i)}$).

3.1.2. ODE model

Following notations are used for the toggle switch model:

$$x_1 \equiv [R_1];\ x_2 \equiv [AR_1];\ x_3 \equiv [Pro_1];\ x_4 \equiv [AR_1 : Pro_1];\ x_5 \equiv [L_1];\ x_6 \equiv [R_1 : L_1]$$

$$x_7 \equiv [R_2];\ x_8 \equiv [AR_2];\ x_9 \equiv [Pro_2];\ x_{10} \equiv [AR_2 : Pro_2];\ x_{11} \equiv [L_2];$$

$$x_{12} \equiv [R_2 : L_2]$$

We use Law of Mass Action to formulate all the reaction rates and achieve the following time dependent ODEs for each species:

$$\frac{dx_1}{dt} = -3k_1x_1^3 + 3k_2x_2 + \alpha_1 x_9 - k_7x_1 - k_{on1}x_1x_5 + k_{off1}x_6 \quad \text{(S37)}$$

$$\frac{dx_2}{dt} = k_1x_1^3 - k_2x_2 - k_3x_2x_3 + k_4x_4 - k_6x_2 \quad \text{(S38)}$$

$$\frac{dx_3}{dt} = -k_3x_2x_3 + k_4x_4 + k_5x_4 \quad \text{(S39)}$$

$$\frac{dx_4}{dt} = k_3x_2x_3 - k_4x_4 - k_5x_4 \quad \text{(S40)}$$

$$\frac{dx_5}{dt} = -k_{on1}x_1x_5 + k_{off1}x_6 \quad \text{(S41)}$$

$$\frac{dx_6}{dt} = k_{on1}x_1x_5 - k_{off1}x_6 \quad \text{(S42)}$$

Since the toggle switch is symmetric, ODEs for the other side are identical except for indexes of variables and parameters thus not presented here. Also governing this system is the conservation of molecule numbers of promoters:

$$Pro_{1T} = x_3 + x_4 \quad \text{(S43)}$$

To generate Figure S12, following parameter values are used:

$k_1 = k_8 = 0.3$, $k_2 = k_9 = 10$, $k_3 = k_{10} = 0.6$, $k_4 = k_{11} = 1$, $k_5 = k_{12} = 0.1$, $k_6 = k_{13} = 0.1$, $k_7 = k_{14} = 0.1$, $k_{on} = 0.5$, $k_{off} = 0.2$.

3.1.3. Results

Supplementary Figure S12 shows the differences between the system with protection of repressor molecules from degradation when bound to promoter (note: not the load) and the one without. If the protection is not included, a very minor increase in the bistable region can be observed with right shift of upper limit point and left shift of lower limit point.

3.2. Toggle Switch without and with Positive Feedback Motif

3.2.1. Modifications to original models

If the protection assumption is released for the toggle switch model, two more reactions should be added into the reaction system.

$$C_1 \xrightarrow{\delta} L_{1F}$$

$$C_2 \xrightarrow{\delta} L_{2F}$$

Corresponding changes to de-dimensionalized ODEs should also be made:

$$\frac{dl_1}{dt} = -k_{on1}{}^{'}k_1 u l_1 + k_{off1}{}^{'}k_1\left(1-l_1\right) + \left(1-l_1\right) \quad \text{(S44)}$$

$$\frac{dl_2}{dt} = -k_{on2}{}^{'}k_2 v l_2 + k_{off2}{}^{'}k_2\left(1-l_2\right) + \left(1-l_2\right) \quad \text{(S45)}$$

When assuming steady state for the entire system, all the terms introduced by load molecules cannot be cancelled out as in the case of protection model. Thus, influences to steady-state behaviors by adding load molecules to the Toggle Switch system should be expected.

3.2.2. Results

Figure 2A shows the steady-state effects of adding increasing number of load molecules to both sides of the original genetic toggle switch. Without load molecule, the system is bistable with two stable steady states and one unstable steady state as predicted. With the increase of number of load molecules, all these three steady state become closer and finally meet together at certain level of L_T. Then two steady states vanish and only one stable steady state left. Figure 2B shows the steady-state effects of adding increasing number of load molecules to R1 side (L_{1T}) of the original genetic toggle switch. Without any load molecule in the system, the toggle switch is bistable with two stable steady states and one unstable steady state as predicted. Adding increasing number of load molecules results in becoming closer between the upper stable steady state and the unstable steady state. At certain threshold of L_{1T} these two steady states meet and vanish, with only the lower stable steady state left.

Supplementary Figure S13 shows effects of adding load molecules to the toggle switch with positive feedback on one side when the protection assumption is released. Interestingly, adding same amount of load molecules to different sides also cause different responses from the system. Adding load to R1 side results in increase of bistable regime and adding to R2 side results in decrease of bistable regime. When equal amount of loads added into both sides, the bistable regime is increased but to an extent smaller than adding to R1 side along. Increase in R1 level is much faster in this case than in Figure 2 due to the positive feedback loop.

3.2.3. Transition times in the absence of protection

The relationship between transition times and load is altered when the protection of a lower or absent decay rate of the repressor from the load complex. As predicted from Figure 2, the system with a one-sided load loses bistability with a load of 3.4; with a both sided load the system loses bistability with a load of 11. Thus we tested the transition times of the system within this regime. As shown in Figure 4, when a load is

applied to the same side, there is a positive linear relationship between rise time and load, but a negative linear relationship with decay time. Conversely, a load applied to the opposite side results in a negative linear relationship between rise time and load, but a positive linear relationship with decay time. A load applied to both sides result in a negative linear relationship for both rise time and decay time. This result is discussed in the main text.

3.3. Ras Model

For Ras model, protection model is also assumed implicitly, since RasGTP is free from GTPase activities after binding to Raf. In this section, we examine potential influences caused by this assumption. For the Ras Switch, we modified the original set of ODE's by including the RasGTP:Raf complexes as an equivalent substrate of RasGAP.

3.3.1. Modifications to original model

If RasGTP can still be deactivated into RasGDP by RasGAP in the complex form with Raf, one more reaction should be included into the Ras System:

$$\text{RasGAP+RasGTP:Raf} \underset{koff5}{\overset{kon5}{\longleftrightarrow}} \text{RasGAP:RasGTP:Raf} \xrightarrow{kcat5} \text{RasGAP+RasGDP+Raf}$$

$$\text{RasGAP} + \text{RasGTP} : \text{Raf} \overset{\text{kon5,koff5}}{\leftrightarrow} \text{RasGAP} : \text{RasGTP} : \text{Raf} \overset{\text{kcat5}}{\rightarrow} \text{RasGAP} + \text{RasGDP} + \text{Raf}$$

$$\text{RasGAP} + \text{RasGTP} : \text{Raf} \overset{\text{kon5,koff5}}{\leftrightarrow} \text{RasGAP} : \text{RasGTP} : \text{Raf} \overset{\text{kcat5}}{\rightarrow} \text{RasGAP} + \text{RasGDP} + \text{Raf}$$

The same reaction rate constants are assumed for the new reaction as free RasGTP deactivation. One more species is introduced into this system, i.e. $x_{12} \equiv \left[RasGAP : RasGTP : Raf \right]$. Based on this new reaction, several modifications should also be made for the ODEs system including adding more terms into x_2, x_8, x_{10} and x_{11} equations and add a new equation of x_{12}.

$$\frac{dx_2}{dt} = -k_{on1}x_1x_2 + k_{off1}x_4 - k_{on3}x_2x_5 + k_{off3}x_7 - k_{on4}x_2x_4 + k_{off4}x_6 + k_{cat5}x_9 + k_{cat5}x_{12} \quad \text{(S46)}$$

$$\frac{dx_8}{dt} = \dagger - k_{on5}x_3x_8 + k_{off5}x_9 + k_{cat5}x_9 - k_{on5}x_8x_{11} + k_{off5}x_{12} + k_{cat5}x_{12} \quad \text{(S47)}$$

$$\frac{dx_{10}}{dt} = -k_{on6}x_3x_{10} + k_{off6}x_{11} + k_{cat5}x_{12} \quad \text{(S48)}$$

$$\frac{dx_{11}}{dt} = k_{on6}x_3x_{10} - k_{off6}x_{11} - k_{on5}x_8x_{11} + k_{off5}x_{12} \quad \text{(S49)}$$

$$\frac{dx_{12}}{dt} = k_{on5}x_8x_{11} - k_{off5}x_{12} - k_{cat5}x_{12} \quad \text{(S50)}$$

Modifications are also needed for conservation laws:

$$Ras_T = x_2 + x_3 + x_4 + x_5 + 2x_6 + 2x_7 + x_9 + x_{11} + x_{12} \quad \text{(S51)}$$

$$GAP_T = x_8 + x_9 + x_{12} \quad \text{(S52)}$$

$$Raf_T = x_{10} + x_{11} + x_{12} \quad \text{(S53)}$$

3.3.2. Results

Figure S14 shows the changes of bifurcation curve with different numbers of Raf molecules (5, 15, 25 and 200) added into the system in logarithmic scale respectively. A similar pattern of decreases in bistable region and finally elimination of the bistable region as reported in the Figure 9 in main text is still observed but with a more complicated dynamics. Differences between the case without protection model and the one in main text as with protection model will be discussed as follows.

As shown in Figure 9 in the main text, where the protection model is included, maximal activation level of RasGTP is always decreasing with increase in Raf molecules added into the system. While in Supplementary Figure S14 reported here, maximal activation level of RasGTP first increase (as for the case of "Total Raf=5") and then decrease (comparing the case of "Total Raf=25" to "Total Raf = 15") with adding more Raf molecules.

Elimination of bistable region happens with much more Raf molecules. Even though the bistable region already decreased a lot after adding 25 molecules, the left bistable region needs much more Raf molecules to eliminate. Even with 150 Raf molecules, a tiny bistable region still exists. After around 200 molecules of Ras added, the bistability is abrogated.

Decrease in bistable region in Figure 9 in the main text is a result of right shifts of both fixed points with a faster rate of shift for the upper fixed points. While the decrease in Supplementary Figure S14 is a result of the leftward shift of both fixed points with a faster shift rate for the lower fixed point.

References:

1. Das J, Ho M, Zikherman J, Govern C, Yang M, et al. (2009) Digital signaling and hysteresis characterize ras activation in lymphoid cells. Cell 136: 337-351.
2. Prasad A, Zikherman J, Das J, Roose JP, Weiss A, et al. (2009) Origin of the sharp boundary that discriminates positive and negative selection of thymocytes. Proc Natl Acad Sci U S A 106: 528-533.
3. Kiel C, Serrano L (2009) Cell type-specific importance of ras-c-raf complex association rate constants for MAPK signaling. Science Signaling 2: ra38.
